# Incidence of Lung Adenocarcinoma by Age, Sex, and Smoking Status in Taiwan

**DOI:** 10.1001/jamanetworkopen.2023.40704

**Published:** 2023-11-01

**Authors:** Li-Hsin Chien, Hsin-Fang Jiang, Fang-Yu Tsai, Hsing-Yi Chang, Neal D. Freedman, Nathaniel Rothman, Qing Lan, Chao A. Hsiung, I-Shou Chang

**Affiliations:** 1 Institute of Population Health Sciences, National Health Research Institutes, Zhunan, Taiwan; 2 Department of Applied Mathematics, Chung-Yuan Christian University, Chung-Li, Taiwan; 3 National Institute of Cancer Research, National Health Research Institutes, Zhunan, Taiwan; 4 Division of Cancer Epidemiology and Genetics, National Cancer Institute, Bethesda, Maryland

## Abstract

**Importance:**

Knowing whether the effects of smoking and other risk factors with lung adenocarcinoma (ADC) incidence varies by sex would provide information on lung cancer prevention strategies.

**Objective:**

To evaluate whether women in Taiwan have higher age- and tumor stage–specific lung ADC incidence rates than men irrespective of smoking status (ie, ever smoker or never smoker).

**Design, Setting, and Participants:**

This population-based cohort study used data sets synthesized from the Taiwan Cancer Registry (TCR) from 1979 to 2019; the TCR Long Form (TCRLF) from 2011 to 2019, which provides individual-level smoking and tumor stage information; the Taiwan Cause of Death Database (TCOD) from 1985 to 2019; the National Health Insurance Research Database (NHIRD) from 2000 to 2020; the Monthly Bulletin of Interior Statistics (MBIS) from 2011 to 2019; the National Health Interview Survey from 2001, 2005, 2009, 2013, and 2017; and Taiwan Biobank data from 2008 to 2021. Included patients were aged 40 to 84 years and had any invasive lung cancer from January 1, 2011, to December 31, 2019.

**Exposure:**

Smoking status.

**Main Outcomes and Measures:**

The main outcomes were age-specific female-to-male incidence rate ratios (IRRs) of lung ADC by smoking status and tumor stage. Linked data from the TCR, TCOD, NHIRD, Taiwan National Health Interview Survey, and MBIS were used to estimate the age- and sex-specific numbers of cancer-free individuals at midyears from 2011 to 2019 by smoking status. Using the TCR and TCRLF, age-, sex-, tumor stage–, and diagnosis year–specific numbers of patients with lung ADC from 2011 to 2019 by smoking status were estimated.

**Results:**

A total of 61 285 patients (32 599 women [53.2%]) aged 40 to 84 years (mean [SD] age, 64.66 [10.79] years) in the Taiwanese population of approximately 23 million were diagnosed with invasive lung ADC as their first lifetime cancer between 2011 and 2019. Among smokers, men had higher tobacco use by almost all examined metrics, including nearly twice the mean (SD) number of pack-years smoked (eg, 7.87 [8.30] for men aged 30-34 years vs 4.38 [5.27] for women aged 30-34 years). For 5-year age bands between 40 and 84 years, incidence of lung ADC was significantly higher among females than males for nearly all age groups irrespective of tumor stage and smoking status (eg, for the age group 70-74 years, the female-to-male IRR for late-stage lung ADC among never smokers was 1.38 [95% CI, 1.30-1.50]).

**Conclusions and Relevance:**

In this cohort study, women had higher age- and stage-specific lung ADC incidence rates than men in Taiwan for both never and ever smokers, suggesting the possibility of differential exposures between sexes to risk factors other than smoking and the potential modification of ADC risk factors by sex. Further work is needed to determine whether this pattern replicates in other populations, discover the causes of lung ADC, and put preventive measures in place.

## Key Points

**Question:**

Do women have higher lung adenocarcinoma (ADC) rates than men in Taiwan regardless of smoking status?

**Findings:**

In this cohort study of 61 285 patients with lung ADC, incidence of lung ADC was significantly higher among females than among males irrespective of age, tumor stage, and smoking status.

**Meaning:**

The findings suggest a possible difference in lung ADC incidence by sex; there is a need to determine whether this is replicated in other populations and to evaluate the etiologic and translational implications.

## Teaser

This cohort study compares incidence rates of lung adenocarcinoma between men and women in Taiwan by smoking status, age, and disease stage.

## Introduction

Lung cancer is the most common incident cancer among men and the second most common cancer among women worldwide.^1^ Historically, lung cancer incidence rates have been higher among men than among women, reflecting higher smoking rates among men.^2^ Among ever smokers, men smoke more cigarettes and initiate smoking at an earlier age.^3,4^ However, emerging studies have observed a convergence of lung cancer incidence rates between sexes.^5,6^ In some populations, rates have even become higher among young women than among young men.^5,6^ In Western countries, where the age-specific smoking prevalence in women approached but did not exceed that of men, increasing lung adenocarcinoma (ADC) incidence rates have been observed, especially in women.^2,7^

Having reviewed risk factors for lung cancer, Fidler-Benaoudia and colleagues^6^ mentioned the possibility that women may be at an increased risk of lung cancer compared with men. Indeed, lifelong female never smokers have had slightly higher age-standardized rates of lung cancer than their male counterparts in population cohorts^8,9,10,11^; however, the case numbers in these studies were not large enough for age-specific studies. When smokers were compared with never smokers, some studies reported a higher relative risk for smoking females than for smoking males, while others found no significant difference.^11,12,13,14^ All these reports prompt the study of age- and sex-specific lung cancer incidence rates by smoking status.

In fact, such study of incidence rates is especially desirable and timely in East Asia in view of the recent observations that in East Asia, there exists a nearly simultaneous decrease in smoking rates, extensive introduction of low-dose computed tomography (LDCT) lung cancer screening, and a shift in lung cancer stage at diagnosis.^15,16,17,18,19,20^ These studies pointed out overdiagnosis due to LDCT lung cancer screening,^15,18^ an increase in incidence rates in females,^18^ and the relevance of the subtype ADC in this context.^18^ These findings reinforce the importance of the study of sex differences in lung ADC incidence and overdiagnosis by smoking status. To address some of these problems, we examined age-, sex-, and stage-specific lung ADC incidence rates by smoking status (ever or never smoker) in Taiwan.

## Methods

This population-based ecological cohort study followed the Strengthening the Reporting of Observational Studies in Epidemiology (STROBE) reporting guideline and was approved by the institutional review board of Taiwan’s National Health Research Institutes, which waived informed consent because all the data sets used were provided to us after deidentification. The study conformed to the Declaration of Helsinki provisions.^21^ All the analyses were performed in a secured area of the Data Science Center, Ministry of Health and Welfare, Taiwan.

We used data from 2011 to 2019 from the Taiwan Cancer Registry (TCR), including the long form (TCRLF), which collected individual-level smoking information for each patient with lung cancer starting in 2011; the Taiwan National Health Interview Survey (NHIS), which estimated age- and sex-specific ever smoking rates for 2001, 2005, 2009, 2013, and 2017; and the Taiwan Monthly Bulletin of Interior Statistics (MBIS), which reports age- and sex-specific population size. Because of case numbers, we report results for lung ADC more completely than for lung squamous cell carcinoma (SCC) and small cell lung cancer (SCLC) and excluded other histologic subtypes. Based on the NHIS, we estimated age- and sex-specific smoking rates in the Taiwanese population for each year between January 1, 2011, and December 31, 2019.

Linking the TCR from 1979 to 2019, the Taiwan Cause of Death Database (TCOD) from 1985 to 2019, and the National Health Insurance Research Database (NHIRD) from 2000 to 2020, we estimated the age- and sex-specific numbers of cancer survivors (patients diagnosed with any invasive cancer) in Taiwan at each midyear from January 1, 2011, to December 31, 2019; in general, an individual in the NHIRD at a certain time point, in the TCR before that time point, and not in the TCOD before that time point was considered a cancer survivor at that time point. Subtracting these individuals from the corresponding age- and sex-specific midyear population size reported in the MBIS and then multiplying by the corresponding smoking rates derived from the NHIS resulted in the age- and sex-specific numbers of cancer-free individuals for each midyear from 2011 to 2019 by smoking status. These data provided the person-years for estimating age-, sex-, and stage-specific lung ADC incidence rates by smoking status. The data sets, procedures, and estimates are detailed in the eMethods and eTables 1A to 5 in Supplement 1.

The quality of the TCR and TCRLF is high in terms of completeness, timeliness, and accuracy.^22,23^ The quality of the TCOD has been described elsewhere.^24^ The NHIRD is a valuable research resource in population health studies.^25^ Some of us have used the linkage of these 3 data sets in epidemiologic studies of cancer^26,27^ and in the prediction of lung cancer risk among ever smokers.^28^ The study design and quality of the NHIS was reported previously.^29,30^

Given a combination of age, sex, and diagnosis year, we identified the set of patients whose first invasive cancer was lung ADC and who were included in the TCR specified by the combination. Restricting this set to the subset of patients included in the TCRLF, we estimated the percentage of patients with late-stage (stages 2-4) lung ADC among all patients with stages 1 to 4 invasive lung ADC for the combination. We also used the TCRLF to estimate the percentage of ever smokers among the subset of patients with invasive lung ADC specified by each age, sex, stage, and diagnosis year combination. Combining all these data resulted in estimated age-, sex-, stage-, and diagnosis year–specific numbers of patients with lung ADC in the TCR by smoking status (detailed in the eMethods and eTables 6 to 8C in Supplement 1). For comparison, we also studied patients diagnosed with stage 3 or 4 lung ADC (eTable 8C in Supplement 1). eTable 9 in Supplement 1 reports the number of patients with invasive lung ADC in the TCR with individual-level smoking information from 2011 to 2019 using the TCRLF.

### Statistical Analysis

We used the estimated age-, sex-, and diagnosis year–specific numbers of patients with late-stage or early-stage lung ADC by smoking status (reported in eTables 8A and 8B in Supplement 1) and the corresponding person-years (reported in eTable 5 in Supplement 1) to obtain the age-, sex-, and stage-specific lung ADC incidence rates by smoking status and period (2011-2015, 2016-2019, and 2011-2019), shown in eTables 10A to 10C in Supplement 1 for late-stage, early-stage, and stages 3 and 4, respectively. We also obtained the incidence rate ratios (IRRs) comparing incidence rates between sexes by smoking status and period, between ever and never smokers by sex and period, and between periods by sex and smoking status, shown in eTables 11A to 11C in Supplement 1 for late-stage, early-stage, and stages 3 and 4, respectively. All 95% CIs were the intervals between the 2.5th percentiles and the 97.5th percentiles estimated using bootstrapping methods.^31^ The estimation procedures are detailed in the eMethods in Supplement 1 and were implemented using the boot package in R, version 4.3.0 (R Project for Statistical Computing). We sometimes used the binomial distribution to report a 95% CI or did not report a 95% CI when the estimand was too small; situations such as these are noted in the relevant tables. The data analyses were performed between March 1 and September 9, 2023.

We used the Taiwan Biobank from 2008 to 2021, including a total of 132 720 participants, to compare smoking exposures between sexes, including number of pack-years smoked, number of cigarettes smoked per day, number of years smoked, and years since cessation (eMethods and eTables 12A and 12B in Supplement 1). eTable 13 in Supplement 1 reports codes for lung ADC. For privacy protection consideration, the Data Science Center did not allow us to bring the number of patients specified by an age, sex, stage, histologic subtype, and smoking status combination out of the center for subsequent analyses if that number was between 1 and 4. This was the main reason that we limited our study to ADC, SCC, and SCLC and considered only age groups 40 to 64 years and 65 to 84 years for some results about early-stage SCC and SCLC.

With this in mind, we prepared corresponding tables for SCC and SCLC. Specifically, eTables 14 to 20 in Supplement 1 are the SCC counterparts of eTables 6 to 11 and 13 for ADC; eTables 21 to 27 in Supplement 1 are the SCLC counterparts. We also prepared corresponding eTables 28 to 33C in Supplement 1 for lung cancer including all the subtypes.

## Results

From 2011 to 2019, a total of 61 285 patients (28 686 men [46.8%]; 32 599 women [53.2%]) aged 40 to 84 years (mean [SD] age, 64.66 [10.79] years) were diagnosed with invasive lung ADC as their first cancer in the Taiwanese population of approximately 23 million. Table 1 reports the number of person-years and the number of patients with invasive lung ADC in this population from 2011 to 2019 by age, sex, stage (late or early), and smoking status, derived from eTables 5, 8A, and 8B in Supplement 1. Table 1 also includes similar information for SCC, SCLC, and lung cancer overall, derived from eTables 16A, 16B, 23A, and 23B in Supplement 1. Table 1 indicates that over 96.5 million person-years and 61 285 patients with invasive lung ADC were used for rate computation. Among them, an estimated 30 570 patients were female never smokers, 9893 were male never smokers, 2029 were female ever smokers, and 18 793 were male ever smokers. Among smokers, men had higher tobacco use by almost all examined metrics, including nearly twice the mean (SD) number of pack-years smoked (eg, 7.87 [8.30] for men aged 30-34 years vs 4.38 [5.27] for women aged 30-34 years) (eTables 12A and 12B in Supplement 1).

**Table 1.** Estimated Number of Person-Years and Patients With Invasive Lung Cancer Diagnosed at Age 40 to 84 Years From 2011 to 2019 in Taiwan by Age, Histologic Subtype, Sex, Smoking Status, and Stage

Age group, y, sex	Never smokers									Ever smokers								
	Person-years	Late stage, No.				Early stage, No.				Person-years	Late stage, No.				Early stage, No.			
		ADC	SCC	SCLC	LC	ADC	SCC	SCLC	LC		ADC	SCC	SCLC	LC	ADC	SCC	SCLC	LC
40-44																		
Female	7 623 184	521	25	7	599	456	NR^a^	NR^a^	490	608 196	90	7	5	120	34	0	0	39
Male	3 826 614	193	17	NR^a^	233	131	0	0	147	4 291 365	470	69	68	725	77	8	NR^a^	106
45-49																		
Female	7 581 740	1090	48	9	1242	745	NR^a^	NR^a^	818	518 334	152	10	17	199	52	0	NR^a^	60
Male	3 516 583	331	31	17	413	211	NR^a^	NR^a^	245	4 568 324	883	193	174	1450	195	21	0	244
50-54																		
Female	7 606 546	1728	88	27	2026	1305	7	NR^a^	1449	396 204	245	22	25	324	82	NR^a^	0	91
Male	3 214 355	505	47	27	642	343	NR^a^	0	382	4 768 566	1419	485	374	2633	370	38	5	467
55-59																		
Female	7 140 977	2534	121	33	2936	1648	NR^a^	NR^a^	1862	278 386	201	31	51	324	79	NR^a^	NR^a^	94
Male	3 262 602	779	101	41	1050	474	10	NR^a^	536	4 012 786	2190	756	617	4050	524	82	5	701
60-64																		
Female	5 978 970	3297	171	48	3838	1912	19	NR^a^	2141	207 693	240	35	71	374	73	7	0	92
Male	2 806 292	1094	154	80	1484	581	9	0	648	3 101 137	2453	1195	929	5146	646	138	17	939
65-69																		
Female	4 135 600	2991	164	46	3474	1639	15	NR^a^	1886	98 361	200	37	68	341	65	9	0	78
Male	1 844 112	1064	205	99	1542	510	22	0	602	2 038 297	2327	1360	909	5165	563	211	24	902
70-74																		
Female	2 965 352	2944	165	36	3462	1070	14	NR^a^	1225	72 775	157	45	76	300	40	9	0	54
Male	1 390 198	999	207	86	1504	408	29	NR^a^	487	1 204 971	2065	1542	967	5313	409	257	14	790
75-79																		
Female	2 407 370	2954	177	43	3658	860	11	5	1026	58 597	159	44	44	288	31	15	0	53
Male	1 007 605	994	270	125	1695	281	27	5	366	927 802	2016	1681	841	5374	341	227	24	685
80-84																		
Female	1 648 137	2474	131	55	3163	402	14	5	489	40 012	115	53	39	256	14	8	0	24
Male	746 166	851	259	138	1562	144	33	6	217	613 329	1631	1427	627	4541	214	179	25	493

Abbreviations: ADC, adenocarcinoma; LC, lung cancer; NR, not reported; SCC, squamous cell carcinoma; SCLC, small cell lung cancer.

^a^ Counts of more than 0 and fewer than 5 patients are not reported, consistent with a data protection rule of the Data Science Center, Ministry of Health and Welfare, Taiwan.

Based on eTables 10A, 10B, 18A, 18B, 25A, and 25B in Supplement 1, we report age-specific incidence rates and their 95% CIs by age, histologic subtype, sex, stage, smoking status, and period (2011-2015 or 2016-2019) in Table 2. Single-year incidence rates and other related information for lung ADC are presented in eTables 10A to 10C in Supplement 1. Table 2 shows that females had higher lung ADC rates than males by age, stage, and smoking status except for groups older than 75 years and compares incidence rates across age, sex, stage, smoking status, and periods. For example, for the age group 70 to 74 years in the period 2016-2019, the incidence of late-stage lung ADC for female never smokers was 99.53 (95% CI, 94.33-104.91) and for male never smokers was 65.73 (95% CI, 60.01-72.13).

**Table 2.** Age-Specific Incidence Rates of Lung Cancer by Age, Histologic Subtype, Period, Sex, Smoking Status, and Stage

Subtype, smoking status, age, y	Incidence, per 100 000 person-years (95% CI)			
	Females		Males	
	2011-2015	2016-2019	2011-2015	2016-2019
**ADC**				
Late stage				
Never smoker				
40-44	7.19 (6.38-8.03)	6.41 (5.50-7.30)	5.76 (4.68-6.81)	4.34 (3.44-5.24)
45-49	14.93 (13.67-16.09)	13.67 (12.42-14.98)	9.20 (7.78-10.51)	9.63 (8.11-11.08)
50-54	23.61 (22.15-25.04)	21.61 (20.06-23.19)	15.68 (13.85-17.57)	15.76 (13.64-17.79)
55-59	36.30 (34.57-38.38)	34.55 (32.71-36.58)	25.79 (23.42-28.28)	21.76 (19.30-24.25)
60-64	58.18 (55.34-60.82)	51.97 (49.44-54.69)	39.56 (36.25-43.15)	38.41 (35.17-41.53)
65-69	72.61 (68.46-76.45)	72.06 (68.41-75.49)	58.32 (53.18-63.51)	57.23 (52.55-61.70)
70-74	99.07 (93.76-104.05)	99.53 (94.33-104.91)	77.88 (71.45-83.93)	65.73 (60.01-72.13)
75-79	123.85 (118.15-129.57)	121.44 (114.47-127.89)	100.54 (92.13-109.38)	96.78 (88.19-104.89)
80-84	148.97 (140.56-156.86)	151.32 (143.00-159.96)	120.73 (109.98-131.62)	106.14 (95.57-117.06)
Ever smoker				
40-44	13.45 (9.56-17.33)	16.58 (12.06-21.56)	11.14 (9.87-12.53)	10.64 (9.13-12.20)
45-49	25.81 (19.97-31.67)	33.91 (26.33-41.69)	18.05 (16.45-19.65)	21.22 (19.02-23.34)
50-54	54.45 (44.70-64.35)	71.57 (59.74-84.67)	29.43 (27.21-31.36)	30.16 (27.76-32.59)
55-59	58.39 (46.03-69.66)	91.27 (75.67-109.66)	51.65 (48.66-54.63)	58.15 (54.56-61.72)
60-64	109.98 (91.12-129.25)	121.99 (99.88-143.77)	74.51 (70.71-78.64)	84.17 (79.68-88.84)
65-69	172.82 (134.08-207.92)	236.19 (191.91-277.21)	108.73 (102.04-114.86)	119.23 (112.66-125.74)
70-74	191.79 (151.07-228.83)	254.42 (198.12-320.59)	162.49 (153.06-171.64)	184.59 (171.81-196.82)
75-79	237.62 (187.62-285.76)	320.34 (250.51-396.64)	207.76 (195.79-219.23)	230.30 (215.89-245.08)
80-84	305.24 (234.82-375.72)	260.01 (186.87-343.60)	262.96 (245.99-278.98)	271.11 (251.80-292.97)
Early stage				
Never smoker				
40-44	3.71 (3.14-4.31)	8.74 (7.71-9.65)	2.25 (1.60-2.93)	4.56 (3.65-5.50)
45-49	7.50 (6.64-8.32)	12.82 (11.58-14.08)	5.11 (4.08-6.11)	7.00 (5.63-8.17)
50-54	12.23 (11.23-13.29)	23.32 (21.72-24.91)	7.74 (6.44-9.10)	14.26 (12.32-16.20)
55-59	16.19 (14.94-17.47)	31.08 (29.29-33.01)	11.66 (10.01-13.11)	17.66 (15.50-19.94)
60-64	25.11 (23.29-26.90)	39.13 (36.89-41.29)	16.49 (14.46-18.55)	24.81 (22.18-27.54)
65-69	29.34 (26.74-31.75)	48.20 (45.16-50.96)	18.97 (15.69-22.06)	34.10 (30.57-37.64)
70-74	30.05 (27.25-32.89)	43.50 (39.70-46.83)	24.28 (20.41-28.07)	34.54 (30.23-38.70)
75-79	29.61 (26.66-32.53)	42.45 (38.27-46.20)	24.55 (20.09-28.82)	31.03 (25.83-35.93)
80-84	19.21 (16.31-21.94)	29.92 (26.39-33.68)	19.78 (15.68-24.14)	18.72 (14.23-23.24)
Ever smoker				
40-44	2.81 (1.20-4.48)	9.02 (5.48-12.43)	1.36 (0.93-1.81)	2.43 (1.71-3.24)
45-49	6.02 (3.10-8.61)	14.97 (10.09-20.18)	3.08 (2.43-3.72)	6.03 (4.86-7.13)
50-54	14.74 (9.38-19.22)	28.46 (20.88-37.10)	6.06 (5.08-6.99)	9.94 (8.55-11.23)
55-59	17.96 (11.20-23.64)	42.81 (31.46-54.42)	9.74 (8.41-10.99)	17.13 (15.04-18.98)
60-64	25.86 (15.81-35.33)	44.91 (31.95-57.92)	17.26 (15.18-19.17)	24.75 (22.06-27.35)
65-69	60.72 (38.86-83.55)	72.45 (44.78-93.83)	22.50 (19.54-25.53)	32.37 (28.99-35.72)
70-74	49.63 (28.88-68.87)	62.89 (36.02-93.66)	28.20 (24.40-32.03)	42.49 (36.39-48.17)
75-79	19.26 (5.77-31.75)	101.41 (62.63-137.78)	30.38 (25.68-35.17)	45.52 (38.67-51.73)
80-84	34.54 (12.81-59.77)	33.37 (6.03-60.28)	28.81 (23.12-34.55)	45.14 (36.79-53.43)
**SCC**				
Late stage				
Never smoker				
40-44	0.45 (0.24-0.62)	0.19 (0.06-0.32)	0.43 (0.16-0.74)	0.45 (0.15-0.72)
45-49	0.67 (0.42-0.89)	0.58 (0.33-0.84)	1.00 (0.59-1.39)	0.75 (0.36-1.15)
50-54	1.13 (0.83-1.47)	1.19 (0.83-1.60)	1.35 (0.79-1.92)	1.59 (0.90-2.21)
55-59	1.79 (1.38-2.19)	1.57 (1.12-2.00)	3.53 (2.69-4.45)	2.60 (1.80-3.41)
60-64	2.85 (2.20-3.41)	2.88 (2.29-3.51)	5.59 (4.24-6.83)	5.39 (4.24-6.50)
65-69	4.48 (3.57-5.43)	3.52 (2.75-4.25)	12.62 (10.07-15.05)	10.03 (8.11-11.89)
70-74	6.16 (4.96-7.29)	4.81 (3.68-6.08)	17.83 (14.89-21.12)	11.86 (9.35-14.45)
75-79	7.50 (5.95-9.05)	7.15 (5.58-8.63)	32.55 (27.80-37.54)	21.25 (17.29-25.25)
80-84	8.13 (6.22-9.97)	7.71 (5.78-9.80)	34.82 (28.86-40.31)	34.37 (28.47-40.09)
Ever smoker				
40-44	0.66^a^	1.61^a^	1.63 (1.19-2.12)	1.55 (0.94-2.18)
45-49	1.13^a^	2.98 (0.44-5.25)	3.97 (3.20-4.75)	4.61 (3.57-5.57)
50-54	4.03 (1.79-7.15)	7.14 (3.48-11.02)	10.28 (9.04-11.47)	10.05 (8.74-11.42)
55-59	11.35 (6.22-16.17)	11.03 (5.10-16.15)	19.11 (17.14-20.80)	18.54 (16.76-20.48)
60-64	17.15 (9.30-25.11)	16.41 (8.99-23.97)	39.25 (36.13-42.45)	37.73 (34.48-40.99)
65-69	39.13 (23.32-56.35)	36.33 (19.19-53.31)	70.23 (65.11-75.79)	63.47 (58.84-68.41)
70-74	57.75 (35.55-75.54)	70.08 (39.62-100.86)	131.65 (122.83-140.16)	122.40 (112.27-132.52)
75-79	78.47 (46.18-109.69)	68.44 (37.58-96.03)	184.05 (172.15-195.60)	177.12 (163.90-191.30)
80-84	142.15 (93.93-196.40)	123.27 (72.34-174.81)	227.29 (212.48-241.83)	241.98 (222.04-262.74)
Early stage				
Never smoker				
40-64	0.14 (0.09-0.19)	0.04 (0.01-0.08)^b^	0.18 (0.09-0.27)	0.13 (0.05-0.21)
65-84	0.67 (0.46-0.87)	0.29 (0.16-0.45)	3.01 (2.31-3.69)	1.46 (1.00-1.88)
Ever smoker				
40-64	0.38^a^	0.83 (0.22-1.46)	1.33 (1.12-1.54)	1.45 (1.20-1.70)
65-84	12.27 (6.47-17.47)	17.94 (10.42-25.17)	18.60 (16.90-20.29)	17.87 (16.27-19.70)
**SCLC**				
Late stage				
Never smoker				
40-44	0.07^a^	0.12^a^	0.06^a^	0.05^a^
45-49	0.10^a^	0.14 (0.03-0.24)	0.52 (0.21-0.80)	0.44 (0.12-0.79)
50-54	0.31 (0.14-0.50)	0.41 (0.21-0.62)	0.88 (0.45-1.30)	0.82 (0.35-1.25)
55-59	0.47 (0.26-0.70)	0.45 (0.21-0.67)	1.22 (0.70-1.70)	1.31 (0.77-1.87)
60-64	0.75 (0.43-1.05)	0.86 (0.51-1.19)	2.86 (1.94-3.74)	2.87 (1.98-3.67)
65-69	1.14 (0.64-1.60)	1.07 (0.66-1.46)	5.10 (3.57-6.89)	5.58 (4.25-7.08)
70-74	1.10 (0.61-1.59)	1.38 (0.75-2.03)	5.68 (3.97-7.66)	6.63 (4.82-8.47)
75-79	1.64 (0.95-2.38)	2.00 (1.13-2.79)	14.79 (11.36-17.86)	10.05 (7.19-12.82)
80-84	3.72 (2.46-4.93)	2.93 (1.76-4.15)	22.72 (17.91-27.37)	13.36 (9.30-17.14)
Ever smoker				
40-44	1.20^a^	0.37^a^	1.19 (0.77-1.58)	2.18 (1.42-2.83)
45-49	3.34 (1.03-5.16)	3.16 (0.88-5.70)	3.66 (2.94-4.34)	4.04 (3.08-4.86)
50-54	4.47 (1.79-6.70)	8.71 (4.64-13.34)	7.34 (6.32-8.37)	8.51 (7.31-9.70)
55-59	17.28 (10.57-23.01)	19.74 (11.90-28.06)	14.29 (12.62-15.78)	16.68 (14.65-18.59)
60-64	29.75 (19.53-39.05)	38.76 (26.96-50.93)	31.03 (28.32-33.73)	28.78 (26.06-31.69)
65-69	65.43 (42.75-87.44)	74.09 (49.04-98.09)	41.97 (37.75-45.99)	47.03 (43.02-51.16)
70-74	88.09 (62.21-113.30)	132.08 (86.46-172.91)	75.90 (69.46-82.35)	86.72 (78.36-95.31)
75-79	72.74 (46.18-101.03)	78.93 (45.93-112.73)	87.27 (79.47-95.10)	95.19 (84.52-105.51)
80-84	101.48 (59.78-140.90)	93.84 (48.22-138.65)	94.25 (84.42-104.42)	115.77 (102.47-129.62)
Early stage				
Never smoker				
40-64	0.02^a^	0.02^a^	0.01^a^	0.01^a^
65-84	0.16 (0.05-0.28)	0.09 (0.02-0.18)	0.35 (0.13-0.59)	0.12^a^
Ever smoker				
40-64	0.09^a^	0.00^a^	0.09 (0.03-0.14)	0.20 (0.10-0.28)
65-84	0.00^a^	0.00^a^	1.88 (1.33-2.40)	1.75 (1.20-2.32)

Abbreviations: ADC, adenocarcinoma; SCC, squamous cell carcinoma; SCLC, small cell lung cancer.

^a^ The number of patients was less than 5.

^b^ The 95% CI for the exact binomial was used.

Table 3 reports age-specific female-to-male IRRs from 2011 to 2019 by smoking status and stage. For any given smoking status and stage combination, age-specific female-to-male IRRs for ADC were higher than 1, with nearly all 95% CIs higher than 1 (eg, for the age group 70-74 years, the female-to-male IRR for late-stage lung ADC was 1.38 [95% CI, 1.30-1.50] for never smokers and 1.26 [95% CI, 1.07-1.50] for ever smokers).

**Table 3.** Age-Specific IRRs for Females vs Males From 2011 to 2019 by Age, Histologic Subtype, Smoking Status, and Stage

Subtype, age, y	IRR (95% CI)			
	Late stage		Early stage	
	Never smoker	Ever smoker	Never smoker	Ever smoker
ADC				
40-44	1.36 (1.16-1.65)	1.36 (1.07-1.73)	1.75 (1.46-2.14)	3.14 (1.78-4.71)
45-49	1.53 (1.35-1.76)	1.52 (1.28-1.85)	1.64 (1.42-1.95)	2.33 (1.63-3.57)
50-54	1.45 (1.31-1.63)	2.08 (1.86-2.36)	1.61 (1.44-1.85)	2.67 (2.05-3.38)
55-59	1.49 (1.38-1.63)	1.32 (1.14-1.55)	1.59 (1.39-1.77)	2.18 (1.65-2.68)
60-64	1.41 (1.33-1.53)	1.46 (1.25-1.62)	1.55 (1.40-1.69)	1.68 (1.31-2.18)
65-69	1.25 (1.14-1.33)	1.78 (1.47-2.05)	1.43 (1.27-1.57)	2.40 (1.89-3.05)
70-74	1.38 (1.30-1.50)	1.26 (1.07-1.50)	1.23 (1.12-1.38)	1.61 (1.17-2.12)
75-79	1.24 (1.14-1.34)	1.25 (1.08-1.47)	1.28 (1.14-1.48)	1.44 (0.89-2.02)
80-84	1.32 (1.23-1.44)	1.08 (0.90-1.32)	1.26 (1.02-1.57)	0.98 (0.53-1.56)
SCC				
40-44	0.76 (0.39-1.79)	0.68 (0.32-1.48)	∞^a^	0.00^b^
45-49	0.71 (0.48-1.22)	0.46 (0.26-0.80)	0.96^c^	0.00^b^
50-54	0.79 (0.56-1.40)	0.54 (0.31-0.83)	0.65 (0.19-3.60)	0.70^c^
55-59	0.55 (0.42-0.71)	0.59 (0.40-0.81)	0.14^c^	0.36^c^
60-64	0.52 (0.42-0.65)	0.44 (0.29-0.57)	0.93 (0.43-2.32)	0.79 (0.26-1.33)
65-69	0.36 (0.28-0.46)	0.57 (0.42-0.75)	0.30 (0.18-0.58)	0.84 (0.41-1.56)
70-74	0.37 (0.31-0.47)	0.49 (0.34-0.62)	0.23 (0.12-0.54)	0.56 (0.23-1.07)
75-79	0.27 (0.22-0.34)	0.41 (0.29-0.56)	0.18 (0.07-0.40)	1.02 (0.62-1.71)
80-84	0.23 (0.16-0.27)	0.57 (0.40-0.77)	0.19 (0.10-0.40)	0.65 (0.32-1.27)
SCLC				
40-44	1.68^c^	0.52 (0.10-1.12)	∞^a^	0.00^b^
45-49	0.25 (0.07-0.46)	0.86 (0.48-1.44)	NA^d^	NA^d^
50-54	0.42 (0.25-0.91)	0.80 (0.53-1.26)	∞^a^	0.00^b^
55-59	0.36 (0.25-0.60)	1.19 (0.85-1.50)	0.46^c^	2.87^c^
60-64	0.28 (0.20-0.39)	1.14 (0.90-1.42)	∞^a^	0.00^b^
65-69	0.20 (0.15-0.30)	1.56 (1.15-2.06)	∞^a^	0.00^b^
70-74	0.20 (0.11-0.30)	1.31 (0.99-1.69)	1.28^c^	0.00^b^
75-79	0.15 (0.10-0.21)	0.84 (0.54-1.09)	0.49 (0.07-2.51)	0.00^b^
80-84	0.18 (0.12-0.26)	0.96 (0.66-1.32)	0.35 (0.08-1.13)	0.00^b^
Lung cancer				
40-44	1.29 (1.13-1.58)	1.17 (0.94-1.36)	1.68 (1.43-2.00)	2.62 (1.88-4.41)
45-49	1.39 (1.26-1.54)	1.21 (1.00-1.36)	1.55 (1.36-1.77)	2.19 (1.62-2.82)
50-54	1.33 (1.22-1.46)	1.48 (1.34-1.66)	1.60 (1.45-1.85)	2.35 (1.80-2.95)
55-59	1.28 (1.19-1.36)	1.15 (1.03-1.29)	1.59 (1.43-1.80)	1.92 (1.39-2.38)
60-64	1.21 (1.14-1.27)	1.09 (0.98-1.19)	1.55 (1.40-1.71)	1.46 (1.16-1.81)
65-69	1.00 (0.95-1.05)	1.37 (1.17-1.53)	1.40 (1.26-1.51)	1.80 (1.39-2.31)
70-74	1.08 (1.02-1.17)	0.94 (0.84-1.04)	1.18 (1.07-1.30)	1.13 (0.84-1.59)
75-79	0.90 (0.85-0.96)	0.85 (0.75-0.95)	1.17 (1.06-1.35)	1.22 (0.84-1.62)
80-84	0.92 (0.87-0.96)	0.87 (0.76-0.99)	1.02 (0.87-1.16)	0.75 (0.40-1.14)

Abbreviations: ADC, adenocarcinoma; IRR, incidence rate ratio; NA, not available; SCC; squamous cell carcinoma; SCLC, small cell lung cancer.

^a^ There were no males and 1 or more females.

^b^ There were no females and 1 or more males.

^c^ There were more than 0 females and males, but at least 1 of the categories had fewer than 5 patients.

^d^ The number of female patients was not available.

These results imply that never-smoking females had a higher rate of invasive lung ADC than their male counterparts for most combinations. Findings were similar for ever smokers because men had higher age-specific smoking exposure by nearly all examined metrics, including number of pack-years smoked, number of cigarettes smoked per day, number of years smoked, age at smoking initiation, and number of years since cessation. Only for the age groups 55 to 59 and 60 to 64 years had men experienced slightly longer times since cessation; details are provided in eTables 12A and 12B and the eMethods in Supplement 1.

Table 3 shows that almost all never- and ever-smoking females had lower rates of late-stage SCC and SCLC than their male counterparts. For early-stage SCC, never-smoking females older than 60 years also had lower rates than their male counterparts. No such statements can be made for early-stage SCLC because of small case numbers.

Table 4 reports age-specific IRRs comparing ever smokers with never smokers from 2011 to 2019 by histologic subtype, sex, and stage. The IRRs were higher than 1 for late-stage lung ADC for any given sex and age group combination, but they were not always higher than 1 for early-stage lung ADC among people younger than 60 years. For late-stage SCC and SCLC, these IRRs were higher than 1. For early-stage SCC, they were also higher than 1 among people aged 60 years or older. No similar statements can be made for early-stage SCLC because of small case numbers.

**Table 4.** Age-Specific IRRs for Ever Smokers vs Never Smokers From 2011 to 2019 by Age, Histologic Subtype, Sex, and Stage

Subtype, age, y	IRR (95% CI)			
	Late stage		Early stage	
	Female	Male	Female	Male
ADC				
40-44	2.17 (1.64-2.68)	2.17 (1.84-2.61)	0.94 (0.62-1.30)	0.52 (0.39-0.68)
45-49	2.04 (1.77-2.48)	2.06 (1.83-2.36)	1.01 (0.68-1.38)	0.71 (0.60-0.88)
50-54	2.72 (2.42-3.09)	1.89 (1.71-2.12)	1.21 (0.94-1.49)	0.73 (0.64-0.86)
55-59	2.04 (1.75-2.40)	2.29 (2.13-2.51)	1.23 (1.00-1.53)	0.90 (0.78-1.01)
60-64	2.10 (1.77-2.36)	2.03 (1.91-2.20)	1.10 (0.89-1.41)	1.01 (0.89-1.14)
65-69	2.81 (2.42-3.29)	1.98 (1.87-2.17)	1.67 (1.19-2.14)	1.00 (0.87-1.14)
70-74	2.17 (1.79-2.60)	2.38 (2.24-2.64)	1.52 (1.02-2.02)	1.16 (1.00-1.36)
75-79	2.21 (1.82-2.61)	2.20 (2.06-2.37)	1.48 (1.07-2.03)	1.32 (1.13-1.45)
80-84	1.91 (1.63-2.43)	2.33 (2.14-2.51)	1.40 (0.73-2.59)	1.81 (1.34-2.26)
SCC				
40-44	3.27 (0.90-8.16)	3.63 (2.47-6.50)	0.00^a^	∞^b^
45-49	3.06 (1.32-5.66)	4.78 (3.44-7.46)	0.00^a^	7.70^c^
50-54	4.73 (2.84-7.15)	6.98 (5.21-10.91)	6.40^c^	5.90^c^
55-59	6.64 (3.93-9.56)	6.10 (5.09-8.02)	17.10^c^	6.50 (3.66-14.38)
60-64	5.87 (3.78-8.36)	7.03 (5.84-8.37)	11.20 (2.58-24.11)	13.27 (7.36-32.67)
65-69	9.54 (6.75-14.16)	5.99 (5.23-6.96)	24.53 (9.63-65.97)	8.87 (5.45-14.56)
70-74	11.24 (7.78-15.51)	8.59 (7.45-10.19)	25.08 (11.73-66.77)	10.38 (7.38-17.28)
75-79	10.13 (7.13-13.63)	6.76 (6.03-7.63)	53.41 (25.06-133.77)	9.19 (6.24-13.51)
80-84	16.71 (11.72-24.79)	6.71 (5.81-7.58)	22.47 (7.78-78.87)	6.52 (4.64-10.16)
SCLC				
40-44	8.95 (2.32-45.40)	28.98 (12.17-71.34)	0.00^a^	∞^b^
45-49	27.43 (13.29-77.46)	7.94 (4.99-14.00)	NA^d^	0.00^a^
50-54	17.72 (10.49-31.85)	9.25 (6.62-16.67)	0.00^a^	∞^b^
55-59	39.72 (25.40-63.27)	12.15 (9.38-17.33)	25.65^c^	4.07^c^
60-64	42.53 (30.24-62.64)	10.46 (8.48-14.95)	0.00^a^	∞^b^
65-69	63.07 (41.05-81.60)	8.28 (6.88-10.56)	0.00^a^	∞^b^
70-74	85.57 (57.06-142.54)	13.01 (10.37-17.33)	0.00^a^	13.84^c^
75-79	42.26 (26.35-66.89)	7.32 (6.28-9.21)	0.00^a^	5.70 (1.82-25.29)
80-84	29.42 (18.12-44.02)	5.54 (4.51-6.88)	0.00^a^	5.11 (2.28-13.80)
Lung cancer				
40-44	2.52 (1.99-3.10)	2.78 (2.42-3.20)	1.00 (0.69-1.34)	0.64 (0.50-0.85)
45-49	2.34 (1.95-2.71)	2.70 (2.44-3.05)	1.08 (0.79-1.40)	0.77 (0.65-0.94)
50-54	3.07 (2.75-3.54)	2.76 (2.52-3.02)	1.21 (0.98-1.50)	0.82 (0.74-0.97)
55-59	2.83 (2.51-3.17)	3.14 (2.92-3.38)	1.29 (1.03-1.52)	1.06 (0.95-1.20)
60-64	2.81 (2.47-3.11)	3.14 (2.98-3.32)	1.24 (0.95-1.58)	1.31 (1.20-1.48)
65-69	4.12 (3.64-4.61)	3.03 (2.87-3.24)	1.75 (1.34-2.19)	1.36 (1.22-1.52)
70-74	3.54 (3.02-3.99)	4.07 (3.86-4.31)	1.80 (1.29-2.25)	1.87 (1.66-2.12)
75-79	3.23 (2.86-3.73)	3.44 (3.29-3.66)	2.11 (1.51-2.63)	2.03 (1.79-2.28)
80-84	3.34 (2.85-3.76)	3.54 (3.35-3.71)	2.03 (1.08-3.00)	2.76 (2.36-3.32)

Abbreviations: ADC, adenocarcinoma; IRR, incidence rate ratio; NA, not available; SCC; squamous cell carcinoma; SCLC, small cell lung cancer.

^a^ There were no ever-smoking patients and 1 or more never-smoking patients.

^b^ There were no never-smoking patients and 1 or more ever-smoking patients.

^c^ There were more than 0 ever-smoking and never-smoking patients, but at least 1 of the categories had fewer than 5 patients.

^d^ The number of both ever- and never-smoking patients was not available.

Table 5 reports age-specific IRRs for 2016 to 2019 vs 2011 to 2015 by histologic subtype, stage, smoking status, and sex. For late-stage lung ADC among never smokers, age- and sex-specific incidence rates were slightly lower from 2016 to 2019 than from 2011 to 2015; for late-stage lung ADC among ever smokers, the IRRs were slightly higher than 1 for most of the age groups. For early-stage lung ADC, both the lower and upper bounds of the 95% CIs for nearly all of the IRRs were higher than 1, indicating significantly higher incidence rates from 2016 to 2019 than from 2011 to 2015. For late-stage SCC and SCLC, there were few differences between the rates in these 2 periods. For early-stage SCC and SCLC, there seemed to be no support that the later period had higher incidence rates, mainly due to their small case numbers. The results for stages 3 and 4 ADC (eTables 10C and 11C in Supplement 1), SCC (eTables 18C and 19C in Supplement 1), SCLC (eTables 25C and 26C in Supplement 1), and lung cancer (eTables 32C and 33C in Supplement 1) were similar to the respective results for late-stage disease.

**Table 5.** Age-Specific IRRs From 2016 to 2019 vs 2011 to 2015 by Age, Histologic Subtype, Sex, Smoking Status, and Stage

Subtype, age, y	IRR (95% CI)			
	Never-smoking female	Never-smoking male	Ever-smoking female	Ever-smoking male
**ADC**				
Late stage				
40-44	0.89 (0.73-1.05)	0.75 (0.57-1.08)	1.23 (0.79-1.80)	0.95 (0.77-1.18)
45-49	0.92 (0.83-1.05)	1.05 (0.81-1.28)	1.31 (0.97-1.84)	1.18 (1.03-1.34)
50-54	0.92 (0.84-0.99)	1.01 (0.81-1.24)	1.31 (1.03-1.62)	1.02 (0.94-1.13)
55-59	0.95 (0.88-1.05)	0.84 (0.73-0.96)	1.56 (1.24-2.05)	1.13 (1.01-1.21)
60-64	0.89 (0.82-0.95)	0.97 (0.87-1.13)	1.11 (0.84-1.55)	1.13 (1.04-1.22)
65-69	0.99 (0.91-1.07)	0.98 (0.87-1.08)	1.37 (1.03-1.76)	1.10 (1.00-1.21)
70-74	1.00 (0.95-1.09)	0.84 (0.73-0.96)	1.33 (0.96-1.75)	1.14 (1.04-1.24)
75-79	0.98 (0.92-1.04)	0.96 (0.87-1.11)	1.35 (0.97-1.98)	1.11 (1.03-1.19)
80-84	1.02 (0.94-1.10)	0.88 (0.77-0.99)	0.85 (0.58-1.36)	1.03 (0.93-1.14)
Early stage				
40-44	2.36 (2.01-2.96)	2.02 (1.44-3.31)	3.21 (1.88-8.50)	1.78 (1.11-2.64)
45-49	1.71 (1.52-2.01)	1.37 (1.01-1.87)	2.49 (1.36-5.59)	1.96 (1.46-2.63)
50-54	1.91 (1.70-2.19)	1.84 (1.52-2.25)	1.93 (1.27-3.27)	1.64 (1.26-2.05)
55-59	1.92 (1.72-2.16)	1.51 (1.28-1.84)	2.38 (1.49-3.73)	1.76 (1.52-2.09)
60-64	1.56 (1.43-1.73)	1.50 (1.25-1.76)	1.74 (1.03-2.74)	1.43 (1.23-1.66)
65-69	1.64 (1.46-1.82)	1.80 (1.53-2.19)	1.19 (0.72-1.89)	1.44 (1.16-1.70)
70-74	1.45 (1.26-1.68)	1.42 (1.11-1.72)	1.27 (0.71-2.42)	1.51 (1.25-1.92)
75-79	1.43 (1.26-1.66)	1.26 (0.98-1.72)	5.27 (2.41-22.68)	1.50 (1.16-1.85)
80-84	1.56 (1.24-1.85)	0.95 (0.66-1.32)	0.97 (0.17-7.04)	1.57 (1.11-1.98)
**SCC**				
Late stage				
40-44	0.42 (0.12-1.15)	1.06 (0.35-5.26)	2.43^a^	0.95 (0.46-1.48)
45-49	0.87 (0.43-1.55)	0.75 (0.33-1.41)	2.64^a^	1.16 (0.88-1.51)
50-54	1.06 (0.67-1.68)	1.18 (0.63-2.05)	1.77 (0.69-4.49)	0.98 (0.81-1.15)
55-59	0.88 (0.61-1.22)	0.74 (0.46-1.18)	0.97 (0.51-2.39)	0.97 (0.82-1.16)
60-64	1.01 (0.73-1.37)	0.96 (0.69-1.26)	0.96 (0.48-1.83)	0.96 (0.85-1.07)
65-69	0.79 (0.58-1.10)	0.80 (0.63-1.01)	0.93 (0.49-1.87)	0.90 (0.81-1.01)
70-74	0.78 (0.57-1.07)	0.67 (0.50-0.83)	1.21 (0.71-2.40)	0.93 (0.83-1.04)
75-79	0.95 (0.67-1.29)	0.65 (0.52-0.88)	0.87 (0.38-1.59)	0.96 (0.86-1.07)
80-84	0.95 (0.66-1.37)	0.99 (0.77-1.31)	0.87 (0.50-1.39)	1.06 (0.96-1.17)
Early stage				
40-64	0.28 (0.08-0.58)	0.71 (0.22-1.58)	2.20^a^	1.09 (0.84-1.40)
65-84	0.43 (0.18-0.73)	0.49 (0.33-0.71)	1.46 (0.82-3.57)	0.96 (0.84-1.11)
**SCLC**				
Late stage				
40-44	1.61 (0.11-7.34)	0.93 (0.00-3.49)	0.31 (0.00-3.06)	1.83 (1.13-3.05)
45-49	1.43 (0.37-9.00)	0.85 (0.24-1.94)	0.95 (0.19-2.91)	1.10 (0.79-1.46)
50-54	1.34 (0.43-2.84)	0.93 (0.42-2.20)	1.95 (0.75-5.52)	1.16 (0.92-1.41)
55-59	0.94 (0.50-2.06)	1.07 (0.44-2.34)	1.14 (0.57-1.89)	1.17 (0.96-1.38)
60-64	1.15 (0.68-1.77)	1.01 (0.59-1.58)	1.30 (0.71-1.88)	0.93 (0.80-1.04)
65-69	0.94 (0.50-2.24)	1.09 (0.73-1.91)	1.13 (0.68-1.71)	1.12 (0.99-1.25)
70-74	1.25 (0.56-2.26)	1.17 (0.87-1.81)	1.50 (0.81-2.46)	1.14 (1.01-1.30)
75-79	1.22 (0.70-2.32)	0.68 (0.51-1.02)	1.09 (0.50-1.99)	1.09 (0.98-1.27)
80-84	0.79 (0.43-1.35)	0.59 (0.40-0.86)	0.92 (0.44-1.93)	1.23 (1.05-1.42)
Early stage				
40-64	1.16^a^	1.05^a^	0.00^b^	2.21 (1.13-6.33)
65-84	0.59 (0.18-2.47)	0.34^a^	NA^c^	0.93 (0.56-1.54)
**Lung cancer**				
Late stage				
40-44	0.91 (0.77-1.05)	0.81 (0.63-1.12)	1.29 (0.87-1.91)	0.98 (0.83-1.13)
45-49	0.93 (0.83-1.05)	1.05 (0.87-1.25)	1.32 (0.96-1.75)	1.14 (1.02-1.28)
50-54	0.93 (0.85-1.01)	0.97 (0.81-1.10)	1.29 (1.02-1.54)	1.03 (0.97-1.13)
55-59	0.96 (0.90-1.04)	0.87 (0.74-0.97)	1.40 (1.17-1.68)	1.11 (1.02-1.18)
60-64	0.93 (0.87-0.98)	1.01 (0.91-1.14)	1.14 (0.94-1.43)	1.05 (1.00-1.12)
65-69	0.99 (0.91-1.06)	0.95 (0.87-1.05)	1.30 (1.06-1.58)	1.02 (0.96-1.08)
70-74	1.01 (0.95-1.08)	0.82 (0.74-0.91)	1.31 (1.05-1.63)	1.04 (0.99-1.09)
75-79	0.95 (0.89-1.01)	0.84 (0.76-0.93)	1.10 (0.88-1.43)	1.02 (0.97-1.08)
80-84	0.98 (0.90-1.03)	0.87 (0.81-0.96)	0.78 (0.58-1.00)	1.06 (0.99-1.12)
Early stage				
40-44	2.30 (1.94-2.83)	2.01 (1.41-3.00)	2.62 (1.81-6.21)	1.81 (1.30-2.82)
45-49	1.76 (1.51-2.04)	1.40 (1.03-1.85)	2.61 (1.56-5.38)	1.79 (1.43-2.35)
50-54	1.98 (1.75-2.19)	1.94 (1.54-2.40)	2.24 (1.53-3.82)	1.54 (1.26-1.86)
55-59	2.08 (1.88-2.38)	1.51 (1.30-1.81)	2.35 (1.57-3.33)	1.72 (1.46-1.99)
60-64	1.68 (1.55-1.85)	1.56 (1.33-1.84)	1.89 (1.23-2.70)	1.38 (1.20-1.60)
65-69	1.81 (1.62-2.00)	1.89 (1.64-2.16)	1.53 (0.93-2.20)	1.33 (1.17-1.52)
70-74	1.54 (1.36-1.81)	1.33 (1.07-1.55)	1.51 (0.93-2.63)	1.42 (1.29-1.67)
75-79	1.52 (1.32-1.73)	1.17 (0.99-1.50)	3.28 (1.91-6.85)	1.28 (1.11-1.52)
80-84	1.53 (1.25-1.82)	0.92 (0.73-1.28)	0.86 (0.36-2.67)	1.31 (1.06-1.55)

Abbreviations: ADC, adenocarcinoma; IRR, incidence rate ratio; NA, not available; SCC; squamous cell carcinoma; SCLC, small cell lung cancer.

^a^ The number of patients in both periods was greater than 0, but at least 1 of the periods had fewer than 5 patients.

^b^ There were no patients from 2016 to 2019, and there was at least 1 patient from 2011 to 2015.

^c^ There were no patients from 2011 to 2019.

## Discussion

Based on a large Taiwanese population study with individual-level smoking information, we studied age-, sex-, stage-, and diagnosis year–specific incidence rates of lung ADC by smoking status from 2011 to 2019, a period in which LDCT lung cancer screening was not reimbursed by the National Health Insurance Program but was available commercially.^15,32,33^ We found that although smoking was generally more common among males, higher incidence rates of stage-specific lung ADC in females were observed among both never and ever smokers, and IRRs comparing ever smokers with never smokers were higher than 1 for late-stage lung ADC but not always for early-stage lung ADC among younger people.

According to Gao and colleagues,^15^ we may assume that LDCT lung cancer screening in Taiwan increased from the 2011 to 2015 period to the 2016 to 2019 period. This assumption is in line with the higher age- and sex-specific incidence rates that we found from 2016 to 2019 than from 2011 to 2015 for early-stage lung ADC. These rates may indicate overdiagnosis if we assume that the distributions of risk factors for ADC in Taiwan varied little with calendar year in this period. The observation that lung cancer detected by LDCT is mostly in an early stage^34,35^ and the finding that the rates of late-stage lung ADC from 2016 to 2019 were at most slightly larger than those from 2011 to 2015 jointly suggest that most late-stage lung ADCs were not detected by screening or incidentally.

Thus, the finding that females had higher incidence rates of late-stage lung ADC than males calls for other explanations, which might be attributed to a combination of genetic, hormonal, and societal factors. Because Taiwan’s National Health Insurance Program reduces the impact of accessibility, we first suggest the possibility of differential exposure to risk factors other than tobacco smoking (eg, home cooking fumes, environmental tobacco smoke^36^) and the potential effect modification of ADC risk factors by sex.^37^ We may also consider the sex differences in comorbidity before ADC diagnosis.^27^

In view of a US report that women had approximately twice as large an autopsy reservoir of indolent lesions of the lung as men^38^ (which nonetheless meet the criteria for lung cancer) and a Japanese report that never-smoking women had approximately twice as large an LDCT lung cancer detection rate as men,^39^ the current study provides support that women may be more susceptible to late-stage lung ADC. That some of the IRRs when comparing periods were higher than 1 for late-stage lung ADC among ever smokers deserves further study to see whether these findings were also due to the wide availability of LDCT lung cancer screening.

This study’s finding that some of the IRRs for ever vs never smokers for early-stage lung ADC were less than 1 among people younger than 60 years deserves attention. These findings might be an artifact of low accumulated tobacco exposure among younger people, the existence of risk factors other than tobacco smoking, and low incidence rates among younger people, among other factors.

Our finding that age-specific IRRs for late-stage lung ADC comparing ever smokers with never smokers were similar between sexes seems to be in line with findings of Freedman and colleagues^13^ and suggests that the relative risk of lung ADC among current smokers vs never smokers is similar in women and men. However, the IRRs for late-stage SCLC were larger among older females, which is an indication for more in-depth study.^13^

A recent report on the overdiagnosis of lung cancer associated with LDCT lung cancer screening in East Asia emphasizes that LDCT screening should be restricted to those at highest risk while rigorously maintaining adherence to growth assessment protocols.^19^ These suggestions are valid in general, but the overdiagnosis reported in the current study concerns particularly early-stage invasive lung ADC and prompts a comparison of the prognosis of early-stage ADC detected by screening with that detected by symptoms.^17^

The focus of this study was on lung ADC because of its large burden worldwide and its large number of cases in this study. For late-stage SCC and SCLC, this study found that males had higher rates than females irrespective of smoking status; because men had higher smoking exposure, the results regarding never smokers are more compelling. For early-stage SCC and SCLC, the case numbers were not large enough to draw definitive conclusions.

### Strengths and Limitations

The main strength of this study is that it was a large population study with the availability of individual-level smoking information for 92% of all patients diagnosed with lung ADC from 2011 to 2019 in Taiwan. The number of never-smoking patients with invasive lung ADC was larger than in other studies in the literature.^8,9,10,11^

A limitation of this study is its short study period. Although the excess early-stage incidence rates for lung ADC suggest some overdiagnosis, we may need a longer period to see if late-stage incidence rates decrease and if there is a sex difference in overdiagnosis. Another limitation resulted from the large error involved in the estimates of age-specific female smoking rates due to the low incidence of smoking among females, especially in older age groups.

## Conclusions

This cohort study found higher age- and stage-specific lung ADC incidence rates among women than men in Taiwan for both never and ever smokers, suggesting the possibility of differential exposures between sexes to risk factors other than smoking and potential modification of ADC risk factors by sex. Further work is needed to determine whether the sex differences in incidence of lung ADC replicate in other populations and other geographic regions.^40,41^ Given the large number of patients with invasive lung ADC among never smokers in this population and worldwide, there is urgency to discover the causes of this tumor and put preventive measures in place.

## Notes

Supplement 1.
eMethods.
**eTable 1A.** Age- and Sex-Specific Number of Cancer Survivors (Patients Diagnosed With Any Invasive Cancer) at the End of Each Year From 2010 to 2019, Based on the Linkage of TCR, TCOD, and NHIRD **eTable 1B.** Age- and Sex-Specific Number of Cancer Survivors at the End of Each Year From 2010 to 2019 in Taiwan, by Year of Diagnosis for Each Year From 1979 to 1988 **eTable 1C.** Age- and Sex-Specific Cancer Prevalence at Midyear of Each of the 9 Years From 2011 to 2019 **eTable 2.** Age- and Sex-Specific Midyear Population Size for 2011 to 2019 in Taiwan According to the Monthly Bulletin of Interior Statistics **eTable 3.** Age- and Sex-Specific Cancer-Free Midyear Population Size in Taiwan From 2011 to 2019 **eTable 4.** Age- and Sex-Specific Smoking Rate for Each Year From 2011 to 2019 **eTable 5.** Midyear Age-Specific Number of Cancer-Free Individuals by Sex and Smoking Status for Each Year From 2011 to 2019 **eTable 6.** Age- and Sex-Specific Number of Patients With Invasive Lung ADC Diagnosed for Each Year From 2011 to 2019 According to the TCR **eTable 7A.** Age-, Sex-, and Calendar Year–Specific Percentages of Patients With Late-Stage (Stage 2-4) ADC Among Patients With All Stages (1-4), Estimated Using the TCRLF for Each Year From 2011 to 2019 **eTable 7B.** Age-, Sex-, and Calendar Year–Specific Percentages of Ever-Smokers Among Corresponding Patients With Late-Stage (2-4) ADC in Taiwan **eTable 7C.** Age-, Sex-, and Year-Specific Percentages of Ever-Smokers Among Corresponding Patients With Early-Stage (1) ADC in Taiwan **eTable 7D.** Age-, Sex-, and Calendar Year–Specific Percentages of Patients With Stage 3-4 ADC Among Patients With All Stages (1-4) of Invasive ADC, Estimated Using the TCRLF for Each Year From 2011 to 2019 **eTable 7E.** Age-, Sex-, and Year-Specific Percentages of Ever-Smokers Among Corresponding Patients With Stage 3-4 ADC in Taiwan **eTable 8A.** Estimated Age- and Sex-Specific Number of Patients With Late-Stage Lung ADC in the TCR, by Smoking Status **eTable 8B.** Estimated Age- and Sex-Specific Number of Patients With Early-Stage Lung ADC in the TCR, by Smoking Status **eTable 8C.** Estimated Age- and Sex-Specific Number of Patients With Stage 3-4 Lung ADC in the TCR, by Smoking Status **eTable 9.** Number of Patients With Invasive Lung ADC in the TCR and TCRLF Having Known Smoking Status (Ever-Smoker or Never-Smoker), a Known Number of Pack-Years Smoked, and a Known Number of Years Since Quitting Smoking, 2011-2019 **eTable 10A.** Late-Stage ADC Age-Specific Incidence Rates for Each Single Year and for the Period 2011-2019, by Sex and Smoking Status **eTable 10B.** Early-Stage ADC Age-Specific Incidence Rates For Each Single Year and for the Period 2011-2019, by Sex and Smoking Status **eTable 10C.** Stage 3-4 ADC Age-Specific Incidence Rates for Each Single Year and for the Period 2011-2019, by Sex and Smoking Status **eTable 11A.** Age-Specific Late-Stage Lung ADC Incidence Rate Ratios and Their 95% CIs Comparing Sex, Smoking Status, and Periods **eTable 11B.** Age-Specific Early-Stage Lung ADC Incidence Rate Ratios and Their 95% CIs Comparing Sex, Smoking Status, and Periods **eTable 11C.** Age-Specific Late-Stage 3-4 Lung ADC Incidence Rate Ratios and Their 95% CIs Comparing Sex, Smoking Status, and Periods **eTable 12A.** Age-Specific Numbers of Never- and Ever-Smoking Males in the Taiwan Biobank **eTable 12B.** Age-Specific Numbers of Never- and Ever-Smoking Females in the Taiwan Biobank **eTable 13.** Histology Codes for Lung ADC That Appeared in the TCR for Each Year From 2011 to 2019 **eTable 14.** Age- and Sex-Specific Number of Patients With Invasive Lung SCC Diagnosed for Each Year From 2011 to 2019 According to the TCR **eTable 15A.** Age-, Sex-, and Calendar Year–Specific Percentages of Patients With Late-Stage (2-4) SCC Among Patients With All Stages (1-4) of Invasive SCC, Estimated Using the TCRLF for Each Year From 2011 to 2019 **eTable 15B.** Age-, Sex-, and Calendar Year–Specific Percentages of Ever-Smokers Among Corresponding Patients With Late-Stage (2-4) SCC in Taiwan **eTable 15C.** Age-, Sex-, and Calendar Year–Specific Percentages of Ever-Smokers Among Corresponding Patients With Early-Stage (1) SCC in Taiwan **eTable 15D.** Age-, Sex-, and Calendar Year–Specific Percentages of Patients With Stage 3-4 SCC Among Patients With All Stages (1-4) of Invasive SCC, Estimated Using the TCRLF for Each Year From 2011 to 2019 **eTable 15E.** Age-, Sex-, and Calendar Year–Specific Percentages of Ever-Smokers Among Corresponding Patients With Stage 3-4 SCC in Taiwan **eTable 16A.** Estimated Age- and Sex-Specific Number of Patients With Late-Stage Lung SCC in the TCR, by Smoking Status **eTable 16B.** Estimated Age- and Sex-Specific Number of Patients With Early-Stage Lung SCC in the TCR, by Smoking Status **eTable 16C.** Estimated Age- and Sex-Specific Number of Patients With Stage 3-4 Lung SCC in the TCR, by Smoking Status **eTable 17.** Number of Patients With Invasive Lung SCC in the TCR and TCRLF Having Known Smoking Status (Ever-Smoker or Never-Smoker), a Known Number of Pack-Years Smoked, and a Known Number of Years Since Quitting Smoking, 2011-2019 **eTable 18A.** Age-Specific Incidence Rates of Late-Stage SCC for the Period 2011-2019, by Sex and Smoking Status **eTable 18B.** Age-Specific Incidence Rates of Early-Stage SCC for the Period 2011-2019, by Sex and Smoking Status **eTable 18C.** Age-Specific Incidence Rates of Stage 3-4 SCC for the Period 2011-2019, by Sex and Smoking Status **eTable 19A.** Age-Specific Late-Stage Lung SCC Incidence Rate Ratios and Their 95% CIs Comparing Sex, Smoking Status, and Periods **eTable 19B.** Age-Specific Early-Stage Lung SCC Incidence Rate Ratios and Their 95% CIs Comparing Sex, Smoking Status, and Periods **eTable 19C.** Age-Specific Late-Stage 3-4 Lung SCC Incidence Rate Ratios and Their 95% CIs Comparing Sex, Smoking Status, and Periods **eTable 20.** Histology Codes for Lung SCC That Appeared in the TCR for Each Year From 2011 to 2019 **eTable 21.** Age- and Sex-Specific Number of Patients With Invasive SCLC Diagnosed From 2011 to 2019 According to the TCR **eTable 22A.** Age-, Sex-, and Calendar Year–Specific Percentages of Patients With Late-Stage (2-4) SCLC Among Patients With All Stages (1-4) of Invasive SCLC, Estimated Using the TCRLF for Each Year From 2011 to 2019 **eTable 22B.** Age-, Sex-, and Calendar Year–Specific Percentages of Ever-Smokers Among Corresponding Patients With Late-Stage (2-4) SCLC in Taiwan **eTable 22C.** Age-, Sex-, and Calendar Year–Specific Percentages of Ever-Smokers Among Corresponding Patients With Early-Stage (1) SCLC in Taiwan **eTable 22D.** Age-, Sex-, and Calendar Year–Specific Percentages of Patients With Stage 3-4 SCLC Among Patients With All Stages (1-4) of Invasive SCLC, Estimated Using the TCRLF for Each Year From 2011 to 2019 **eTable 22E.** Age-, Sex-, and Calendar Year–Specific Percentages of Ever-Smokers Among Corresponding Patients With Stage 3-4 SCLC in Taiwan **eTable 23A.** Estimated Age- and Sex-Specific Number of Patients With Late-Stage Lung SCLC in the TCR, by Smoking Status **eTable 23B.** Estimated Age- and Sex-Specific Number of Patients With Early-Stage Lung SCLC in the TCR, by Smoking Status **eTable 23C.** Estimated Age- and Sex-Specific Number of Patients With Stage 3-4 Lung SCLC in the TCR, by Smoking Status **eTable 24.** Number of Patients With Invasive Lung SCLC in the TCR and TCRLF Having Known Smoking Status (Ever-Smoker or Never-Smoker), a Known Number of Pack-Years Smoked, and a Known Number of Years Since Quitting Smoking, 2011-2019 **eTable 25A.** Late-Stage SCLC Age-Specific Incidence Rates for the Period 2011-2019, by Sex and Smoking Status **eTable 25B.** Early-Stage SCLC Age-Specific Incidence Rates for the Period 2011-2019, by Sex and Smoking Status **eTable 25C.** Stage 3-4 SCLC Age-Specific Incidence Rates for the Period 2011-2019, by Sex and Smoking Status **eTable 26A.** Age-Specific Late-Stage Lung SCLC Incidence Rate Ratios and Their 95% CIs Comparing Sex, Smoking Status, and Periods **eTable 26B.** Age-Specific Early-Stage Lung SCLC Incidence Rate Ratios and Their 95% CIs Comparing Sex, Smoking Status, and Periods **eTable 26C.** Age-Specific Late-Stage 3-4 Lung SCLC Incidence Rate Ratios and Their 95% CIs Comparing Sex, Smoking Status, and Periods **eTable 27.** Histology Codes for Lung SCLC That Appeared in the TCR for Each Year From 2011 to 2019 **eTable 28.** Age- and Sex-Specific Number of Patients With Invasive Lung Cancer Diagnosed for Each Year From 2011 to 2019 According to the TCR **eTable 29A.** Age-, Sex-, and Calendar Year–Specific Percentages of Patients With Late-Stage (2-4) Lung Cancer Among All Stages (1-4) of Invasive Cancer, Estimated Using the TCRLF for Each Year From 2011 to 2019 **eTable 29B.** Age-, Sex-, and Calendar Year–Specific Percentages of Ever-Smokers Among Corresponding Patients With Late-Stage (2-4) Lung Cancer in Taiwan **eTable 29C.** Age-, Sex-, and Calendar Year–Specific Percentages of Ever-Smokers Among Corresponding Patients With Early-Stage Lung Cancer in Taiwan **eTable 29D.** Age-, Sex-, and Calendar Year–Specific Percentages of Patients With Stage 3-4 Lung Cancer Among Patients With All Stages (1-4) of Invasive Lung Cancer, Estimated Using the TCRLF for Each Year From 2011 to 2019 **eTable 29E.** Age-, Sex-, and Calendar Year–Specific Percentages of Ever-Smokers Among Corresponding Patients With Stage 3-4 Lung Cancer in Taiwan **eTable 30A.** Estimated Age- and Sex-Specific Number of Patients With Late-Stage Lung Cancer in the TCR, by Smoking Status **eTable 30B.** Estimated Age- and Sex-Specific Number of Patients With Early-Stage Lung Cancer in the TCR, by Smoking Status **eTable 30C.** Estimated Age- and Sex-Specific Number of Patients With Stage 3-4 Lung Cancer in the TCR, by Smoking Status **eTable 31.** Number of Patients With Invasive Lung Cancer in the TCR and TCRLF Having Known Smoking Status (Ever-Smoker or Never-Smoker), a Known Number of Pack-Years Smoked, and a Known Number of Years Since Quitting Smoking, 2011-2019 **eTable 32A.** Age-Specific Incidence Rates of Late-Stage Lung Cancer for Each Single Year and for the Period 2011-2019, by Sex and Smoking Status **eTable 32B.** Age-Specific Incidence Rates of Early-Stage Lung Cancer for Each Single Year and for the Period 2011-2019, by Sex and Smoking Status **eTable 32C.** Age-Specific Incidence Rates of Stage 3-4 Lung Cancer for Each Single Year and for the Period 2011-2019, by Sex and Smoking Status **eTable 33A.** Age-Specific Late-Stage Lung Cancer Incidence Rate Ratios and Their 95% CIs Comparing Sex, Smoking Status, and Periods **eTable 33B.** Age-Specific Early-Stage Lung Cancer Incidence Rate Ratios and Their 95% CIs Comparing Sex, Smoking Status, and Periods **eTable 33C.** Age-Specific Late-Stage 3-4 Lung Cancer Incidence Rate Ratios and Their 95% CIs Comparing Sex, Smoking Status, and Periods
eReferences

Supplement 2.
Data Sharing Statement
