# Supplementary material for: Incidence of Lung Adenocarcinoma by Age, Sex, and Smoking Status in Taiwan
Source: JAMA Netw Open. 2023 Nov 1;6(11):e2340704. doi: 10.1001/jamanetworkopen.2023.40704 (PMC10620613; doi:10.1001/jamanetworkopen.2023.40704)
Supplement: Supplement 1. — eMethods. eTable 1A. Age- and Sex-Specific Number of Cancer Survivors (Patients Diagnosed With Any Invasive Cancer) at the End of Each Year From 2010 to 2019, Based on the Linkage of TCR, TCOD, and NHIRD eTable 1B. Age- and Sex-Specific Number of Cancer Survivors at the End of Each Year From 2010 to 2019 in Taiwan, by Year of Diagnosis for Each Year From 1979 to 1988 eTable 1C. Age- and Sex-Specific Cancer Prevalence at Midyear of Each of the 9 Years From 2011 to 2019 eTable 2. Age- and Sex-Specific Midyear Population Size for 2011 to 2019 in Taiwan According to the Monthly Bulletin of Interior Statistics eTable 3. Age- and Sex-Specific Cancer-Free Midyear Population Size in Taiwan From 2011 to 2019 eTable 4. Age- and Sex-Specific Smoking Rate for Each Year From 2011 to 2019 eTable 5. Midyear Age-Specific Number of Cancer-Free Individuals by Sex and Smoking Status for Each Year From 2011 to 2019 eTable 6. Age- and Sex-Specific Number of Patients With Invasive Lung ADC Diagnosed for Each Year From 2011 to 2019 According to the TCR eTable 7A. Age-, Sex-, and Calendar Year–Specific Percentages of Patients With Late-Stage (Stage 2-4) ADC Among Patients With All Stages (1-4), Estimated Using the TCRLF for Each Year From 2011 to 2019 eTable 7B. Age-, Sex-, and Calendar Year–Specific Percentages of Ever-Smokers Among Corresponding Patients With Late-Stage (2-4) ADC in Taiwan eTable 7C. Age-, Sex-, and Year-Specific Percentages of Ever-Smokers Among Corresponding Patients With Early-Stage (1) ADC in Taiwan eTable 7D. Age-, Sex-, and Calendar Year–Specific Percentages of Patients With Stage 3-4 ADC Among Patients With All Stages (1-4) of Invasive ADC, Estimated Using the TCRLF for Each Year From 2011 to 2019 eTable 7E. Age-, Sex-, and Year-Specific Percentages of Ever-Smokers Among Corresponding Patients With Stage 3-4 ADC in Taiwan eTable 8A. Estimated Age- and Sex-Specific Number of Patients With Late-Stage Lung ADC in the TCR, by Smoking Status eTable 8B. Estimated Age- a [file jamanetwopen-e2340704-s001.pdf]

Supplemental Online Content

Chien L, Jiang H, Tsai F, et al. Incidence of lung adenocarcinoma by age, sex, and smoking status in Taiwan. *JAMA Netw Open*. 2023;6(11):e2340704. doi:10.1001/jamanetworkopen.2023.40704

eMethods

eTable 1A. Age- and Sex-Specific Number of Cancer Survivors (Patients Diagnosed With Any Invasive Cancer) at the End of Each Year From 2010 to 2019, Based on the Linkage of TCR, TCOD, and NHIRD

eTable 1B. Age- and Sex-Specific Number of Cancer Survivors at the End of Each Year From 2010 to 2019 in Taiwan, by Year of Diagnosis for Each Year From 1979 to 1988

eTable 1C. Age- and Sex-Specific Cancer Prevalence at Midyear of Each of the 9 Years From 2011 to 2019

eTable 2. Age- and Sex-Specific Midyear Population Size for 2011 to 2019 in Taiwan According to the Monthly Bulletin of Interior Statistics

eTable 3. Age- and Sex-Specific Cancer-Free Midyear Population Size in Taiwan From 2011 to 2019

eTable 4. Age- and Sex-Specific Smoking Rate for Each Year From 2011 to 2019

eTable 5. Midyear Age-Specific Number of Cancer-Free Individuals by Sex and Smoking Status for Each Year From 2011 to 2019

eTable 6. Age- and Sex-Specific Number of Patients With Invasive Lung ADC Diagnosed for Each Year From 2011 to 2019 According to the TCR

eTable 7A. Age-, Sex-, and Calendar Year–Specific Percentages of Patients With Late-Stage (Stage 2-4) ADC Among Patients With All Stages (1-4), Estimated Using the TCRLF for Each Year From 2011 to 2019

eTable 7B. Age-, Sex-, and Calendar Year–Specific Percentages of Ever-Smokers Among Corresponding Patients With Late-Stage (2-4) ADC in Taiwan

© 2023 Chien et al. *JAMA Network Open.*

eTable 7C. Age-, Sex-, and Year-Specific Percentages of Ever-Smokers Among Corresponding Patients With Early-Stage (1) ADC in Taiwan

eTable 7D. Age-, Sex-, and Calendar Year–Specific Percentages of Patients With Stage 3-4 ADC Among Patients With all Stages (1-4) of Invasive ADC, Estimated Using the TCRLF for Each Year From 2011 to 2019

eTable 7E. Age-, Sex-, and Year-Specific Percentages of Ever-Smokers Among Corresponding Patients With Stage 3-4 ADC in Taiwan

eTable 8A. Estimated Age- and Sex-Specific Number of Patients With Late-Stage Lung ADC in the TCR, by Smoking Status

eTable 8B. Estimated Age- and Sex-Specific Number of Patients With Early-Stage Lung ADC in the TCR, by Smoking Status

eTable 8C. Estimated Age- and Sex-Specific Number of Patients With Stage 3-4 Lung ADC in the TCR, by Smoking Status

eTable 9. Number of Patients With Invasive Lung ADC in the TCR and TCRLF Having Known Smoking Status (Ever-Smoker or Never-Smoker), a Known Number of Pack-Years Smoked, and a Known Number of Years Since Quitting Smoking, 2011-2019

eTable 10A. Late-Stage ADC Age-Specific Incidence Rates for Each Single Year and for the Period 2011-2019, by Sex and Smoking Status

eTable 10B. Early-stage ADC Age-Specific Incidence Rates For Each Single Year and for the Period 2011-2019, by Sex and Smoking Status

eTable 10C. Stage 3-4 ADC Age-Specific Incidence Rates for Each Single Year and for the Period 2011-2019, by Sex and Smoking Status

eTable 11A. Age-Specific Late-Stage Lung ADC Incidence Rate Ratios and Their 95% CIs Comparing Sex, Smoking Status, and Periods

eTable 11B. Age-Specific Early-Stage Lung ADC Incidence Rate Ratios and Their 95% CIs Comparing Sex, Smoking Status, and Periods

eTable 11C. Age-Specific Late-Stage 3-4 Lung ADC Incidence Rate Ratios and their 95% CIs Comparing Sex, Smoking Status, and Periods

eTable 12A. Age-Specific Numbers of Never- and Ever-Smoking Males in the Taiwan Biobank

eTable 12B. Age-Specific Numbers of Never- and Ever-Smoking Females in the Taiwan Biobank

© 2023 Chien et al. *JAMA Network Open.*

eTable 13. Histology Codes for Lung ADC That Appeared in the TCR for Each Year From 2011 to 2019

eTable 14. Age- and Sex-Specific Number of Patients With Invasive Lung SCC Diagnosed for Each Year From 2011 to 2019 According to the TCR

eTable 15A. Age-, Sex-, and Calendar Year–Specific Percentages of Patients With Late-Stage (2-4) SCC Among Patients With All Stages (1-4) of Invasive SCC, Estimated Using the TCRLF for Each Year From 2011 to 2019

eTable 15B. Age-, Sex-, and Calendar Year–Specific Percentages of Ever-Smokers Among Corresponding Patients With Late-Stage (2-4) SCC in Taiwan

eTable 15C. Age-, Sex-, and Calendar Year–Specific Percentages of Ever-Smokers Among Corresponding Patients With Early-Stage (1) SCC in Taiwan

eTable 15D. Age-, Sex-, and Calendar Year–Specific Percentages of Patients With Stage 3-4 SCC Among Patients With all Stages (1-4) of Invasive SCC, Estimated Using the TCRLF for Each Year From 2011 to 2019

eTable 15E. Age-, Sex-, and Calendar Year–Specific Percentages of Ever-Smokers Among Corresponding Patients With Stage 3-4 SCC in Taiwan

eTable 16A. Estimated Age- and Sex-Specific Number of Patients With Late-Stage Lung SCC in the TCR, by Smoking Status

eTable 16B. Estimated Age- and Sex-Specific Number of Patients With Early-Stage Lung SCC in the TCR, by Smoking Status

eTable 16C. Estimated Age- and Sex-Specific Number of Patients With Stage 3-4 Lung SCC in the TCR, by Smoking Status

eTable 17. Number of Patients With Invasive Lung SCC in the TCR and TCRLF Having Known Smoking Status (Ever-Smoker or Never-Smoker), a Known Number of Pack-Years Smoked, and a Known Number of Years Since Quitting Smoking, 2011-2019

eTable 18A. Age-Specific Incidence Rates of Late-Stage SCC for the Period 2011-2019, by Sex and Smoking Status

eTable 18B. Age-Specific Incidence Rates of Early-Stage SCC for the Period 2011-2019, by Sex and Smoking Status

eTable 18C. Age-Specific Incidence Rates of Stage 3-4 SCC for the Period 2011-2019, by Sex and Smoking Status

eTable 19A. Age-Specific Late-Stage Lung SCC Incidence Rate Ratios and Their 95% CIs Comparing Sex, Smoking Status, and Periods

© 2023 Chien et al. *JAMA Network Open.*

eTable 19B. Age-Specific Early-Stage Lung SCC Incidence Rate Ratios and Their 95% CIs Comparing Sex, Smoking Status, and Periods

eTable 19C. Age-Specific Late-Stage 3-4 Lung SCC Incidence Rate Ratios and Their 95% CIs Comparing Sex, Smoking Status, and Periods

eTable 20. Histology Codes for Lung SCC That Appeared in the TCR for Each Year From 2011 to 2019

eTable 21. Age- and Sex-Specific Number of Patients With Invasive SCLC Diagnosed From 2011 to 2019 According to the TCR

eTable 22A. Age-, Sex-, and Calendar Year–Specific Percentages of Patients With Late-Stage (2-4) SCLC Among Patients With All Stages (1-4) of Invasive SCLC, Estimated Using the TCRLF for Each Year From 2011 to 2019

eTable 22B. Age-, Sex-, and Calendar Year–Specific Percentages of Ever-Smokers Among Corresponding Patients With Late-Stage (2-4) SCLC in Taiwan

eTable 22C. Age-, Sex-, and Calendar Year–Specific Percentages of Ever-Smokers Among Corresponding Patients With Early-Stage (1) SCLC in Taiwan

eTable 22D. Age-, Sex-, and Calendar Year–Specific Percentages of Patients With Stage 3-4 SCLC Among Patients With All Stages (1-4) of Invasive SCLC, Estimated Using the TCRLF for Each Year From 2011 to 2019

eTable 22E. Age-, Sex-, and Calendar Year–Specific Percentages of Ever-Smokers Among Corresponding Patients With Stage 3-4 SCLC in Taiwan

eTable 23A. Estimated Age- and Sex-Specific Number of Patients With Late-Stage Lung SCLC in the TCR, by Smoking Status

eTable 23B. Estimated Age- and Sex-Specific Number of Patients With Early-Stage Lung SCLC in the TCR, by Smoking Status

eTable 23C. Estimated Age- and Sex-Specific Number of Patients With Stage 3-4 Lung SCLC in the TCR, by Smoking Status

eTable 24. Number of Patients With Invasive Lung SCLC in the TCR and TCRLF Having Known Smoking Status (Ever-Smoker or Never-Smoker), a Known Number of Pack-Years Smoked, and a Known Number of Years Since Quitting Smoking, 2011-2019

eTable 25A. Late-Stage SCLC Age-Specific Incidence Rates for the Period 2011-2019, by Sex and Smoking Status

© 2023 Chien et al. *JAMA Network Open.*

eTable 25B. Early-Stage SCLC Age-Specific Incidence Rates for the Period 2011-2019, by Sex and Smoking Status

eTable 25C. Stage 3-4 SCLC Age-Specific Incidence Rates for the Period 2011-2019, by Sex and Smoking Status

eTable 26A. Age-Specific Late-Stage Lung SCLC Incidence Rate Ratios and Their 95% CIs Comparing Sex, Smoking Status, and Periods

eTable 26B. Age-Specific Early-Stage Lung SCLC Incidence Rate Ratios and Their 95% CIs Comparing Sex, Smoking Status, and Periods

eTable 26C. Age-Specific Late-Stage 3-4 Lung SCLC Incidence Rate Ratios and Their 95% CIs Comparing Sex, Smoking Status, and Periods

eTable 27. Histology Codes for Lung SCLC That Appeared in the TCR for Each Year From 2011 to 2019

eTable 28. Age- and Sex-Specific Number of Patients With Invasive Lung Cancer Diagnosed for Each Year From 2011 to 2019 According to the TCR

eTable 29A. Age-, Sex-, and Calendar Year–Specific Percentages of Patients With Late-Stage (2-4) Lung Cancer Among All Stages (1-4) of Invasive Cancer, Estimated Using the TCRLF for Each Year From 2011 to 2019

eTable 29B. Age-, Sex-, and Calendar Year–Specific Percentages of Ever-Smokers Among Corresponding Patients With Late-Stage (2-4) Lung Cancer in Taiwan

eTable 29C. Age-, Sex-, and Calendar Year–Specific Percentages of Ever-Smokers Among Corresponding Patients With Early-Stage Lung Cancer in Taiwan

eTable 29D. Age-, Sex-, and Calendar Year–Specific Percentages of Patients With Stage 3-4 Lung Cancer Among Patients With All Stages (1-4) of Invasive Lung Cancer, Estimated Using the TCRLF for Each Year From 2011 to 2019

eTable 29E. Age-, Sex-, and Calendar Year–Specific Percentages of Ever-Smokers Among Corresponding Patients With Stage 3-4 Lung Cancer in Taiwan

eTable 30A. Estimated Age- and Sex-Specific Number of Patients With Late-Stage Lung Cancer in the TCR, by Smoking Status

eTable 30B. Estimated Age- and Sex-Specific Number of Patients With Early-Stage Lung Cancer in the TCR, by Smoking Status

eTable 30C. Estimated Age- and Sex-Specific Number of Patients With Stage 3-4 Lung Cancer in the TCR, by Smoking Status

© 2023 Chien et al. *JAMA Network Open.*

eTable 31. Number of Patients With Invasive Lung Cancer in the TCR and TCRLF Having Known Smoking Status (Ever-Smoker or Never-Smoker), a Known Number of Pack-Years Smoked, and a Known Number of Years Since Quitting Smoking, 2011-2019

eTable 32A. Age-Specific Incidence Rates of Late-Stage Lung Cancer for Each Single Year and for the Period 2011-2019, by Sex and Smoking Status

eTable 32B. Age-Specific Incidence Rates of Early-Stage Lung Cancer for Each Single Year and for the Period 2011-2019, by Sex and Smoking Status

eTable 32C. Age-Specific Incidence Rates of Stage 3-4 Lung Cancer for Each Single Year and for the Period 2011-2019, by Sex and Smoking Status

eTable 33A. Age-Specific Late-Stage Lung Cancer Incidence Rate Ratios and Their 95% CIs Comparing Sex, Smoking Status, and Periods

eTable 33B. Age-Specific Early-Stage Lung Cancer Incidence Rate Ratios and Their 95% CIs Comparing Sex, Smoking Status, and Periods

eTable 33C. Age-Specific Late-Stage 3-4 Lung Cancer Incidence Rate Ratios and Their 95% CIs Comparing Sex, Smoking Status, and Periods

eReferences

This supplemental material has been provided by the authors to give readers additional information about their work.

© 2023 Chien et al. *JAMA Network Open.*

eMethods

Datasets used

The datasets and information used in this study of lung adenocarcinoma (ADC) included the Taiwan Cancer Registry (TCR), Taiwan Cause of Death Database (TCOD), National Health Insurance Research Database (NHIRD), Taiwan National Health Interview Survey (NHIS), Monthly Bulletin of the Interior Statistics (MBIS), and Taiwan Biobank.

Linkage of the TCR, TCOD, and NHIRD

The TCR has collected information on newly diagnosed primary cancer patients at all hospitals in Taiwan having 50+ beds since 1979. Its associated Taiwan Cancer Registry Long-Form (TCRLF) started in 2004. The quality of the TCR and TCRLF is high in terms of completeness, timeliness, and accuracy [1, 2]. The TCRLF has collected smoking information on lung cancer patients since 2011. This study considered only lung cancers that were the first invasive cancers in cancer patients.

The TCOD has included cause-of-death information for Taiwanese people since 1971. Its quality has been previously described [3]. During 1985—2020, there were 4,745,912 unique death records that included individuals' national identification card number (NICN), sex, birth date, death date, and cause of death. The TCOD adopted NICN in 1985. The original TCOD contains 4,763,623 records for this period; < 0.4% of the data were excluded during data cleaning.

The NHIRD is based on the administrative database of the National Health Insurance Program, which started in 1995 and has a coverage higher than 99% of its population of more than 23 million. Data in the NHIRD have proven to be valuable for health science research [4]. The linkage of the above datasets was made through NICN and was used in our own previous studies [5-7].

Cancer prevalence

Given a time point in the life of an individual, this individual is either cancer-free or a cancer survivor at this time point; it is cancer-free if this individual has never been diagnosed with any invasive cancer. This study used the linkage of the TCR during 1979—2019, TCOD during 1985—2020, and NHIRD during 2000—2020. Using these data, we estimated the cancer prevalence, which is the number of survivors of any invasive cancer, at the end of each year from 2010 to 2019. Cancer survivors were operationally defined as follows. For example, a 2012 cancer survivor was one included in the TCR during 1979—2012, not included in the TCOD during 1985—2012, and included in the NHIRD during 2000—2013. eTable 1A in the Supplement presents age- and sex-specific number of cancer survivors at the end of each year from 2010 to 2019.

© 2023 Chien et al. *JAMA Network Open.*

To understand the possible underestimation due to cancer survivors diagnosed before 1979, we present in eTable1B in the Supplement the age-, sex-, and calendar year-specific number of cancer survivors whose diagnoses were in the year between 1979 and 1988. Because of data protection regulation of the Data Science Center, Ministry of Health and Welfare, Taiwan, if the number in a cell is larger than 0 but less than 5, it is coded as <5; in this situation, we treated it as 2.5 for later uses. Although eTable 1B suggests that the underestimation in eTable 1A is unlikely serious, especially for males, we modified eTable 1A. For example, we modified each of the age- and sex-specific number of cancer survivors at the end of 2012, reported in eTable 1A, by adding three times the corresponding age- and sex-specific number of cancer survivors at the end of 2012 who were diagnosed in 1979. Considering the average of the estimated cancer prevalence of two consecutive years, we present in eTable 1C the age- and sex-specific cancer prevalence at midyear of each of the 9 years from 2011 to 2019.

Cancer-free population size by age, sex, and smoking status

Using the MBIS, eTable 2 in the Supplement reports midyear age- and sex-specific population sizes in Taiwan for each year during 2011—2019. Subtracting the age- and sex-specific cancer prevalence in eTable 1C from the corresponding population sizes in eTable 2, we report in eTable 3 the midyear age- and sex-specific cancer-free population size in Taiwan for each year from 2011 to 2019.

Using NHIS, we estimated and reported in eTable 4 in the Supplement age- and sex-specific smoking rate for each year during 2011—2019. NHIS is a collaborative project between Taiwan National Health Research Institutes (NHRI) and Health Promotion Administration (HPA) and has completed five surveys respectively in 2001, 2005, 2009, 2013, and 2017. NHIS data from 2001, 2005, 2009, and 2013 were used to study the time trend of cigarette smoking, cessation, and exposure to secondhand smoking in Taiwan [8], which also includes the survey design, response rates, and number of respondents for 2001, 2005, 2009, and 2013. The 2017 survey was conducted in the same manner as 2013 with a response rate 72.8% and 17,026 respondents. In this study, we first estimated age- and sex-specific smoking rate in Taiwan for the year 2009, 2013, and 2017, based on respectively NHIS survey data from 2009, 2013, and 2017; the estimation method was that reported in Chiang and Chang [8].

Because the female ever smoking rates were very low and hence their estimates involved larger errors, we performed a simple smoothing procedure. Given a survey year and the age-specific rates based on the survey described in the last paragraph, we replaced the rate of an age-group by the weighted average of the rates of three age groups: that for the group immediately younger, that for the group immediately older, and that for the group itself. Their weights were one quarter for those two neighboring ones and 1/2 for the group itself. Also, rates for 65 or older females were averaged. We considered these rates estimated based on the NHIS data. This procedure wasn't applied to males.

© 2023 Chien et al. *JAMA Network Open.*

Given an age group and sex combination and its estimated smoking rates in 2009, 2013, and 2017 described in the previous paragraph, we fitted a linear regression by least square method; the fitted values at 2011, 2012, 2013, …, 2019 were considered the smoking rates for that age group and sex combination and are reported in eTable 4 in the Supplement.

Using eTables 3 and 4, we obtained in eTable 5 in the Supplement the midyear age-specific number of cancer-free individuals by sex and smoking status for each year from 2011 to 2019.

Age- and sex-specific number of patients with ADC by smoking status

eTable 6 in the Supplement reports the age- and sex-specific number of invasive lung ADC patients diagnosed for each year from 2011 to 2019 according to the TCR. For each patient with invasive ADC reported in eTable 6, we obtained his/her stage information from the TCRLF. Their stages should never be 0, according to the coding rule of the TCR; we found more than 99.8% of them did have stage 1, 2, 3, or 4; we changed those with stage 0 into 1. Given an age, sex, and calendar year combination, eTable 7A reports the percentage of late-stage (stage 2—4) ADC patients among all stage (1—4) of invasive ADC patients specified by the combination.

Given an age, sex, and calendar year combination, eTable 7B reports the percentage of ever-smokers in the late-stage ADC patients, also estimated using the TCRLF. Using these, we report in eTable 8A in the Supplement the estimated age- and sex-specific number of late-stage lung ADC patients in the TCR by smoking status. To study early-stage (stage 1) ADC incidence, we present eTable 7C in the Supplement the early-stage counterpart of eTable 7B and eTable 8B the early-stage counterpart of eTable 8A. Note that for the period 2011—2019, 93.9% of the lung ADC patients in the TCRLF had smoking status information available; 98% of the invasive lung ADC patients in the TCR were included in the TCRLF (see eTable 9 in the Supplement).

Incidence rates and incidence rate ratios

Incidence rates are the ratios of estimated incident lung cancer cases to the estimated number of person-years. We considered age at diagnosis according to the 5-year age bands (40—44, …, 75—79, 80—84) and diagnosis periods 2011—2015, 2016—2019, and 2011—2019 in estimating the incidence rates and incidence rate ratios. Consider, for example, the female never-smoking late-stage lung ADC incidence rate for the age group 50—54 and the period 2011—2019. It is the fraction number whose numerator is the sum of the estimated number of never-smoking female late-stage ADC patients diagnosed at age 50—54 in 2011, that in 2012, …, that in 2019, and whose denominator is the sum of the estimated number of cancer-free female never-smokers whose age were 50—54 at the midyear of 2011, that at the midyear of 2012, …, and that at the midyear of 2019. The numerator is 1728 and denominator is

© 2023 Chien et al. *JAMA Network Open.*

7,606,546, shown in Table 1. They were obtained from eTable 8A and eTable 5, respectively. In fact, Table 1 reports the age- and sex-specific number of late-stage lung ADC patients diagnosed in the period by smoking status and the corresponding person-years for estimating the incidence rates.

eTable 10A in the Supplement reports the age- and sex-specific incidence rates for each single calendar year and for the period 2011—2019 for late-stage ADC patients by smoking status; it also included confidence intervals for those regarding the period 2011—2019.

We used the percentile bootstrap method to estimate the confidence intervals [9]. Associated with each fraction number in the last paragraph, we considered the population consisting of 0 s and 1 s in which the number of 1 s was equal to the numerator of the fraction and the number of the 1 s plus that of the 0 s was equal to the denominator. We considered 1,000 bootstrap samples for this population. Each bootstrap sample consisted of elements sampled with replacement from this population and had the same population size; the proportion of 1 s in the bootstrap sample resulted in the bootstrap sample incidence rate. The 2.5 and 97.5 percentiles of these bootstrap sample incidence rates gave the 95% bootstrap confidence interval of the incidence rate.

The corresponding results for early-stage ADC are in eTable 10B in the Supplement.

In fact, we considered other approaches to these confidence intervals and obtained similar results; we decided to use the bootstrap method because it is straightforward to obtain the confidence intervals for incidence rate ratios (IRRs).

The way we obtained the confidence intervals for IRRs is exemplified as follows. Consider the female-to-male IRR in the age group 50—54 and the period 2011—2019 as an example. For the fraction number and the associated population for the females described in the last three paragraphs, we also considered their counterparts for the males. We considered the population that was the union of the population associated to the females and that associated to the males. Its size was the sum of the two denominators and each individual in the population is either a male or a female and either a patient or a healthy person. We considered 1,000 bootstrap samples from this population. Each sample had the same size as the population and provided a disease incidence rate for each sex. The ratio of the female incidence rate to the male incidence rate was called the bootstrap sample female-to-male incidence rate ratio (IRR). The 2.5 and 97.5 percentiles of these 1000 bootstrap IRRs gave the 95% bootstrap confidence interval of female-to-male IRR. eTable 11A in the Supplement reports the estimates of various age-specific late-stage lung ADC incidence rate ratios and their 95% confidence intervals. Its counterparts for early-stage ADC are in eTable 11B and those for the stage 3 and 4 are in eTable 11C.

Sex difference in smoking experience

© 2023 Chien et al. *JAMA Network Open.*

We used the Taiwan Biobank dataset to report the sex difference in smoking experience for each age group. eTable 12 reports the age- and sex-specific number of ever- and never-smokers; among the ever-smokers, the estimated mean number of pack-years smoked, mean number of cigarettes smoked per day (intensity), mean number of years smoked (duration), mean age of smoking initiation; among the former smokers, the mean number of years since smoking quitting. Each of these variables indicates that male has higher smoke exposure than female except for smoking quit years. In fact, the mean number of smoking quit years for males were also shorter than those for females, except for the age groups 55—59 and 60—64. The Taiwan Biobank is an ongoing community-based cohort of Taiwanese participants aged 30—70 years who had no cancer diagnosis at enrollment. This study used all the Taiwan Biobank data provided to us by April 16, 2021, including a total of 132,720 participants. Additional information can be found in Feng and colleagues [10] and the Supplementary Materials of Chien and colleagues [11].

Histology codes

The TCR has information on the histology of lung cancer. Supplement eTable 13 lists the histology codes for ADC that appeared in the TCR for each year from 2011 to 2019.

Prevalence of lung squamous cell carcinoma and small cell lung cancer

The datasets and methods discussed for ADC could be used to study other histologic subtypes if there are enough patients in the datasets. Specifically, if the number of patients in an age, sex, stage, and smoking status combination is small, the estimated incidence rate for that combination may involve large error. More importantly, Data Science Center do not let us bring the number of patients of a combination out of the Center for subsequent analyses if that number is less than five and larger than 0, due to privacy protection consideration. Indeed, some of the combinations considered for ADC in the above cannot be studied for lung squamous cell carcinoma (SCC) and small cell lung cancer (SCLC). To circumvent these situations, we considered age groups 40—64 and 65—84 for early-stage SCC and SCLC and do not report confidence intervals when the number of patients is less than 5.

With these in mind, we prepared corresponding tables for SCC and SCLC. Specifically, eTables 14, 15, 16, 17, 18, 19, and 20 are the SCC counterparts of eTables 6, 7, 8, 9, 10, 11, and 13 for ADC respectively; eTables 21, 22, 23, 24, 25, 26, and 27 are the SCLC counterparts. We also prepared the corresponding eTables 28, 29, 30, 31, 32, and 33 for lung cancer, including all the subtypes.

List of abbreviations

ADC (lung adenocarcinoma)

© 2023 Chien et al. *JAMA Network Open.*

HPA (Health Promotion Administration)

IRR (incidence rate ratio)

MBIS (Monthly Bulletin of the Interior Statistics)

NHIRD (National Health Insurance Research Database)

NHIS (National Health Interview Survey)

NICN (national identification card number)

NHRI (National Health Research Institutes)

SCC (lung squamous cell carcinoma)

SCLC (small cell lung cancer)

TCR (Taiwan Cancer Registry)

TCRLF (Taiwan Cancer Registry Long-Form)

TCOD (Taiwan Cause of Death Database)

© 2023 Chien et al. *JAMA Network Open.*

eTable 1A. Age- and sex-specific number of cancer survivors (patients diagnosed with any invasive cancer) at the end of each year from 2010 to 2019, based on the linkage of TCR, TCOD, and NHIRD.

	2010		2011		2012		2013		2014		2015		2016		2017		2018		2019	
Age \ sex	M	F	M	F	M	F	M	F	M	F	M	F	M	F	M	F	M	F	M	F
35	827	1373	1030	1605	962	1556	1045	1569	1146	1732	1118	1838	1106	1795	1136	1799	1098	1714	1115	1811
36	943	1458	969	1583	1179	1857	1114	1788	1199	1850	1280	2025	1277	2070	1253	2062	1267	2107	1232	1990
37	1045	1605	1085	1669	1089	1863	1343	2126	1270	2099	1380	2176	1463	2340	1436	2413	1425	2425	1442	2442
38	1156	1883	1198	1847	1240	1947	1232	2144	1527	2406	1436	2456	1539	2506	1674	2679	1616	2758	1601	2859
39	1424	2208	1304	2162	1380	2134	1402	2272	1400	2507	1712	2792	1597	2783	1738	2907	1888	3112	1796	3153
40	1557	2492	1608	2520	1508	2485	1570	2462	1598	2652	1636	2899	1961	3201	1803	3149	1974	3325	2148	3593
41	1723	2768	1761	2862	1826	2900	1689	2871	1748	2850	1792	3049	1856	3268	2234	3700	2057	3619	2225	3826
42	1867	3048	1966	3209	2018	3238	2066	3268	1884	3275	2008	3268	1991	3482	2120	3775	2471	4249	2332	4181
43	1992	3229	2119	3470	2262	3619	2328	3677	2325	3704	2140	3811	2260	3729	2282	3987	2371	4275	2779	4880
44	2315	3931	2230	3685	2367	4001	2544	4094	2648	4193	2622	4255	2401	4322	2523	4241	2544	4525	2674	4870
45	2452	4285	2605	4417	2509	4236	2668	4586	2825	4650	2909	4772	2870	4892	2730	4960	2832	4881	2831	5187
46	2664	4689	2776	4885	2987	5015	2767	4791	2969	5171	3090	5286	3225	5512	3153	5506	3073	5616	3112	5552
47	3021	5298	3068	5289	3108	5478	3365	5641	3113	5403	3347	5808	3449	5934	3599	6184	3512	6291	3464	6331
48	3272	5501	3332	5887	3407	5924	3481	6115	3727	6274	3456	6031	3658	6453	3871	6647	4003	6921	3896	7044
49	3394	5912	3670	6176	3709	6539	3869	6564	3871	6786	4086	7001	3834	6673	4050	7271	4274	7381	4369	7739
50	3653	6294	3879	6564	4120	6863	4183	7289	4334	7235	4354	7461	4543	7744	4311	7413	4517	8071	4767	8191

© 2023 Chien et al. *JAMA Network Open.*

	2010		2011		2012		2013		2014		2015		2016		2017		2018		2019	
sex / Age	M	F	M	F	M	F	M	F	M	F	M	F	M	F	M	F	M	F	M	F
51	3954	6743	4084	7020	4349	7215	4631	7501	4707	8003	4859	7971	4794	8146	5066	8542	4801	8178	4939	8840
52	4051	6724	4395	7338	4611	7670	4801	7912	5055	8160	5194	8773	5370	8755	5222	8980	5521	9359	5270	8903
53	4292	6833	4541	7320	4931	8013	5051	8313	5344	8691	5581	8896	5669	9551	5851	9592	5698	9783	6048	10205
54	4681	7497	4766	7456	5051	7931	5422	8688	5687	9002	5827	9407	6116	9639	6169	10321	6363	10326	6236	10610
55	4870	7596	5192	8116	5288	8069	5617	8585	6017	9426	6251	9744	6409	10170	6717	10431	6782	11194	6938	11173
56	4951	7513	5368	8210	5704	8833	5831	8652	6222	9282	6626	10198	6779	10504	7046	10952	7298	11322	7439	12063
57	5014	7546	5492	8087	5962	8894	6247	9497	6377	9308	6819	10012	7189	10934	7407	11344	7660	11722	7925	12138
58	5307	7582	5502	8189	6072	8691	6529	9568	6892	10249	6910	9990	7344	10712	7846	11792	8055	12224	8267	12559
59	5757	8035	5893	8230	6102	8802	6658	9414	7148	10250	7478	10979	7477	10671	7976	11496	8547	12577	8660	13024
60	5253	6996	6394	8535	6495	8891	6692	9420	7234	10107	7862	10969	8141	11725	8129	11493	8664	12323	9314	13448
61	5151	6670	5772	7544	7017	9207	7081	9521	7308	10117	7906	10810	8464	11661	8823	12626	8835	12266	9322	13158
62	4840	6120	5654	7159	6297	8142	7652	9850	7664	10199	7914	10797	8571	11511	9160	12453	9659	13373	9552	13121
63	4559	5603	5286	6521	6212	7724	6895	8709	8345	10509	8369	10834	8553	11493	9258	12240	9935	13279	10385	14167
64	3941	4480	4971	5974	5832	6968	6770	8264	7413	9301	9003	11235	8982	11516	9167	12195	9998	13019	10715	14100
65	3639	4283	4272	4815	5393	6361	6380	7465	7267	8772	7935	9927	9682	11976	9704	12297	9990	12934	10848	13767
66	4473	5108	3932	4604	4648	5127	5848	6796	6981	7978	7824	9255	8545	10429	10370	12750	10476	13051	10791	13661
67	4838	5441	4855	5450	4245	4914	5033	5488	6303	7144	7458	8396	8387	9774	9157	11101	11192	13415	11176	13807
68	4970	5580	5251	5762	5255	5806	4545	5186	5335	5845	6731	7484	7968	8806	9105	10412	9790	11734	11912	14094
69	5300	5588	5330	5855	5622	6080	5665	6129	4903	5475	5710	6164	7173	7886	8438	9252	9737	10970	10481	12288

© 2023 Chien et al. *JAMA Network Open.*

	2010		2011		2012		2013		2014		2015		2016		2017		2018		2019	
sex / Age	M	F	M	F	M	F	M	F	M	F	M	F	M	F	M	F	M	F	M	F
70	5254	5851	5669	5893	5682	6211	6059	6397	5955	6413	5236	5759	6048	6439	7636	8235	8927	9690	10358	11487
71	5226	5473	5627	6122	6027	6190	6138	6499	6440	6700	6318	6682	5537	5956	6357	6683	8040	8576	9419	10083
72	5095	5405	5565	5735	6045	6340	6377	6524	6466	6759	6707	6973	6613	6969	5798	6206	6704	6947	8511	8968
73	5097	5213	5320	5587	5911	5987	6434	6677	6691	6853	6813	7008	7021	7255	6937	7268	6119	6438	7020	7238
74	5055	4945	5415	5413	5638	5818	6243	6240	6753	6940	7055	7089	7090	7234	7358	7541	7311	7550	6390	6691
75	5078	4999	5283	5132	5663	5653	5944	6043	6640	6505	6989	7174	7271	7320	7287	7509	7671	7792	7619	7802
76	5102	4774	5269	5153	5493	5270	5830	5868	6152	6293	6833	6703	7230	7310	7483	7607	7497	7697	7856	8013
77	5116	4446	5200	4915	5442	5311	5673	5427	6056	5998	6288	6444	6856	6792	7413	7503	7662	7750	7677	7841
78	5243	4030	5249	4531	5408	4989	5557	5397	5761	5518	6203	6162	6353	6597	6958	6926	7514	7722	7808	7889
79	5364	4083	5318	4140	5276	4557	5454	5092	5616	5487	5854	5617	6184	6259	6324	6646	7058	6979	7593	7806
80	5569	3822	5400	4107	5314	4185	5286	4637	5438	5083	5619	5476	5787	5589	6178	6305	6348	6630	7073	7057
81	5232	3528	5584	3783	5373	4140	5240	4189	5230	4636	5389	5086	5557	5453	5713	5643	6110	6222	6318	6646
82	4876	3296	5200	3565	5533	3780	5334	4085	5132	4180	5065	4624	5219	5078	5425	5392	5603	5657	5938	6217
83	4379	2940	4842	3215	5041	3541	5388	3755	5186	4035	5061	4162	4864	4533	5114	4939	5322	5363	5449	5518
84	4042	2690	4232	2863	4716	3171	4863	3437	5192	3683	5013	3960	4800	4029	4741	4426	4967	4790	5138	5230

© 2023 Chien et al. *JAMA Network Open.*

eTable 1B. Age- and sex-specific number of cancer survivors at the end of each year from 2010 to 2019 in Taiwan by year of diagnosis for each year from 1979 to 1988.

Criterion	diag_y / SEX	Age	1979	1980	1981	1982	1983	1984	1985	1986	1987	1988
2010	Male	30-39	0	0	<5	<5	20	15	20	18	34	37
		40-49	<5	<5	<5	5	22	43	42	49	70	88
		50-59	5	8	17	14	68	78	91	115	160	176
		60-69	6	16	16	18	86	91	107	141	158	193
		70-79	18	16	20	17	105	121	147	178	221	218
		80-89	5	16	28	19	123	122	120	183	232	241
	Female	30-39	<5	<5	5	5	10	12	15	15	44	34
		40-49	<5	8	5	7	42	53	67	72	150	146
		50-59	17	23	33	31	124	189	207	279	372	415
		60-69	25	35	32	66	215	286	343	383	526	577
		70-79	36	60	62	76	358	374	435	465	581	591
		80-89	20	30	35	44	171	190	209	240	314	289
2011	Male	30-39	0	0	<5	<5	18	16	20	22	31	35
		40-49	0	<5	<5	6	25	39	39	43	57	81
		50-59	5	6	15	11	59	68	83	108	153	164
		60-69	5	12	15	18	92	97	103	134	156	177
		70-79	14	18	15	13	84	101	131	163	208	211
		80-89	7	14	27	19	125	123	127	167	207	225
	Female	30-39	<5	<5	<5	5	9	11	13	13	34	29
		40-49	<5	8	5	5	38	40	58	62	143	126
		50-59	12	20	25	28	113	172	193	238	315	367
		60-69	27	30	32	60	196	279	316	391	519	562
		70-79	35	67	63	69	348	358	438	443	557	603
		80-89	21	24	35	48	180	211	220	259	329	300
2012	Male	30-39	0	0	<5	<5	22	22	20	25	32	30
		40-49	0	<5	<5	6	22	32	38	39	54	81
		50-59	5	5	11	9	51	63	75	98	144	143
		60-69	<5	12	17	16	86	92	97	126	151	178
		70-79	15	17	12	14	81	94	118	139	202	184
		80-89	7	12	28	17	120	114	121	160	192	212
	Female	30-39	<5	0	<5	5	8	10	13	11	30	27

© 2023 Chien et al. *JAMA Network Open.*

Criterion	diag_y SEX	Age	1979	1980	1981	1982	1983	1984	1985	1986	1987	1988
		40-49	<5	6	5	<5	34	31	52	56	127	118
		50-59	11	17	21	24	101	155	167	210	293	326
		60-69	25	30	31	57	184	256	299	370	496	546
		70-79	31	63	60	60	330	359	431	441	533	593
		80-89	21	27	36	56	187	213	228	263	346	299
2013	Male	30-39	0	0	<5	<5	21	29	24	26	32	31
		40-49	0	<5	<5	5	19	30	35	30	51	78
		50-59	5	<5	12	8	49	57	69	99	128	133
		60-69	<5	11	16	16	81	83	91	115	156	172
		70-79	12	15	9	14	71	92	114	132	169	173
		80-89	6	11	27	15	109	101	100	150	200	193
	Female	30-39	<5	0	<5	<5	8	11	14	15	30	30
		40-49	<5	5	<5	<5	25	25	45	49	107	97
		50-59	11	15	20	21	91	134	160	189	272	297
		60-69	20	33	32	54	183	246	279	355	466	527
		70-79	35	55	50	63	309	338	420	423	533	583
		80-89	23	32	40	51	187	234	228	266	349	315
2014	Male	30-39	0	0	<5	<5	18	26	27	28	33	32
		40-49	0	<5	<5	<5	19	23	29	24	45	67
		50-59	<5	<5	12	7	36	59	53	91	123	124
		60-69	<5	11	11	14	82	80	98	108	145	167
		70-79	12	10	11	15	70	86	110	128	159	165
		80-89	5	11	21	12	99	89	89	134	178	183
	Female	30-39	<5	0	<5	<5	8	12	12	17	29	30
		40-49	<5	5	<5	<5	19	23	40	45	90	83
		50-59	9	13	17	17	90	114	150	171	252	259
		60-69	20	31	29	48	163	237	252	331	451	512
		70-79	33	51	47	59	288	318	397	423	526	565
		80-89	22	31	38	54	193	236	242	268	336	325
2015	Male	30-39	0	0	<5	<5	15	23	27	34	34	32
		40-49	0	<5	<5	<5	18	21	26	22	37	60
		50-59	<5	<5	11	7	31	54	46	80	115	114
		60-69	<5	10	10	13	83	76	94	100	137	168
		70-79	11	9	11	15	60	84	98	123	147	150
		80-89	5	8	18	12	86	84	90	125	172	173

© 2023 Chien et al. *JAMA Network Open.*

Criterion	diag_y / SEX	Age	1979	1980	1981	1982	1983	1984	1985	1986	1987	1988
	Female	30-39	<5	0	<5	<5	7	12	11	18	26	28
		40-49	<5	<5	<5	<5	17	16	35	34	75	74
		50-59	10	12	13	16	74	96	133	151	244	231
		60-69	19	30	27	45	167	236	248	332	432	498
		70-79	28	44	36	57	261	306	369	399	487	547
		80-89	22	33	40	50	198	234	248	272	343	318
2016	Male	30-39	0	0	<5	<5	12	23	24	33	30	36
		40-49	0	<5	<5	<5	20	17	24	19	36	50
		50-59	<5	<5	9	7	28	52	43	72	100	111
		60-69	<5	7	10	13	79	72	86	96	146	156
		70-79	10	11	9	13	57	80	87	114	138	142
		80-89	6	6	13	12	77	74	87	120	156	164
	Female	30-39	<5	0	<5	<5	6	10	11	17	23	31
		40-49	<5	<5	<5	<5	13	13	32	28	69	63
		50-59	9	10	11	13	64	81	108	131	219	203
		60-69	19	29	27	40	161	230	252	327	418	477
		70-79	24	42	33	60	241	292	352	370	470	534
		80-89	20	30	38	48	196	222	243	268	344	314
2017	Male	30-39	0	0	<5	<5	12	23	23	31	29	40
		40-49	0	0	<5	<5	16	14	21	17	35	40
		50-59	<5	<5	8	6	27	46	42	62	92	105
		60-69	<5	6	11	14	75	73	79	96	137	157
		70-79	7	12	9	9	55	75	83	116	127	138
		80-89	7	5	11	12	71	69	84	104	146	147
	Female	30-39	<5	0	0	<5	6	8	9	17	22	28
		40-49	<5	<5	5	<5	12	13	28	23	59	57
		50-59	8	11	8	11	56	72	97	104	194	177
		60-69	17	27	29	37	148	210	232	311	401	460
		70-79	27	43	30	54	222	270	334	359	457	525
		80-89	17	22	32	51	207	219	248	258	340	324
2018	Male	30-39	0	0	<5	<5	12	21	22	29	25	39
		40-49	0	0	<5	<5	14	12	19	15	35	36
		50-59	<5	<5	6	7	25	43	40	54	78	99
		60-69	<5	6	12	11	63	66	72	95	141	147
		70-79	6	9	7	9	57	64	83	108	120	127

© 2023 Chien et al. *JAMA Network Open.*

Criterion	diag_y / SEX	Age	1979	1980	1981	1982	1983	1984	1985	1986	1987	1988
		80-89	7	<5	10	9	65	74	72	100	136	146
	Female	30-39	<5	0	0	<5	5	6	7	14	22	27
		40-49	<5	<5	<5	<5	10	11	22	24	54	47
		50-59	<5	9	9	6	53	68	82	89	173	167
		60-69	18	24	27	36	138	188	220	287	387	431
		70-79	25	37	30	51	190	267	312	362	436	505
		80-89	15	29	29	52	215	216	257	240	340	332
2019	Male	30-39	0	0	<5	<5	9	18	19	29	24	37
		40-49	0	0	<5	<5	15	14	15	13	34	34
		50-59	0	<5	<5	6	22	36	39	47	64	86
		60-69	<5	5	11	9	57	68	73	86	138	140
		70-79	<5	10	9	8	60	61	74	104	114	131
		80-89	7	<5	9	8	61	64	72	89	125	124
	Female	30-39	0	0	0	<5	<5	<5	6	11	19	27
		40-49	<5	<5	<5	<5	9	14	17	20	46	38
		50-59	<5	8	6	6	47	58	73	79	158	149
		60-69	18	21	26	29	122	180	188	267	347	417
		70-79	20	34	25	53	186	242	303	331	434	476
		80-89	15	32	29	46	213	218	264	265	337	343

© 2023 Chien et al. *JAMA Network Open.*

eTable 1C. Age- and sex-specific cancer prevalence at midyear of each of the 9 years from 2011 to 2019.

	2011		2012		2013		2014		2015		2016		2017		2018		2019	
sex / Age	M	F	M	F	M	F	M	F	M	F	M	F	M	F	M	F	M	F
35	928.50	1489.75	996.00	1581.25	1003.50	1563.25	1095.50	1651.25	1132.00	1785.75	1112.00	1817.25	1121.00	1797.75	1117.00	1757.25	1106.50	1762.88
36	956.00	1521.25	1074.00	1720.75	1146.50	1823.25	1156.50	1819.75	1239.50	1938.25	1278.50	2048.25	1265.00	2066.75	1260.00	2085.25	1249.50	2048.88
37	1065.00	1637.75	1087.00	1766.75	1216.00	1995.25	1306.50	2113.25	1325.00	2138.25	1421.50	2258.75	1449.50	2377.25	1430.50	2419.75	1433.50	2433.88
38	1177.00	1865.75	1219.00	1897.75	1236.00	2046.25	1379.50	2275.75	1481.50	2431.75	1487.50	2481.75	1606.50	2593.25	1645.00	2719.25	1608.50	2808.88
39	1364.00	2185.75	1342.00	2148.75	1391.00	2203.75	1401.00	2390.25	1556.00	2650.25	1654.50	2788.25	1667.50	2845.75	1813.00	3010.25	1842.00	3132.88
40	1582.88	2506.75	1558.00	2503.25	1539.00	2474.25	1584.00	2557.75	1617.00	2776.25	1798.50	3050.75	1882.00	3175.75	1888.50	3237.75	2061.00	3459.75
41	1742.38	2815.75	1793.50	2881.75	1757.50	2886.25	1718.50	2861.25	1770.00	2950.25	1824.00	3159.25	2045.00	3484.75	2145.50	3660.25	2141.00	3723.25
42	1916.88	3129.25	1992.00	3224.25	2042.00	3253.75	1975.00	3272.25	1946.00	3272.25	1999.50	3375.75	2055.50	3629.25	2295.50	4012.75	2401.50	4215.75
43	2055.88	3350.25	2190.50	3545.25	2295.00	3648.75	2326.50	3691.25	2232.50	3758.25	2200.00	3770.75	2271.00	3858.75	2326.50	4131.75	2575.00	4578.25
44	2272.88	3808.75	2298.50	3843.75	2455.50	4048.25	2596.00	4144.25	2635.00	4224.75	2511.50	4289.25	2462.00	4282.25	2533.50	4383.75	2609.00	4698.25
45	2528.88	4351.75	2557.00	4327.25	2588.50	4411.75	2746.50	4618.75	2867.00	4711.75	2889.50	4832.75	2800.00	4926.75	2781.00	4921.25	2831.50	5034.75
46	2720.38	4787.75	2881.50	4950.75	2877.00	4903.75	2868.00	4981.75	3029.50	5229.25	3157.50	5399.75	3189.00	5509.75	3113.00	5561.75	3092.50	5584.75
47	3044.88	5294.25	3088.00	5384.25	3236.50	5560.25	3239.00	5522.75	3230.00	5606.25	3398.00	5871.75	3524.00	6059.75	3555.50	6238.25	3488.00	6311.75
48	3302.38	5694.75	3369.50	5906.25	3444.00	6020.25	3604.00	6195.25	3591.50	6153.25	3557.00	6242.75	3764.50	6550.75	3937.00	6784.75	3949.50	6983.25
49	3532.38	6044.75	3689.50	6358.25	3789.00	6552.25	3870.00	6675.75	3978.50	6894.25	3960.00	6837.75	3942.00	6972.75	4162.00	7326.75	4321.50	7560.75
50	3767.50	6433.35	4001.00	6716.95	4153.00	7079.30	4259.63	7265.00	4344.75	7350.85	4449.25	7605.35	4427.75	7581.05	4414.75	7743.58	4642.38	8131.75
51	4020.50	6885.85	4218.00	7120.95	4491.50	7361.30	4670.13	7755.00	4783.75	7989.85	4827.25	8061.35	4930.75	8346.55	4934.25	8361.58	4870.38	8509.75

© 2023 Chien et al. *JAMA Network Open.*

	2011		2012		2013		2014		2015		2016		2017		2018		2019	
sex / Age	M	F	M	F	M	F	M	F	M	F	M	F	M	F	M	F	M	F
52	4224.50	7035.35	4504.50	7507.45	4707.50	7794.30	4929.13	8039.00	5125.25	8469.35	5282.75	8766.85	5296.75	8870.05	5372.25	9171.08	5395.88	9131.75
53	4418.00	7080.85	4737.50	7669.95	4992.50	8166.30	5198.63	8505.00	5463.25	8796.35	5625.75	9226.35	5760.75	9574.05	5775.25	9689.08	5873.38	9994.75
54	4725.00	7480.85	4910.00	7696.95	5238.00	8312.80	5555.63	8848.00	5757.75	9207.35	5972.25	9525.85	6143.25	9982.55	6266.75	10325.08	6299.88	10468.75
55	5032.50	7860.35	5241.50	8095.95	5454.00	8330.30	5818.13	9008.50	6134.75	9587.85	6330.75	9959.85	6563.75	10303.05	6750.25	10814.08	6860.38	11184.25
56	5161.00	7865.85	5537.50	8524.95	5769.00	8745.80	6027.63	8970.00	6424.75	9742.85	6703.25	10353.85	6913.25	10730.55	7172.75	11138.58	7368.88	11693.25
57	5254.50	7820.85	5728.50	8493.95	6106.00	9198.80	6313.13	9405.50	6598.75	9662.85	7004.75	10475.85	7298.75	11141.55	7534.25	11534.58	7792.88	11930.75
58	5406.00	7889.85	5788.50	8443.45	6302.00	9132.80	6711.63	9911.50	6901.75	10122.35	7127.75	10353.85	7595.75	11254.55	7951.25	12009.58	8161.38	12392.25
59	5826.50	8136.85	5999.00	8519.45	6381.50	9111.30	6904.13	9835.00	7313.75	10617.35	7478.25	10827.85	7727.25	11086.05	8262.25	12038.08	8603.88	12801.25
60	5825.15	7773.30	6445.63	8720.80	6594.25	9162.25	6963.75	9769.50	7548.75	10543.85	8002.25	11352.70	8135.75	11614.40	8397.25	11913.25	8989.75	12890.90
61	5463.15	7114.80	6395.63	8383.30	7049.75	9370.75	7195.25	9825.00	7607.75	10469.35	8185.75	11241.20	8644.25	12148.90	8829.75	12451.25	9079.25	12717.40
62	5248.65	6647.30	5976.63	7658.30	6975.25	9002.75	7658.75	10030.50	7789.75	10503.85	8243.25	11159.70	8866.25	11987.40	9410.25	12918.25	9606.25	13252.40
63	4924.15	6069.80	5750.13	7130.30	6554.25	8223.25	7620.75	9615.00	8357.75	10677.35	8461.75	11169.20	8906.25	11871.90	9597.25	12764.75	10160.75	13728.40
64	4457.65	5234.80	5402.63	6478.80	6301.75	7622.75	7092.25	8788.50	8208.75	10273.85	8993.25	11381.20	9075.25	11860.90	9583.25	12612.25	10357.25	13564.90
65	3957.15	4556.80	4833.63	5595.80	5887.25	6919.75	6824.25	8124.50	7601.75	9355.35	8809.25	10957.20	9693.75	12141.90	9847.75	12620.75	10419.75	13355.90
66	4204.15	4863.80	4291.13	4873.30	5248.75	5968.25	6415.25	7393.00	7403.25	8622.35	8185.25	9847.70	9458.25	11594.90	10423.75	12905.75	10634.25	13361.40
67	4848.15	5453.30	4551.13	5189.80	4639.75	5207.75	5668.75	6322.00	6881.25	7775.85	7923.25	9090.70	8772.75	10442.90	10175.25	12263.25	11184.75	13616.40
68	5112.15	5678.80	5254.13	5791.80	4900.75	5502.75	4940.75	5521.50	6033.75	6670.35	7350.25	8150.70	8537.25	9614.40	9448.25	11078.25	10851.75	12919.40
69	5316.65	5729.30	5477.13	5975.30	5644.25	6111.25	5284.75	5808.00	5307.25	5825.35	6442.25	7030.70	7806.25	8574.40	9088.25	10116.25	10109.75	11634.40
70	5466.30	5882.65	5679.85	6061.90	5874.55	6313.90	6010.60	6415.20	5598.95	6095.15	5645.15	6106.80	6844.55	7344.65	8283.45	8970.30	9643.78	10595.25

© 2023 Chien et al. *JAMA Network Open.*

	2011		2012		2013		2014		2015		2016		2017		2018		2019	
sex / Age	M	F	M	F	M	F	M	F	M	F	M	F	M	F	M	F	M	F
71	5431.30	5808.15	5831.35	6165.90	6086.55	6354.40	6292.60	6609.70	6382.45	6700.15	5930.65	6326.80	5949.55	6327.15	7200.45	7637.30	8730.78	9336.25
72	5334.80	5580.65	5809.35	6047.40	6215.05	6441.90	6425.10	6651.70	6589.95	6875.15	6663.15	6978.80	6208.05	6595.15	6252.95	6584.30	7608.78	7964.25
73	5213.30	5410.65	5619.85	5796.90	6176.55	6341.90	6566.10	6775.20	6755.45	6939.65	6920.15	7139.30	6981.55	7269.15	6529.95	6860.80	6570.78	6844.75
74	5239.80	5189.65	5530.85	5625.40	5944.55	6038.90	6501.60	6600.20	6907.45	7023.65	7075.65	7169.30	7226.55	7395.15	7336.45	7553.30	6851.78	7127.25
75	5185.30	5076.15	5477.35	5402.40	5807.55	5857.90	6295.60	6284.20	6817.95	6848.65	7133.15	7254.80	7281.55	7422.15	7480.95	7658.30	7646.28	7803.75
76	5190.30	4974.15	5385.35	5221.40	5665.55	5578.90	5994.60	6090.70	6495.95	6507.15	7034.65	7014.30	7359.05	7466.15	7491.95	7659.80	7677.78	7861.75
77	5162.80	4691.15	5325.35	5122.90	5561.55	5378.90	5868.10	5722.70	6175.45	6230.15	6575.15	6625.80	7137.05	7155.15	7539.45	7634.30	7670.78	7802.25
78	5250.80	4291.15	5332.85	4769.90	5486.55	5202.90	5662.60	5467.70	5985.45	5849.15	6281.15	6387.30	6658.05	6769.15	7237.95	7331.80	7662.28	7812.25
79	5345.80	4122.15	5301.35	4358.40	5369.05	4834.40	5538.60	5299.70	5738.45	5561.15	6022.15	5945.80	6256.55	6460.15	6692.95	6820.30	7326.78	7399.25
80	5486.30	3970.65	5359.10	4152.30	5301.95	4417.60	5363.65	4866.75	5530.00	5286.10	5704.65	5538.80	5984.45	5952.55	6265.10	6472.30	6712.60	6848.00
81	5409.80	3661.65	5480.60	3967.80	5308.45	4171.10	5236.65	4419.25	5311.00	4867.60	5474.65	5275.80	5636.95	5553.55	5913.60	5937.30	6216.10	6438.50
82	5039.80	3436.65	5368.60	3678.80	5435.45	3939.10	5234.65	4139.25	5100.00	4408.60	5143.65	4857.30	5323.95	5240.55	5516.10	5529.30	5772.60	5941.50
83	4612.30	3083.65	4943.60	3384.30	5216.45	3654.60	5288.65	3901.75	5125.00	4105.10	4964.15	4353.80	4990.95	4741.55	5220.10	5155.80	5387.60	5445.00
84	4138.80	2782.65	4476.10	3023.30	4791.45	3310.60	5029.15	3566.75	5104.00	3828.10	4908.15	4000.80	4772.45	4233.05	4856.10	4612.80	5054.60	5014.50

© 2023 Chien et al. *JAMA Network Open.*

eTable 2. Age- and sex-specific midyear population size for 2011—2019 in Taiwan according to Monthly Bulletin of Interior Statistics.

	2011		2012		2013		2014		2015		2016		2017		2018		2019	
Age \ sex	M	F	M	F	M	F	M	F	M	F	M	F	M	F	M	F	M	F
35	193715	196250	199399	202341	195762	199131	203746	206553	203798	207681	202673	206701	201547	205027	193642	196576	185929	186970
36	177631	181020	193256	196490	198986	202575	195416	199416	203409	206750	203475	207821	202351	206847	201242	205183	193372	196766
37	176466	178937	177369	181249	193059	196641	198869	202663	195338	199481	203347	206868	203394	207977	202227	206996	201099	205345
38	175879	178768	176048	179068	177024	181330	192734	196724	198467	202688	194946	199550	203025	206981	203121	208071	201992	207116
39	178291	181190	175421	178849	175615	179112	176615	181419	192290	196748	198027	202715	194567	199640	202652	207011	202748	208086
40	183952	185500	177794	181255	174958	178822	175142	179110	176162	181438	191848	196761	197590	202702	194163	199622	202263	207011
41	186904	187779	183376	185472	177247	181206	174432	178821	174668	179101	175700	181443	191363	196799	197108	202698	193686	199575
42	187259	187283	186239	187680	182759	185364	176726	181109	173920	178728	174159	179035	175196	181409	190822	196772	196598	202678
43	182985	181958	186554	187194	185606	187578	182158	185283	176122	181044	173323	178677	173605	178976	174655	181348	190248	196690
44	184683	183949	182246	181823	185860	187039	184949	187434	181519	185143	175503	180944	172697	178585	173014	178867	174137	181282
45	189329	189493	183884	183801	181471	181671	185119	186877	184227	187252	180840	184976	174868	180814	172055	178476	172366	178763
46	189708	189548	188475	189275	183033	183558	180680	181490	184369	186656	183455	187051	180069	184828	174163	180659	171418	178350
47	192340	192549	188826	189348	187612	189027	182230	183311	179896	181259	183574	186471	182700	186886	179324	184663	173441	180511
48	191743	192561	191317	192334	187843	189073	186633	188760	181326	183051	179041	181041	182702	186259	181862	186642	178569	184453
49	188713	189319	190702	192383	190291	192054	186855	188754	185611	188467	180340	182771	178140	180803	181829	186023	181005	186413
50	186090	186803	187660	189062	189669	192146	189258	191822	185826	188461	184584	188152	179329	182501	177190	180548	180925	185788
51	183620	185431	184991	186556	186545	188766	188549	191810	188162	191475	184744	188120	183530	187798	178359	182181	176217	180249

© 2023 Chien et al. *JAMA Network Open.*

	2011		2012		2013		2014		2015		2016		2017		2018		2019	
sex / Age	M	F	M	F	M	F	M	F	M	F	M	F	M	F	M	F	M	F
52	178994	181909	182496	185044	183899	186184	185438	188392	187387	191443	186972	191129	183610	187812	182439	187488	177307	181924
53	171023	174742	177839	181602	181285	184684	182667	185791	184218	188009	186121	191109	185723	190813	182416	187478	181318	187161
54	168938	173099	169871	174413	176611	181174	180014	184217	181420	185333	182987	187565	184849	190685	184416	190408	181173	187108
55	169512	173405	167709	172700	168669	174002	175333	180745	178683	183747	180071	184866	181620	187092	183530	190199	183127	190006
56	162277	166954	168201	173037	166421	172282	167362	173522	173936	180197	177256	183233	178686	184377	180228	186612	182154	189796
57	153944	159542	160960	166525	166853	172557	165078	171754	165985	172933	172501	179573	175758	182621	177218	183805	178807	186105
58	148909	154490	152600	159056	159582	165961	165433	171979	163638	171218	164467	172362	170966	178980	174259	182044	175692	183253
59	147523	153766	147547	153941	151201	158469	158138	165352	163907	171347	162069	170603	162915	171739	169368	178308	172633	181365
60	136181	142451	146066	153246	146138	153368	149754	157838	156587	164691	162356	170652	160556	169941	161394	171106	167775	177722
61	119539	125350	134714	141847	144518	152573	144586	152692	148210	157153	155008	163972	160745	169967	158940	169290	159771	170452
62	107576	113264	118256	124801	133253	141199	142957	151848	143012	151950	146507	156367	153263	163231	159033	169225	157289	168585
63	94065	99490	106299	112713	116823	124156	131669	140460	141203	151032	141233	151163	144753	155631	151497	162492	157204	168466
64	79006	84379	92888	98990	104952	112154	115329	123528	129994	139691	139387	150229	139462	150391	142953	154873	149661	161735
65	66399	71831	77892	83908	91653	98384	103511	111480	113766	122814	128252	138920	137513	149418	137711	149574	141199	154052
66	67525	73456	65353	71297	76710	83283	90289	97666	101945	110671	112059	121950	126392	137981	135580	148464	135842	148680
67	72803	79367	66363	72810	64256	70657	75405	82516	88751	96769	100277	109708	110264	120961	124426	136920	133496	147385
68	71645	79138	71387	78601	65116	72115	63032	69989	73973	81746	87144	95882	98558	108742	108412	119912	122312	135809
69	70150	78380	70168	78230	69939	77708	63811	71323	61767	69246	72519	80907	85418	94941	96653	107714	106462	118809
70	68820	78050	68495	77378	68564	77271	68342	76769	62388	70460	60390	68430	70962	79974	83681	93862	94678	106540

© 2023 Chien et al. *JAMA Network Open.*

	2011		2012		2013		2014		2015		2016		2017		2018		2019	
Age \ sex	M	F	M	F	M	F	M	F	M	F	M	F	M	F	M	F	M	F
71	65386	75869	67142	76968	66811	76286	66877	76192	66659	75714	60898	69531	58944	67536	69334	78960	81818	92765
72	60238	70994	63571	74628	65363	75736	65034	75069	65093	75008	64914	74588	59300	68509	57454	66551	67678	77893
73	56345	67163	58345	69702	61607	73342	63367	74450	63129	73803	63183	73753	63018	73375	57627	67434	55866	65530
74	52931	63622	54392	65832	56389	68341	59542	71936	61317	73024	61128	72428	61179	72436	61056	72057	55852	66240
75	49983	60212	50943	62178	52417	64351	54420	66809	57435	70391	59134	71538	58977	70973	59073	71032	58997	70716
76	47807	57349	47900	58657	48849	60652	50323	62784	52278	65218	55159	68741	56854	69865	56721	69440	56870	69565
77	45371	52996	45572	55728	45812	57038	46733	58956	48058	61039	49918	63480	52764	66949	54485	68057	54473	67728
78	44284	49087	43042	51252	43302	53947	43517	55242	44398	57121	45725	59208	47520	61646	50313	65103	52081	66220
79	44467	46539	41818	47292	40668	49448	40933	52070	41202	53335	42082	55192	43329	57215	45116	59597	47870	63040
80	44356	43553	41746	44625	39323	45389	38233	47491	38474	50025	38721	51259	39549	53079	40807	55089	42639	57487
81	42094	39685	41463	41558	39005	42667	36720	43405	35754	45388	35969	47811	36206	49068	37042	50866	38316	52843
82	38203	35905	38983	37639	38511	39481	36228	40565	34066	41312	33095	43165	33332	45459	33679	46768	34492	48518
83	34239	31968	35099	33783	35845	35493	35466	37259	33384	38284	31362	38953	30470	40746	30720	43078	31087	44394
84	30184	28586	31185	29928	32015	31639	32699	33235	32296	34908	30376	35817	28603	36462	27874	38220	28095	40463

© 2023 Chien et al. *JAMA Network Open.*

eTable 3. Age- and sex-specific cancer-free midyear population size in Taiwan from 2011 to 2019.

	2011		2012		2013		2014		2015		2016		2017		2018		2019	
Age \ sex	M	F	M	F	M	F	M	F	M	F	M	F	M	F	M	F	M	F
40	182369	182993	176236	178752	173419	176348	173558	176552	174545	178662	190050	193710	195708	199526	192275	196384	200202	203551
41	185162	184963	181583	182590	175490	178320	172714	175960	172898	176151	173876	178284	189318	193314	194963	199038	191545	195852
42	185342	184154	184247	184456	180717	182110	174751	177837	171974	175456	172160	175659	173141	177780	188527	192759	194197	198462
43	180929	178608	184364	183649	183311	183929	179832	181592	173890	177286	171123	174906	171334	175117	172329	177216	187673	192112
44	182410	180140	179948	177979	183405	182991	182353	183290	178884	180918	172992	176655	170235	174303	170481	174483	171528	176584
45	186800	185141	181327	179474	178883	177259	182373	182258	181360	182540	177951	180143	172068	175887	169274	173555	169535	173728
46	186988	184760	185594	184324	180156	178654	177812	176508	181340	181427	180298	181651	176880	179318	171050	175097	168326	172765
47	189295	187255	185738	183964	184376	183467	178991	177788	176666	175653	180176	180599	179176	180826	175769	178425	169953	174199
48	188441	186866	187948	186428	184399	183053	183029	182565	177735	176898	175484	174798	178938	179708	177925	179857	174620	177470
49	185181	183274	187013	186025	186502	185502	182985	182078	181633	181573	176380	175933	174198	173830	177667	178696	176684	178852
50	182323	180370	183659	182345	185516	185067	184998	184557	181481	181110	180135	180547	174901	174920	172775	172804	176283	177656
51	179600	178545	180773	179435	182054	181405	183879	184055	183378	183485	179917	180059	178599	179451	173425	173819	171347	171739
52	174770	174874	177992	177537	179192	178390	180509	180353	182262	182974	181689	182362	178313	178942	177067	178317	171911	172792
53	166605	167661	173102	173932	176293	176518	177468	177286	178755	179213	180495	181883	179962	181239	176641	177789	175445	177166
54	164213	165618	164961	166716	171373	172861	174458	175369	175662	176126	177015	178039	178706	180702	178149	180083	174873	176639
55	164480	165545	162468	164604	163215	165672	169515	171737	172548	174159	173740	174906	175056	176789	176780	179385	176267	178822
56	157116	159088	162664	164512	160652	163536	161334	164552	167511	170454	170553	172879	171773	173646	173055	175473	174785	178103

© 2023 Chien et al. *JAMA Network Open.*

	2011		2012		2013		2014		2015		2016		2017		2018		2019	
Age \ sex	M	F	M	F	M	F	M	F	M	F	M	F	M	F	M	F	M	F
57	148690	151721	155232	158031	160747	163358	158765	162349	159386	163270	165496	169097	168459	171479	169684	172270	171014	174174
58	143503	146600	146812	150613	153280	156828	158721	162068	156736	161096	157339	162008	163370	167725	166308	170034	167531	170861
59	141697	145629	141548	145422	144820	149358	151234	155517	156593	160730	154591	159775	155188	160653	161106	166270	164029	168564
60	130356	134678	139620	144525	139544	144206	142790	148069	149038	154147	154354	159299	152420	158327	152997	159193	158785	164831
61	114076	118235	128318	133464	137468	143202	137391	142867	140602	146684	146822	152731	152101	157818	150110	156839	150692	157735
62	102327	106617	112279	117143	126278	132196	135298	141818	135222	141446	138264	145207	144397	151244	149623	156307	147683	155333
63	89141	93420	100549	105583	110269	115933	124048	130845	132845	140355	132771	139994	135847	143759	141900	149727	147043	154738
64	74548	79144	87485	92511	98650	104531	108237	114740	121785	129417	130394	138848	130387	138530	133370	142261	139304	148170
65	62442	67274	73058	78312	85766	91464	96687	103356	106164	113459	119443	127963	127819	137276	127863	136953	130779	140696
66	63321	68592	61062	66424	71461	77315	83874	90273	94542	102049	103874	112102	116934	126386	125156	135558	125208	135319
67	67955	73914	61812	67620	59616	65449	69736	76194	81870	88993	92354	100617	101491	110518	114251	124657	122311	133769
68	66533	73459	66133	72809	60215	66612	58091	64468	67939	75076	79794	87731	90021	99128	98964	108834	111460	122890
69	64833	72651	64691	72255	64295	71597	58526	65515	56460	63421	66077	73876	77612	86367	87565	97598	96352	107175
70	63354	72167	62815	71316	62689	70957	62331	70354	56789	64365	54745	62323	64117	72629	75398	84892	85034	95945
71	59955	70061	61311	70802	60724	69932	60584	69582	60277	69014	54967	63204	52994	61209	62134	71323	73087	83429
72	54903	65413	57762	68581	59148	69294	58609	68417	58503	68133	58251	67609	53092	61914	51201	59967	60069	69929
73	51132	61752	52725	63905	55430	67000	56801	67675	56374	66863	56263	66614	56036	66106	51097	60573	49295	58685
74	47691	58432	48861	60207	50444	62302	53040	65336	54410	66000	54052	65259	53952	65041	53720	64504	49000	59113
75	44798	55136	45466	56776	46609	58493	48124	60525	50617	63542	52001	64283	51695	63551	51592	63374	51351	62912

© 2023 Chien et al. *JAMA Network Open.*

	2011		2012		2013		2014		2015		2016		2017		2018		2019	
Age \ sex	M	F	M	F	M	F	M	F	M	F	M	F	M	F	M	F	M	F
76	42617	52375	42515	53436	43183	55073	44328	56693	45782	58711	48124	61727	49495	62399	49229	61780	49192	61703
77	40208	48305	40247	50605	40250	51659	40865	53233	41883	54809	43343	56854	45627	59794	46946	60423	46802	59926
78	39033	44796	37709	46482	37815	48744	37854	49774	38413	51272	39444	52821	40862	54877	43075	57771	44419	58408
79	39121	42417	36517	42934	35299	44614	35394	46770	35464	47774	36060	49246	37072	50755	38423	52777	40543	55641
80	38870	39582	36387	40473	34021	40971	32869	42624	32944	44739	33016	45720	33565	47126	34542	48617	35926	50639
81	36684	36023	35982	37590	33697	38496	31483	38986	30443	40520	30494	42535	30569	43514	31128	44929	32100	46405
82	33163	32468	33614	33960	33076	35542	30993	36426	28966	36903	27951	38308	28008	40218	28163	41239	28719	42577
83	29627	28884	30155	30399	30629	31838	30177	33357	28259	34179	26398	34599	25479	36004	25500	37922	25699	38949
84	26045	25803	26709	26905	27224	28328	27670	29668	27192	31080	25468	31816	23831	32229	23018	33607	23040	35449

© 2023 Chien et al. *JAMA Network Open.*

eTable 4. Age- and sex-specific smoking rate for each year during 2011—2019.

	2011		2012		2013		2014		2015		2016		2017		2018		2019	
sex / Age	M	F	M	F	M	F	M	F	M	F	M	F	M	F	M	F	M	F
35-39	0.549	0.087	0.528	0.087	0.508	0.087	0.487	0.087	0.467	0.086	0.446	0.086	0.426	0.086	0.405	0.086	0.385	0.086
40-44	0.629	0.075	0.604	0.074	0.579	0.074	0.554	0.074	0.529	0.074	0.504	0.074	0.479	0.074	0.454	0.073	0.429	0.073
45-49	0.621	0.064	0.607	0.064	0.593	0.064	0.578	0.064	0.564	0.064	0.550	0.064	0.535	0.064	0.521	0.064	0.507	0.064
50-54	0.607	0.051	0.604	0.051	0.602	0.050	0.600	0.050	0.597	0.050	0.595	0.049	0.593	0.049	0.590	0.048	0.588	0.048
55-59	0.577	0.043	0.571	0.042	0.564	0.040	0.558	0.039	0.552	0.038	0.546	0.036	0.540	0.035	0.534	0.034	0.528	0.032
60-64	0.554	0.035	0.547	0.034	0.540	0.034	0.534	0.034	0.527	0.034	0.520	0.033	0.513	0.033	0.507	0.033	0.500	0.033
65-69	0.583	0.030	0.570	0.028	0.558	0.027	0.545	0.025	0.532	0.024	0.519	0.023	0.507	0.021	0.494	0.020	0.481	0.018
70-74	0.546	0.030	0.526	0.028	0.506	0.027	0.486	0.025	0.466	0.024	0.445	0.023	0.425	0.021	0.405	0.020	0.385	0.018
75-79	0.562	0.030	0.542	0.028	0.522	0.027	0.502	0.025	0.482	0.024	0.462	0.023	0.442	0.021	0.422	0.020	0.402	0.018
80-84	0.528	0.030	0.508	0.028	0.488	0.027	0.468	0.025	0.448	0.024	0.429	0.023	0.409	0.021	0.389	0.020	0.369	0.018

© 2023 Chien et al. *JAMA Network Open.*

Current smoking rate

	**2011**		**2012**		**2013**		**2014**		**2015**		**2016**		**2017**		**2018**		**2019**	
Age \ sex	M	F	M	F	M	F	M	F	M	F	M	F	M	F	M	F	M	F
35-39	0.442	0.069	0.429	0.069	0.416	0.069	0.403	0.069	0.390	0.069	0.377	0.069	0.363	0.069	0.350	0.069	0.337	0.069
40-44	0.482	0.060	0.462	0.059	0.441	0.059	0.420	0.059	0.400	0.059	0.379	0.059	0.359	0.059	0.338	0.058	0.317	0.058
45-49	0.451	0.049	0.444	0.049	0.436	0.049	0.428	0.049	0.420	0.049	0.412	0.049	0.405	0.049	0.397	0.049	0.389	0.049
50-54	0.414	0.038	0.407	0.037	0.400	0.037	0.393	0.036	0.386	0.036	0.379	0.036	0.372	0.035	0.365	0.035	0.358	0.034
55-59	0.352	0.034	0.347	0.032	0.342	0.030	0.337	0.029	0.331	0.027	0.326	0.025	0.321	0.023	0.316	0.022	0.311	0.020
60-64	0.288	0.029	0.285	0.028	0.283	0.027	0.280	0.025	0.278	0.024	0.275	0.023	0.273	0.022	0.270	0.020	0.268	0.019
65-69	0.276	0.015	0.266	0.015	0.256	0.015	0.246	0.015	0.236	0.014	0.226	0.014	0.216	0.014	0.206	0.014	0.196	0.014
70-74	0.329	0.015	0.320	0.015	0.311	0.015	0.303	0.015	0.294	0.014	0.285	0.014	0.276	0.014	0.268	0.014	0.259	0.014
75-79	0.354	0.015	0.353	0.015	0.351	0.015	0.349	0.015	0.348	0.014	0.346	0.014	0.345	0.014	0.343	0.014	0.342	0.014
80-84	0.391	0.015	0.383	0.015	0.376	0.015	0.368	0.015	0.361	0.014	0.353	0.014	0.346	0.014	0.338	0.014	0.331	0.014

© 2023 Chien et al. *JAMA Network Open.*

eTable 5. The midyear age-specific number of cancer-free individuals by sex and smoking status for each year from 2011 to 2019.

	Never-smokers																	
Year	2011		2012		2013		2014		2015		2016		2017		2018		2019	
Sex / Age	M	F	M	F	M	F	M	F	M	F	M	F	M	F	M	F	M	F
40	67600	169338	69742	165446	72972	163253	77379	163474	82192	165459	94255	179431	101964	184854	104992	181979	114337	188657
41	68635	171161	71858	168999	73843	165078	77003	162925	81417	163134	86233	165142	98635	179099	106460	184438	109393	181521
42	68702	170412	72912	170725	76043	168587	77911	164663	80981	162490	85382	162711	90206	164707	102946	178620	110907	183940
43	67066	165280	72958	169978	77135	170271	80176	168140	81883	164185	84868	162013	89265	162240	94101	164217	107182	178055
44	67615	166698	71211	164731	77174	169402	81300	169712	84235	167549	85795	163633	88693	161486	93092	161684	97961	163663
45	70721	173377	71249	168049	72853	165956	76890	170615	79064	170858	80129	168594	79947	164591	81077	162389	83632	162531
46	70792	173021	72925	172591	73372	167262	74967	165233	79055	169816	81186	170006	82183	167802	81927	163832	83036	161630
47	71665	175357	72982	172254	75090	171767	75464	166431	77017	164412	81131	169021	83250	169213	84187	166945	83839	162972
48	71342	174993	73850	174561	75100	171380	77167	170902	77483	165577	79018	163592	83139	168167	85220	168286	86141	166031
49	70108	171629	73483	174183	75957	173673	77148	170447	79182	169953	79422	164654	80937	162666	85097	167199	87159	167325
50	71728	171139	72678	173089	73842	175750	74063	175343	73075	172143	72949	171683	71233	166405	70767	164464	72611	169156
51	70657	169408	71536	170327	72464	172272	73615	174866	73838	174401	72860	171219	72740	170716	71033	165430	70578	163522
52	68756	165924	70435	168525	71324	169409	72266	171349	73389	173914	73578	173409	72623	170231	72525	169711	70810	164525
53	65544	159081	68500	165103	70170	167631	71049	168435	71977	170340	73095	172953	73295	172416	72350	169208	72266	168689
54	64603	157143	65279	158254	68212	164159	69844	166613	70732	167406	71685	169298	72783	171906	72968	171392	72030	168188
55	69635	158442	69776	157758	71095	158998	74875	165044	77269	167601	78865	168549	80532	170595	82405	173335	83243	173025

© 2023 Chien et al. *JAMA Network Open.*

	Never-smokers																	
Year	2011		2012		2013		2014		2015		2016		2017		2018		2019	
Sex / Age	M	F	M	F	M	F	M	F	M	F	M	F	M	F	M	F	M	F
56	66518	152263	69860	157670	69978	156949	71262	158140	75014	164035	77418	166595	79022	167562	80669	169555	82544	172330
57	62950	145212	66668	151458	70020	156778	70127	156022	71375	157122	75123	162951	77497	165471	79098	166460	80763	168528
58	60754	140311	63052	144348	66767	150511	70107	155752	70188	155029	71420	156120	75156	161849	77524	164300	79118	165322
59	59989	139381	60792	139373	63082	143342	66800	149457	70124	154677	70172	153968	71392	155024	75099	160662	77464	163100
60	58193	130020	63262	139561	64160	139286	66607	143053	70518	148962	74065	153978	74156	153076	75459	153951	79375	159442
61	50925	114146	58141	128879	63206	138317	64088	138027	66526	141749	70451	147629	74000	152584	74035	151674	75329	152578
62	45681	102929	50874	113119	58061	127687	63112	137013	63981	136688	66344	140357	70252	146227	73795	151160	73825	150254
63	39794	90189	45559	101956	50700	111978	57865	126412	62856	135633	63708	135318	66092	138991	69986	144797	73505	149679
64	33280	76407	39640	89333	45358	100965	50489	110853	57623	125064	62568	134210	63436	133936	65779	137576	69636	143326
65	26020	65278	31379	76099	37933	89009	44000	100727	49670	110734	57410	125070	63070	134367	64727	134245	67876	138113
66	26387	66557	26226	64547	31606	75239	38169	87977	44232	99598	49927	109568	57699	123708	63357	132878	64984	132834
67	28318	71721	26548	65709	26368	63692	31735	74256	38304	86856	44390	98343	50079	108176	57836	122192	63481	131312
68	27725	71280	28404	70752	26632	64824	26436	62828	31786	73273	38353	85748	44419	97027	50098	106682	57849	120633
69	27017	70495	27785	70213	28437	69675	26634	63849	26415	61897	31760	72206	38296	84537	44327	95668	50008	105207
70	28739	70026	29764	69301	30971	69052	32054	68565	30351	62819	30365	60915	36859	71090	44867	83213	52319	94183
71	27197	67982	29051	68801	30000	68054	31155	67813	32215	67356	30488	61776	30465	59912	36974	69912	44969	81897
72	24906	63472	27370	66643	29222	67434	30139	66677	31267	66497	32309	66081	30521	60602	30468	58781	36959	68645
73	23195	59920	24983	62099	27385	65202	29210	65954	30129	65258	31207	65108	32213	64705	30406	59375	30330	57608

© 2023 Chien et al. *JAMA Network Open.*

	Never-smokers																	
Year	2011		2012		2013		2014		2015		2016		2017		2018		2019	
Age \ Sex	M	F	M	F	M	F	M	F	M	F	M	F	M	F	M	F	M	F
74	21634	56699	23152	58505	24922	60630	27276	63674	29079	64415	29981	63784	31015	63663	31967	63228	30149	58027
75	19621	53500	20825	55171	22282	56923	23970	58986	26225	62016	27984	62830	28855	62204	29830	62120	30719	61757
76	18666	50821	19473	51926	20644	53595	22079	55252	23720	57301	25898	60332	27626	61077	28464	60558	29428	60570
77	17611	46872	18434	49175	19242	50272	20354	51880	21700	53493	23324	55569	25467	58527	27144	59228	27998	58825
78	17097	43467	17272	45169	18078	47436	18855	48509	19902	50040	21226	51627	22808	53714	24906	56629	26572	57335
79	17135	41158	16726	41720	16875	43416	17629	45581	18374	46626	19405	48133	20693	49679	22216	51733	24254	54619
80	18353	38408	17902	39329	17413	39872	17476	41540	18169	43664	18863	44687	19842	46128	21105	47655	22664	49709
81	17321	34954	17703	36528	17247	37463	16739	37994	16789	39547	17422	41574	18071	42592	19019	44040	20250	45552
82	15659	31505	16538	33001	16929	34588	16478	35499	15975	36017	15970	37442	16557	39366	17208	40423	18117	41795
83	13989	28027	14837	29540	15677	30984	16044	32509	15585	33358	15082	33817	15062	35242	15580	37172	16212	38234
84	12298	25038	13141	26144	13934	27568	14711	28914	14996	30333	14551	31097	14088	31546	14064	32943	14535	34798

	Ever-smokers																	
Year	2011		2012		2013		2014		2015		2016		2017		2018		2019	
Age \ Sex	M	F	M	F	M	F	M	F	M	F	M	F	M	F	M	F	M	F
40	114769	13655	106494	13306	100447	13095	96179	13078	92353	13202	95795	14279	93744	14672	87282	14405	85865	14894
41	116526	13802	109725	13592	101646	13242	95711	13034	91481	13017	87643	13142	90683	14215	88502	14600	82152	14331

© 2023 Chien et al. *JAMA Network Open.*

	Ever-smokers																	
Year	2011		2012		2013		2014		2015		2016		2017		2018		2019	
Sex / Age	M	F	M	F	M	F	M	F	M	F	M	F	M	F	M	F	M	F
42	116640	13741	111335	13731	104674	13523	96840	13173	90993	12965	86777	12949	82934	13073	85581	14139	83289	14522
43	113863	13328	111405	13670	106176	13658	99655	13452	92006	13101	86255	12893	82069	12877	78228	12999	80491	14057
44	114795	13442	108737	13248	106231	13588	101053	13577	94649	13369	87197	13022	81542	12817	77389	12799	73567	12921
45	116079	11764	110078	11424	106029	11304	105483	11643	102296	11682	97822	11549	92121	11296	88197	11166	85902	11197
46	116196	11740	112668	11733	106784	11392	102845	11276	102285	11611	99112	11646	94697	11517	89123	11265	85289	11135
47	117630	11898	112756	11710	109285	11699	103527	11358	99649	11241	99045	11578	95926	11613	91581	11480	86114	11227
48	117099	11874	114097	11867	109299	11673	105862	11663	100251	11321	96466	11206	95798	11542	92705	11572	88479	11438
49	115073	11645	113530	11841	110545	11829	105837	11632	102450	11620	96958	11279	93261	11164	92570	11497	89524	11527
50	110595	9230	110981	9256	111674	9317	110935	9214	108407	8967	107186	8864	103668	8515	102008	8340	103672	8500
51	108943	9137	109237	9108	109590	9132	110264	9189	109540	9085	107056	8840	105860	8735	102392	8389	100769	8217
52	106013	8949	107556	9012	107867	8981	108243	9004	108873	9059	108111	8953	105690	8711	104542	8606	101101	8268
53	101061	8580	104601	8829	106122	8886	106420	8851	106778	8873	107401	8930	106668	8823	104291	8581	103179	8477
54	99610	8476	99682	8462	103161	8702	104615	8756	104931	8720	105330	8741	105923	8796	105181	8691	102843	8452
55	94844	7102	92691	6846	92120	6673	94640	6692	95279	6559	94875	6357	94524	6194	94374	6050	93023	5796
56	90598	6825	92803	6842	90674	6587	90073	6412	92498	6419	93135	6284	92751	6084	92386	5918	92241	5773
57	85739	6509	88563	6573	90727	6580	88638	6327	88011	6148	90374	6146	90962	6008	90586	5810	90251	5646
58	82749	6289	83759	6264	86513	6317	88614	6316	86548	6067	85919	5889	88214	5877	88784	5735	88413	5538
59	81707	6248	80756	6048	81738	6016	84434	6060	86469	6053	84418	5807	83796	5629	86007	5608	86565	5464

© 2023 Chien et al. *JAMA Network Open.*

	Ever-smokers																	
Year	2011		2012		2013		2014		2015		2016		2017		2018		2019	
Age \ Sex	M	F	M	F	M	F	M	F	M	F	M	F	M	F	M	F	M	F
60	72163	4658	76358	4964	75384	4919	76183	5016	78520	5185	80289	5321	78265	5251	77538	5242	79410	5389
61	63150	4089	70177	4584	74262	4885	73302	4840	74076	4934	76372	5102	78101	5234	76075	5165	75363	5157
62	56647	3687	61405	4024	68217	4510	72186	4804	71242	4758	71920	4850	74145	5016	75828	5147	73858	5078
63	49347	3231	54990	3627	59569	3955	66184	4433	69989	4721	69063	4676	69755	4768	71914	4930	73538	5059
64	41269	2737	47846	3178	53292	3566	57748	3887	64162	4354	67826	4638	66951	4594	67591	4685	69667	4844
65	36421	1996	41680	2213	47833	2455	52687	2628	56494	2725	62033	2892	64749	2909	63136	2708	62904	2583
66	36934	2035	34836	1877	39855	2075	45705	2296	50309	2451	53947	2534	59235	2678	61799	2681	60224	2485
67	39637	2193	35264	1911	33249	1757	38001	1938	43566	2137	47964	2274	51412	2342	56415	2465	58831	2456
68	38808	2180	37729	2057	33583	1788	31655	1639	36153	1803	41441	1983	45601	2100	48866	2152	53611	2256
69	37816	2156	36906	2042	35858	1922	31892	1666	30044	1523	34317	1670	39315	1830	43238	1930	46345	1968
70	34615	2141	33051	2015	31718	1905	30278	1789	26438	1546	24380	1409	27259	1539	30531	1679	32715	1762
71	32757	2079	32259	2001	30724	1877	29429	1770	28062	1657	24479	1429	22530	1297	25160	1410	28118	1532
72	29997	1941	30392	1938	29926	1860	28469	1740	27236	1636	25942	1528	22571	1312	20733	1186	23110	1284
73	27937	1832	27742	1806	28045	1799	27591	1721	26245	1606	25056	1506	23823	1401	20691	1198	18965	1078
74	26057	1734	25709	1701	25523	1672	25765	1662	25330	1585	24072	1475	22937	1378	21753	1276	18852	1085
75	25176	1636	24641	1604	24328	1570	24154	1539	24392	1526	24017	1453	22841	1347	21762	1253	20632	1155
76	23951	1554	23042	1510	22539	1478	22249	1442	22062	1410	22227	1395	21869	1322	20765	1222	19764	1133
77	22597	1433	21813	1430	21008	1387	20511	1354	20183	1316	20018	1285	20160	1267	19802	1195	18804	1100

© 2023 Chien et al. *JAMA Network Open.*

	Ever-smokers																	
Year	2011		2012		2013		2014		2015		2016		2017		2018		2019	
Sex / Age	M	F	M	F	M	F	M	F	M	F	M	F	M	F	M	F	M	F
78	21937	1329	20437	1314	19738	1309	19000	1266	18511	1231	18218	1194	18054	1163	18169	1142	17847	1072
79	21986	1259	19791	1213	18424	1198	17765	1189	17090	1147	16655	1113	16380	1075	16207	1044	16289	1022
80	20517	1174	18484	1144	16608	1100	15394	1084	14775	1074	14153	1033	13722	999	13437	961	13263	930
81	19363	1069	18279	1062	16449	1033	14745	991	13654	973	13072	961	12498	922	12109	888	11850	852
82	17505	963	17076	960	16146	954	14515	926	12991	886	11982	866	11451	852	10955	816	10602	782
83	15638	857	15319	859	14952	855	14133	848	12674	821	11316	782	10417	763	9919	750	9487	715
84	13747	766	13568	760	13290	760	12959	754	12196	746	10917	719	9743	683	8954	665	8506	651

© 2023 Chien et al. *JAMA Network Open.*

eTable 6. Age- and sex-specific number of patients diagnosed with invasive lung ADC for each year from 2011 to 2019 according to the TCR.

Year	2011		2012		2013		2014		2015		2016		2017		2018		2019	
Age \ Sex	M	F	M	F	M	F	M	F	M	F	M	F	M	F	M	F	M	F
40-44	87	93	92	98	101	99	100	99	95	120	79	118	89	108	114	166	113	201
45-49	147	178	170	195	167	213	179	238	178	225	208	222	172	201	197	277	202	290
50	31	62	48	55	48	60	42	56	45	56	61	63	39	47	68	84	64	98
51	42	55	48	61	66	60	61	72	55	63	55	90	41	55	74	76	69	91
52	41	68	68	65	59	77	52	73	66	74	56	83	54	63	59	103	62	101
53	55	51	59	72	51	67	58	87	62	75	77	87	61	69	62	101	59	104
54	45	60	55	50	60	73	76	80	71	96	84	100	67	63	74	103	87	112
55	65	59	59	61	72	66	84	83	69	83	81	98	84	72	100	121	84	134
56	62	68	56	98	78	74	88	83	88	117	82	100	67	75	93	120	113	144
57	64	73	87	73	84	85	91	89	81	88	84	111	88	90	119	146	107	130
58	69	78	80	83	85	102	93	103	80	84	95	112	99	101	125	144	112	145
59	73	72	104	92	88	87	93	116	104	119	100	122	81	96	133	119	122	147
60	85	83	102	104	102	91	88	120	106	125	130	121	107	99	115	142	153	154
61	80	91	91	101	90	103	110	118	115	130	126	146	89	111	128	140	137	183
62	70	92	64	105	83	119	90	132	90	143	107	139	92	93	161	154	142	167
63	57	66	75	98	96	96	101	112	129	133	127	133	104	100	127	178	156	165
64	64	66	75	94	102	97	88	114	120	152	117	137	95	115	133	180	154	180
65	57	62	68	76	85	98	94	112	89	120	118	149	99	117	155	175	147	168
66	47	61	49	60	69	87	90	98	112	117	123	140	112	126	150	164	164	172
67	63	61	63	66	63	63	78	116	119	100	111	139	108	116	168	187	157	174
68	60	74	86	69	64	61	64	92	96	77	109	122	103	119	120	149	168	168
69	75	74	79	90	75	70	73	62	78	68	90	118	88	70	145	131	133	157
70	75	87	86	90	106	77	82	76	47	80	80	91	78	79	94	136	141	150
71	80	63	74	94	83	90	87	102	91	86	68	68	64	72	95	117	108	126
72	76	91	91	81	81	98	82	88	95	94	85	108	69	52	90	84	103	125
73	77	61	91	92	82	104	86	105	77	95	110	107	74	90	78	88	95	94
74	83	65	82	101	84	94	85	97	113	106	96	109	74	84	101	110	82	104
75	61	75	79	99	73	86	97	94	82	97	96	109	85	86	100	102	113	110
76	80	63	79	74	64	80	82	86	82	113	87	101	87	89	81	101	105	104
77	66	80	68	86	68	75	78	80	80	87	74	92	71	104	77	104	89	88
78	68	54	95	79	69	75	67	89	77	90	93	81	78	97	98	116	79	119

© 2023 Chien et al. *JAMA Network Open.*

**Year**	**2011**		**2012**		**2013**		**2014**		**2015**		**2016**		**2017**		**2018**		**2019**	
Age \ Sex	M	F	M	F	M	F	M	F	M	F	M	F	M	F	M	F	M	F
79	80	57	62	69	78	72	75	76	86	87	76	90	73	71	88	103	85	114
80	82	63	69	77	61	70	68	61	50	84	65	71	60	91	74	96	70	87
81	93	52	62	68	84	42	61	71	61	67	65	65	52	67	63	63	55	87
82	86	62	71	54	71	69	64	52	60	57	57	91	73	73	49	94	57	75
83	61	50	55	68	78	51	60	62	62	70	61	58	54	65	54	86	49	77
84	55	47	76	37	62	38	63	69	73	72	55	51	50	50	47	71	42	73

© 2023 Chien et al. *JAMA Network Open.*

eTable 7A. Age-, sex-, and calendar year-specific percentages of patients diagnosed with late-stage (stage2—4) ADC among those with all stage (1—4) ADC, estimated using the TCRLF for each year from 2011 to 2019.

Year	2011		2012		2013		2014		2015		2016		2017		2018		2019	
Age \ Sex	M	F	M	F	M	F	M	F	M	F	M	F	M	F	M	F	M	F
40-44	0.940	0.816	0.857	0.765	0.840	0.646	0.838	0.667	0.716	0.534	0.718	0.465	0.721	0.434	0.607	0.479	0.664	0.426
45-49	0.890	0.803	0.730	0.665	0.834	0.729	0.777	0.639	0.723	0.586	0.722	0.546	0.784	0.682	0.686	0.515	0.652	0.447
50-54	0.875	0.786	0.811	0.715	0.739	0.645	0.798	0.618	0.707	0.617	0.688	0.557	0.729	0.571	0.669	0.484	0.626	0.442
55-59	0.836	0.775	0.819	0.725	0.801	0.713	0.770	0.714	0.749	0.584	0.712	0.577	0.751	0.642	0.686	0.498	0.679	0.477
60-64	0.834	0.753	0.805	0.695	0.759	0.709	0.779	0.726	0.728	0.661	0.723	0.589	0.785	0.664	0.693	0.567	0.678	0.534
65-69	0.872	0.789	0.805	0.742	0.796	0.738	0.796	0.668	0.777	0.667	0.742	0.627	0.761	0.687	0.710	0.603	0.708	0.545
70-74	0.864	0.822	0.782	0.806	0.839	0.768	0.835	0.752	0.789	0.707	0.752	0.705	0.833	0.791	0.735	0.668	0.713	0.669
75-79	0.889	0.858	0.869	0.813	0.862	0.806	0.822	0.812	0.815	0.786	0.835	0.762	0.853	0.797	0.790	0.702	0.754	0.717
80-84	0.908	0.905	0.927	0.889	0.854	0.852	0.858	0.897	0.887	0.888	0.860	0.866	0.881	0.857	0.850	0.822	0.826	0.809

© 2023 Chien et al. *JAMA Network Open.*

eTable 7B. Age-, sex- and calendar year-specific percentages of ever-smokers among corresponding patients with late-stage (2—4) ADC in Taiwan.

Year	2011		2012		2013		2014		2015		2016		2017		2018		2019	
Age \ Sex	M	F	M	F	M	F	M	F	M	F	M	F	M	F	M	F	M	F
40-44	0.743	0.085	0.761	0.155	0.741	0.049	0.709	0.167	0.677	0.200	0.714	0.151	0.672	0.174	0.667	0.141	0.676	0.207
45-49	0.748	0.076	0.761	0.105	0.738	0.090	0.752	0.117	0.705	0.142	0.768	0.221	0.748	0.097	0.733	0.120	0.589	0.154
50-54	0.708	0.059	0.726	0.120	0.728	0.097	0.768	0.144	0.761	0.126	0.716	0.160	0.725	0.138	0.748	0.122	0.750	0.156
55-59	0.710	0.031	0.730	0.043	0.726	0.069	0.711	0.087	0.730	0.080	0.737	0.079	0.777	0.106	0.742	0.088	0.769	0.073
60-64	0.685	0.057	0.709	0.061	0.673	0.069	0.714	0.074	0.663	0.051	0.684	0.069	0.702	0.085	0.686	0.058	0.709	0.087
65-69	0.730	0.054	0.675	0.052	0.734	0.068	0.669	0.053	0.700	0.077	0.649	0.076	0.706	0.060	0.671	0.058	0.675	0.062
70-74	0.683	0.055	0.698	0.044	0.690	0.049	0.667	0.049	0.667	0.057	0.677	0.064	0.654	0.034	0.672	0.039	0.656	0.062
75-79	0.720	0.055	0.692	0.027	0.673	0.062	0.683	0.059	0.695	0.048	0.688	0.057	0.603	0.050	0.634	0.048	0.647	0.054
80-84	0.693	0.061	0.668	0.044	0.672	0.060	0.654	0.043	0.690	0.060	0.656	0.038	0.639	0.040	0.609	0.039	0.608	0.021

© 2023 Chien et al. *JAMA Network Open.*

eTable 7C. Age-, sex- and year-specific percentages of ever-smokers among corresponding patients with early-stage (1) ADC in Taiwan.

Year	2011		2012		2013		2014		2015		2016		2017		2018		2019	
Age \ Sex	M	F	M	F	M	F	M	F	M	F	M	F	M	F	M	F	M	F
40-44	0.600	0.063	0.538	0.043	0.250	0.059	0.375	0.000	0.556	0.094	0.318	0.017	0.333	0.083	0.279	0.106	0.351	0.081
45-49	0.667	0.000	0.628	0.063	0.385	0.036	0.432	0.073	0.327	0.054	0.596	0.103	0.472	0.098	0.459	0.076	0.443	0.045
50-54	0.680	0.098	0.510	0.060	0.507	0.078	0.517	0.044	0.566	0.044	0.510	0.086	0.565	0.057	0.458	0.047	0.500	0.051
55-59	0.640	0.014	0.522	0.037	0.468	0.053	0.480	0.053	0.534	0.050	0.537	0.031	0.564	0.047	0.526	0.044	0.506	0.059
60-64	0.561	0.021	0.653	0.027	0.468	0.091	0.586	0.044	0.530	0.004	0.528	0.033	0.520	0.018	0.475	0.044	0.522	0.044
65-69	0.657	0.060	0.683	0.067	0.609	0.042	0.513	0.072	0.581	0.032	0.478	0.016	0.522	0.037	0.473	0.039	0.484	0.029
70-74	0.551	0.083	0.570	0.024	0.516	0.030	0.530	0.064	0.541	0.031	0.433	0.014	0.625	0.027	0.414	0.052	0.466	0.021
75-79	0.500	0.024	0.622	0.015	0.511	0.015	0.552	0.014	0.643	0.022	0.552	0.075	0.528	0.060	0.523	0.034	0.514	0.034
80-84	0.484	0.000	0.619	0.036	0.617	0.056	0.611	0.074	0.567	0.057	0.595	0.049	0.688	0.045	0.718	0.000	0.489	0.014

© 2023 Chien et al. *JAMA Network Open.*

eTable 7D. Age-, sex-, and calendar year-specific percentages of patients with stage 3&4 ADC among patients with all stages (1—4) of invasive ADC, estimated using the TCRLF for each year from 2011 to 2019.

Year	2011		2012		2013		2014		2015		2016		2017		2018		2019	
Age \ Sex	M	F	M	F	M	F	M	F	M	F	M	F	M	F	M	F	M	F
40-44	0.892	0.793	0.824	0.714	0.790	0.604	0.768	0.656	0.684	0.500	0.641	0.447	0.698	0.425	0.580	0.442	0.645	0.400
45-49	0.846	0.775	0.693	0.634	0.785	0.667	0.749	0.597	0.678	0.550	0.678	0.505	0.760	0.646	0.644	0.485	0.597	0.412
50-54	0.832	0.751	0.785	0.688	0.707	0.606	0.749	0.596	0.656	0.584	0.664	0.524	0.709	0.543	0.632	0.455	0.590	0.412
55-59	0.790	0.745	0.782	0.684	0.758	0.683	0.728	0.671	0.720	0.563	0.679	0.546	0.733	0.608	0.643	0.451	0.637	0.447
60-64	0.799	0.729	0.762	0.646	0.711	0.673	0.731	0.677	0.684	0.622	0.677	0.565	0.749	0.630	0.647	0.535	0.639	0.482
65-69	0.843	0.748	0.769	0.722	0.747	0.681	0.750	0.627	0.725	0.627	0.702	0.584	0.740	0.657	0.664	0.556	0.672	0.492
70-74	0.810	0.784	0.757	0.763	0.804	0.732	0.808	0.717	0.726	0.658	0.700	0.662	0.816	0.774	0.696	0.639	0.672	0.629
75-79	0.841	0.819	0.825	0.777	0.835	0.770	0.767	0.780	0.773	0.757	0.786	0.738	0.808	0.765	0.745	0.671	0.727	0.673
80-84	0.859	0.880	0.884	0.867	0.827	0.816	0.841	0.876	0.859	0.860	0.831	0.850	0.837	0.835	0.816	0.791	0.791	0.762

© 2023 Chien et al. *JAMA Network Open.*

eTable 7E. Age-, sex-, and year-specific percentages of ever-smokers among corresponding patients with stage 3&4 ADC in Taiwan.

Year	2011		2012		2013		2014		2015		2016		2017		2018		2019	
Age \ Sex	M	F	M	F	M	F	M	F	M	F	M	F	M	F	M	F	M	F
40-44	0.729	0.087	0.765	0.152	0.750	0.035	0.708	0.169	0.661	0.196	0.740	0.137	0.661	0.178	0.667	0.139	0.667	0.221
45-49	0.752	0.079	0.766	0.109	0.756	0.098	0.758	0.111	0.693	0.142	0.759	0.221	0.748	0.094	0.732	0.120	0.593	0.150
50-54	0.710	0.062	0.722	0.125	0.726	0.098	0.777	0.134	0.763	0.129	0.725	0.166	0.723	0.137	0.758	0.125	0.750	0.152
55-59	0.702	0.033	0.725	0.045	0.724	0.068	0.710	0.086	0.727	0.083	0.734	0.080	0.775	0.108	0.737	0.091	0.772	0.075
60-64	0.682	0.059	0.711	0.062	0.680	0.063	0.724	0.075	0.664	0.052	0.677	0.069	0.701	0.089	0.685	0.057	0.711	0.083
65-69	0.734	0.053	0.665	0.053	0.748	0.069	0.684	0.053	0.696	0.082	0.651	0.079	0.710	0.063	0.670	0.056	0.680	0.064
70-74	0.683	0.057	0.699	0.047	0.677	0.049	0.671	0.048	0.657	0.058	0.681	0.064	0.658	0.034	0.681	0.038	0.659	0.066
75-79	0.723	0.058	0.688	0.028	0.673	0.061	0.685	0.058	0.684	0.047	0.682	0.059	0.599	0.045	0.635	0.047	0.654	0.055
80-84	0.694	0.063	0.662	0.045	0.672	0.057	0.659	0.044	0.698	0.058	0.654	0.039	0.629	0.041	0.603	0.041	0.596	0.022

© 2023 Chien et al. *JAMA Network Open.*

eTable 8A. Estimated age- and sex-specific number of patients with late-stage lung ADC in the TCR by smoking status.

	Never-smokers																	
Year	2011		2012		2013		2014		2015		2016		2017		2018		2019	
Age \ Sex	M	F	M	F	M	F	M	F	M	F	M	F	M	F	M	F	M	F
40-44	20.992	69.483	18.881	63.380	21.996	60.793	24.409	55.000	21.935	51.310	16.205	46.579	21.037	38.717	23.071	68.270	24.262	67.817
45-49	32.971	132.183	29.655	116.066	36.442	141.176	34.516	134.354	37.984	113.083	34.898	94.446	34.005	123.869	36.084	125.448	54.147	109.651
50-54	54.702	218.836	61.690	190.674	57.053	196.443	53.456	194.694	50.516	196.353	65.027	198.061	52.449	146.252	56.876	198.298	53.345	188.739
55-59	80.655	262.664	85.372	282.331	89.145	274.728	99.941	308.775	85.317	263.664	82.810	288.473	70.251	248.774	100.915	294.932	84.349	309.762
60-64	93.623	282.481	95.477	327.821	117.578	334.214	106.131	400.839	137.291	428.126	138.826	370.686	114.052	314.764	144.471	423.872	146.428	413.721
65-69	71.105	247.797	90.370	254.085	75.421	260.646	105.197	303.639	115.239	296.851	143.222	387.005	114.110	353.651	172.470	458.143	176.826	428.777
70-74	107.150	285.103	100.222	352.757	113.202	338.032	117.453	334.553	111.298	307.180	106.795	318.704	103.388	288.022	110.306	343.313	129.619	376.231
75-79	88.253	266.539	102.636	321.943	99.272	293.329	104.011	324.686	101.270	354.355	110.878	339.927	133.470	338.588	128.478	351.701	125.387	362.780
80-84	105.115	232.676	102.492	258.422	99.726	216.238	93.720	270.241	84.183	292.093	89.658	279.799	91.856	284.420	95.416	323.828	88.457	316.151

	Ever-smokers																	
Year	2011		2012		2013		2014		2015		2016		2017		2018		2019	
Age \ Sex	M	F	M	F	M	F	M	F	M	F	M	F	M	F	M	F	M	F
40-44	60.767	6.414	59.976	11.620	62.844	3.144	59.430	11.000	46.065	12.828	40.513	8.281	43.126	8.151	46.143	11.209	50.729	17.737
45-49	97.815	10.835	94.456	13.593	102.895	14.010	104.593	17.844	90.739	18.674	115.268	26.831	100.917	13.272	98.973	17.155	77.505	20.032

© 2023 Chien et al. *JAMA Network Open.*

	Ever-smokers																	
Year	2011		2012		2013		2014		2015		2016		2017		2018		2019	
Sex / Age	M	F	M	F	M	F	M	F	M	F	M	F	M	F	M	F	M	F
50-54	132.548	13.810	163.799	26.048	152.860	21.010	177.140	32.630	161.021	28.350	164.167	37.726	138.466	23.316	168.480	27.571	160.035	34.795
55-59	197.873	8.507	230.820	12.642	236.660	20.390	246.009	29.507	230.673	22.837	231.869	24.757	244.258	29.641	290.261	28.542	281.163	24.399
60-64	203.388	17.055	232.183	21.218	241.570	24.717	265.328	32.153	270.259	23.142	299.907	27.380	268.294	29.205	315.696	26.230	356.381	39.351
65-69	192.205	14.254	187.518	13.821	207.947	18.994	212.563	17.046	268.529	24.828	265.350	31.635	273.865	22.712	351.572	28.113	367.500	28.306
70-74	230.869	16.512	231.193	16.331	252.441	17.560	234.906	17.212	222.596	18.717	223.500	21.755	195.778	10.047	226.184	14.035	247.357	24.795
75-79	227.421	15.613	230.361	8.943	204.249	19.403	223.850	20.364	230.477	17.999	244.811	20.504	202.467	17.762	222.323	17.640	229.691	20.824
80-84	237.119	15.175	206.250	11.800	204.202	13.802	177.443	12.173	187.338	18.546	170.835	11.192	162.892	12.001	148.426	13.144	136.927	6.799

© 2023 Chien et al. *JAMA Network Open.*

eTable 8B. Estimated age- and sex-specific number of patients with early-stage lung ADC in the TCR by smoking status.

	Never-smokers																	
Year	2011		2012		2013		2014		2015		2016		2017		2018		2019	
Sex / Age	M	F	M	F	M	F	M	F	M	F	M	F	M	F	M	F	M	F
40-44	2.096	16.034	6.066	22.000	12.120	33.000	10.101	33.000	12.000	50.592	15.192	62.088	16.558	56.038	32.287	77.360	24.655	106.086
45-49	5.404	34.983	17.075	61.257	17.023	55.749	22.641	79.524	33.186	88.176	23.337	90.338	19.569	57.578	33.510	124.137	39.194	153.123
50-54	8.560	57.123	25.741	81.142	36.522	110.272	28.195	134.515	37.936	133.105	50.844	171.022	30.907	120.121	60.517	229.892	63.810	268.138
55-59	19.610	77.764	33.341	107.840	43.167	112.625	53.628	128.521	49.403	194.325	59.002	222.558	45.521	148.324	84.760	312.285	85.218	344.198
60-64	25.872	96.369	27.504	148.855	60.581	133.699	43.709	155.787	71.621	230.711	79.488	268.635	50.234	170.960	107.064	328.726	114.440	378.491
65-69	13.265	65.774	21.306	86.819	28.421	95.219	39.578	147.861	46.192	155.117	74.356	245.272	58.360	165.358	112.834	307.367	115.928	370.685
70-74	23.787	59.936	39.833	86.795	34.079	104.217	32.710	108.839	40.884	130.914	61.669	140.490	22.438	76.826	71.230	168.411	81.218	193.914
75-79	19.663	45.732	18.890	74.978	23.701	74.144	31.854	78.840	26.876	99.460	31.482	104.153	27.388	85.254	44.481	151.402	56.395	146.175
80-84	17.944	26.149	9.241	32.571	19.942	37.740	17.437	30.172	14.941	37.112	17.233	42.814	10.704	47.326	12.173	73.028	24.337	74.963

	Ever-smokers																	
Year	2011		2012		2013		2014		2015		2016		2017		2018		2019	
Sex / Age	M	F	M	F	M	F	M	F	M	F	M	F	M	F	M	F	M	F
40-44	3.145	1.069	7.077	1.000	4.040	2.063	6.061	0.000	15.000	5.270	7.090	1.052	8.279	5.094	12.498	9.161	13.355	9.360
45-49	10.809	0.000	28.814	4.084	10.639	2.065	17.250	6.278	16.090	5.068	34.498	10.384	17.509	6.281	28.433	10.259	31.154	7.194

© 2023 Chien et al. *JAMA Network Open.*

	Ever-smokers																	
Year	2011		2012		2013		2014		2015		2016		2017		2018		2019	
Sex / Age	M	F	M	F	M	F	M	F	M	F	M	F	M	F	M	F	M	F
50-54	18.190	6.232	26.770	5.136	37.565	9.275	30.209	6.161	49.527	6.191	52.962	16.191	40.179	7.312	51.127	11.239	63.810	14.328
55-59	34.862	1.065	36.467	4.188	38.028	6.257	49.422	7.197	56.607	10.174	68.319	7.213	58.970	7.261	94.063	14.241	87.271	21.641
60-64	33.116	2.095	51.835	4.106	53.270	13.370	61.832	7.222	80.829	1.021	88.779	9.299	54.420	3.071	96.769	15.172	124.750	17.437
65-69	25.425	4.176	45.807	6.276	44.211	4.140	41.661	11.454	64.039	5.205	68.072	4.088	63.665	6.279	101.124	12.377	108.747	11.233
70-74	29.194	5.449	52.752	2.117	36.278	3.190	36.931	7.397	48.222	4.189	47.036	2.051	37.396	2.105	50.280	9.242	70.805	4.061
75-79	19.663	1.115	31.113	1.136	24.778	1.123	39.286	1.110	48.377	2.186	38.828	8.416	30.675	5.396	48.717	5.257	59.528	5.221
80-84	16.823	0.000	15.017	1.206	32.129	2.220	27.401	2.414	19.538	2.249	25.275	2.196	23.548	2.254	30.985	0.000	23.279	1.086

© 2023 Chien et al. *JAMA Network Open.*

eTable 8C. Estimated age- and sex-specific number of patients with stage 3&4 lung ADC in the TCR by smoking status.

	Never-smokers																	
Year	2011		2012		2013		2014		2015		2016		2017		2018		2019	
Sex / Age	M	F	M	F	M	F	M	F	M	F	M	F	M	F	M	F	M	F
40-44	21.054	67.345	17.841	59.394	19.948	57.714	22.391	53.957	22.034	48.214	13.167	45.544	21.048	37.698	22.054	63.242	24.312	62.649
45-49	30.800	127.017	27.536	110.039	31.986	128.015	32.452	126.207	37.050	106.132	33.933	87.252	32.975	117.608	34.055	118.269	49.093	101.498
50-54	51.607	208.435	60.693	182.442	55.018	184.070	48.344	189.728	46.432	185.151	60.847	184.858	51.446	139.200	51.624	186.062	50.282	176.573
55-59	78.396	252.149	83.079	265.867	85.057	263.263	94.694	290.555	83.034	253.519	79.755	273.004	69.243	235.449	96.633	266.422	78.174	289.301
60-64	90.552	273.137	89.693	304.192	107.513	319.009	96.379	373.322	128.802	402.704	132.707	355.411	108.875	297.465	135.224	400.749	137.215	375.271
65-69	67.849	235.343	88.809	246.834	67.035	240.219	94.672	285.137	108.891	277.128	134.857	359.587	109.575	337.128	161.468	423.487	165.489	386.156
70-74	100.457	271.422	96.723	333.116	113.135	322.402	112.161	319.324	105.348	285.631	98.028	299.342	100.229	281.774	101.803	329.166	121.358	351.765
75-79	82.586	253.856	98.456	307.119	96.105	280.624	96.399	312.081	99.529	341.996	106.544	328.536	127.550	326.278	120.736	336.155	118.616	340.256
80-84	98.962	225.965	99.410	251.913	96.532	207.698	90.678	263.743	79.344	283.695	87.152	274.551	89.639	277.093	92.947	311.234	87.314	297.363

	Ever-smokers																	
Year	2011		2012		2013		2014		2015		2016		2017		2018		2019	
Sex / Age	M	F	M	F	M	F	M	F	M	F	M	F	M	F	M	F	M	F
40-44	56.513	6.414	57.983	10.606	59.843	2.099	54.377	11.012	42.966	11.786	37.474	7.246	41.045	8.151	44.107	10.200	48.624	17.751
45-49	93.501	10.856	90.317	13.495	99.155	13.985	101.543	15.776	83.628	17.517	107.101	24.775	97.827	12.205	92.878	16.128	71.504	17.974

© 2023 Chien et al. *JAMA Network Open.*

	Ever-smokers																	
Year	2011		2012		2013		2014		2015		2016		2017		2018		2019	
Age \ Sex	M	F	M	F	M	F	M	F	M	F	M	F	M	F	M	F	M	F
50-54	126.384	13.825	157.589	26.063	145.635	19.985	168.154	29.441	149.850	27.352	160.125	36.762	134.391	22.146	161.326	26.580	150.847	31.720
55-59	184.715	8.511	218.740	12.660	223.276	19.342	232.330	27.471	220.724	22.858	220.433	23.740	238.023	28.636	270.158	26.536	264.750	23.399
60-64	194.041	17.071	220.451	20.209	228.865	21.410	252.318	30.124	254.356	22.138	278.154	26.366	255.857	29.100	294.059	24.066	337.095	33.923
65-69	186.863	13.135	176.495	13.831	198.942	17.911	204.578	16.019	249.361	24.898	251.882	30.671	267.971	22.691	328.465	24.972	351.263	26.233
70-74	216.369	16.483	224.170	16.329	237.584	16.505	228.765	16.127	201.916	17.577	209.272	20.607	192.748	10.063	217.031	12.866	234.048	24.894
75-79	216.082	15.520	217.519	8.967	197.931	18.326	209.810	19.292	215.260	16.875	228.466	20.534	190.761	15.484	210.170	16.587	223.929	19.821
80-84	224.802	15.064	194.997	11.772	197.832	12.623	175.146	12.154	183.557	17.369	164.621	11.206	152.264	11.944	141.185	13.218	128.546	6.836

© 2023 Chien et al. *JAMA Network Open.*

eTable 9. Number of patients with invasive lung ADC in TCR, TCRLF, having known smoking status (ever-smoker or never-smoker), and having a known number of pack-years smoked and a known number of years since smoking quitting, for 2011—2019.

Year	TCR	TCRLF	Smoking status known	Pack-year and quit time known
2011	5738	5504	5157	1426
2012	6445	6275	5863	1583
2013	6650	6503	6068	1596
2014	7129	6990	6509	1679
2015	7516	7412	6946	1773
2016	8049	7926	7478	1858
2017	7003	6848	6385	1629
2018	9259	9130	8628	2000
2019	9663	9549	9061	2003
Sum	67452	66137	62095	15547

© 2023 Chien et al. *JAMA Network Open.*

eTable 10A. Late-stage ADC age-specific incidence rates for each single year and for the period 2011—2019 by sex and smoking status: never-smoking female (1), never-smoking male (2), ever-smoking female (3), ever-smoking male (4).

(1) never-smoking female

Age	Never-smoking Female														
	2011	2012	2013	2014	2015	2016	2017	2018	2019	2011-2019		2011-2015		2016-2019	
	rate									rate	95% Cl	rate	95% Cl	rate	95% Cl
40-44	8.243	7.546	7.267	6.635	6.236	5.592	4.542	7.839	7.570	6.839	(6.28-7.39)	7.192	(6.38-8.03)	6.413	(5.50-7.30)
45-49	15.222	13.470	16.608	15.926	13.452	11.299	14.880	15.139	13.364	14.380	(13.51-15.26)	14.935	(13.67-16.09)	13.668	(12.42-14.98)
50-54	26.600	22.827	23.132	22.729	22.880	23.069	17.172	23.601	22.628	22.722	(21.67-23.70)	23.614	(22.15-25.04)	21.609	(20.06-23.19)
55-59	35.707	37.614	35.838	39.364	33.021	35.694	30.320	35.350	36.775	35.487	(34.16-36.89)	36.295	(34.57-38.38)	34.549	(32.71-36.58)
60-64	54.990	57.226	54.060	61.163	62.219	52.100	43.427	57.345	54.777	55.135	(53.34-57.02)	58.181	(55.34-60.82)	51.968	(49.44-54.69)
65-69	71.756	73.156	71.914	77.928	68.659	78.830	64.557	77.433	68.266	72.313	(69.71-74.96)	72.614	(68.46-76.45)	72.064	(68.41-75.49)
70-74	89.627	108.424	102.319	100.562	94.127	100.328	90.015	102.632	104.404	99.276	(95.47-102.89)	99.068	(93.76-104.05)	99.532	(94.33-104.91)
75-79	113.028	132.399	116.566	124.780	131.498	122.060	118.719	121.164	123.770	122.700	(118.14-127.19)	123.847	(118.15-129.57)	121.440	(114.47-127.89)
80-84	147.326	157.056	126.845	153.148	159.683	148.343	145.951	160.126	150.485	150.101	(144.35-155.75)	148.965	(140.56-156.86)	151.317	(143.00-159.96)

© 2023 Chien et al. *JAMA Network Open.*

(2) never-smoking male

Age	Never-smoking Male											
	2011	2012	2013	2014	2015	2016	2017	2018	2019	2011-2019	2011-2015	2016-2019
	rate									**rate 95% CI**	**rate 95% CI**	**rate 95% CI**
40-44	6.181	5.264	5.832	6.199	5.341	3.712	4.488	4.600	4.495	5.038 (4.31-5.78)	5.756 (4.68-6.81)	4.345 (3.44-5.24)
45-49	9.297	8.136	9.786	9.044	9.695	8.705	8.305	8.643	12.776	9.404 (8.33-10.32)	9.200 (7.78-10.51)	9.635 (8.11-11.08)
50-54	16.028	17.705	16.026	14.815	13.916	17.856	14.462	15.814	14.889	15.714 (14.44-17.08)	15.677 (13.85-17.57)	15.760 (13.64-17.79)
55-59	25.217	25.859	26.147	28.298	23.441	22.201	18.314	25.561	20.923	23.869 (22.19-25.53)	25.785 (23.42-28.28)	21.764 (19.30-24.25)
60-64	41.086	37.082	41.771	35.124	42.703	41.178	32.780	40.237	39.397	38.980 (36.85-41.30)	39.561 (36.25-43.15)	38.408 (35.17-41.53)
65-69	52.489	64.393	49.956	63.002	60.522	64.561	45.002	61.520	58.129	57.695 (54.23-61.11)	58.321 (53.18-63.51)	57.232 (52.55-61.70)
70-74	85.262	74.614	79.440	78.389	72.724	69.191	64.187	63.147	66.565	71.892 (67.19-76.25)	77.878 (71.45-83.93)	65.726 (60.01-72.13)
75-79	97.917	110.684	102.215	101.092	92.130	94.095	106.394	96.922	90.225	98.616 (92.30-104.40)	100.538 (92.13-109.38)	96.775 (88.19-105.08)
80-84	135.423	127.921	122.816	115.067	103.275	109.489	109.848	109.704	96.383	113.999 (105.74-121.55)	120.735 (109.98-131.62)	106.136 (95.57-117.06)

© 2023 Chien et al. *JAMA Network Open.*

(3) ever-smoking female

Age	Ever-smoking Female														
	2011	2012	2013	2014	2015	2016	2017	2018	2019	2011-2019		2011-2015		2016-2019	
	rate									rate	95% CI	rate	95% CI	rate	95% CI
40-44	9.437	17.202	4.686	16.587	19.538	12.493	12.048	16.258	25.079	14.861	(11.67-17.76)	13.451	(9.56-17.33)	16.585	(12.06-21.56)
45-49	18.389	23.207	24.198	30.995	32.491	46.860	23.230	30.108	35.440	29.372	(24.50-33.76)	25.808	(19.97-31.67)	33.915	(26.33-41.69)
50-54	31.123	58.318	46.669	72.487	63.419	85.108	53.501	64.710	83.017	61.902	(54.27-70.17)	54.451	(44.70-64.35)	71.571	(59.74-84.67)
55-59	25.800	38.810	63.375	92.769	73.089	81.215	99.496	98.015	86.467	72.282	(61.78-81.89)	58.395	(46.03-69.66)	91.265	(75.67-109.66)
60-64	92.677	104.128	113.201	139.920	96.615	111.361	117.460	104.216	154.159	115.773	(100.63-130.48)	109.985	(91.12-129.25)	121.988	(99.88-143.77)
65-69	134.979	136.836	189.986	167.660	233.356	278.647	191.514	235.528	240.931	203.036	(174.87-233.81)	172.824	(134.08-207.92)	236.190	(191.91-277.21)
70-74	169.747	172.615	192.688	198.268	233.072	296.136	145.052	207.971	367.848	215.683	(182.76-248.72)	191.792	(151.07-228.83)	254.420	(198.12-320.59)
75-79	216.514	126.470	279.523	299.912	271.430	318.365	287.693	301.238	379.831	271.431	(230.39-313.97)	237.616	(187.62-285.76)	320.341	(250.51-396.64)
80-84	314.207	246.613	293.513	264.373	412.010	256.587	284.476	322.174	173.017	286.490	(234.94-337.41)	305.243	(234.82-375.72)	260.013	(186.87-343.60)

© 2023 Chien et al. *JAMA Network Open.*

(4) ever-smoking male

Age	Ever-smoking Male														
	2011	2012	2013	2014	2015	2016	2017	2018	2019	2011-2019		2011-2015		2016-2019	
	rate									rate	95% CI	rate	95% CI	rate	95% CI
40-44	10.539	10.951	12.105	12.142	9.982	9.131	10.007	11.066	12.514	10.943	(9.93-11.86)	11.143	(9.87-12.53)	10.637	(9.13-12.20)
45-49	16.805	16.773	18.986	19.977	17.900	23.553	21.390	21.792	17.805	19.332	(18.04-20.71)	18.049	(16.45-19.80)	21.217	(18.86-23.18)
50-54	25.189	30.786	28.391	32.775	29.900	30.681	26.234	32.499	31.284	29.747	(28.23-31.31)	29.427	(27.43-31.32)	30.157	(27.81-32.54)
55-59	45.421	52.630	53.571	55.110	51.397	51.673	54.250	64.198	62.412	54.565	(52.31-56.94)	51.648	(48.62-54.68)	58.146	(54.56-61.89)
60-64	71.977	74.711	73.043	76.773	75.493	82.061	73.062	85.567	95.843	79.100	(76.10-82.61)	74.507	(70.47-79.01)	84.174	(79.34-88.77)
65-69	101.365	100.592	109.229	106.313	123.994	110.700	105.206	128.567	130.359	114.166	(109.41-118.82)	108.734	(102.15-115.37)	119.226	(112.38-125.74)
70-74	152.527	155.003	172.979	165.974	166.976	180.345	164.354	190.283	203.151	171.359	(163.82-178.76)	162.486	(152.50-172.05)	184.590	(172.22-196.21)
75-79	196.652	209.947	192.621	215.906	225.435	242.064	203.888	229.897	246.089	217.250	(207.70-226.77)	207.763	(195.79-218.86)	230.304	(215.38-245.34)
80-84	273.274	249.315	263.673	247.323	282.604	278.053	281.674	268.039	254.944	265.996	(253.21-278.97)	262.964	(246.51-277.94)	271.106	(249.63-295.12)

© 2023 Chien et al. *JAMA Network Open.*

eTable 10B. Early-stage ADC age-specific incidence rates for each single year and for the period 2011—2019 by sex and smoking status: never-smoking female (1), never-smoking male (2), ever-smoking female (3), ever-smoking male (4).

(1) never-smoking female

	Never-smoking Female											
**Age**	2011	2012	2013	2014	2015	2016	2017	2018	2019	2011-2019	2011-2015	2016-2019
	rate									**rate 95% CI**	**rate 95% rate**	**rate 95% CI**
40-44	1.902	2.619	3.945	3.981	6.149	7.454	6.574	8.882	11.842	5.984 (5.40-6.52)	3.707 (3.14-4.31)	8.736 (7.71-9.65)
45-49	4.029	7.109	6.558	9.426	10.489	10.808	6.917	14.981	18.662	9.824 (9.10-10.55)	7.497 (6.64-8.32)	12.816 (11.58-14.08)
50-54	6.943	9.714	12.985	15.703	15.510	19.920	14.104	27.361	32.148	17.161 (16.21-18.05)	12.225 (11.23-13.29)	23.317 (21.72-24.91)
55-59	10.571	14.367	14.692	16.384	24.337	27.538	18.077	37.430	40.864	23.084 (22.00-24.28)	16.192 (14.94-17.47)	31.082 (29.29-33.01)
60-64	18.760	25.985	21.626	23.771	33.529	37.757	23.587	44.473	50.113	31.983 (30.51-33.38)	25.110 (23.29-26.90)	39.130 (36.89-41.29)
65-69	19.047	24.997	26.272	37.948	35.877	49.960	30.185	51.950	59.017	39.643 (37.79-41.47)	29.343 (26.74-31.75)	48.203 (45.16-50.96)
70-74	18.842	26.677	31.545	32.715	40.115	44.226	24.010	50.346	53.811	36.095 (33.99-38.27)	30.052 (27.25-32.89)	43.500 (39.70-46.83)
75-79	19.393	30.835	29.464	30.299	36.908	37.399	29.893	52.159	49.871	35.729 (33.23-38.09)	29.608 (26.66-32.53)	42.455 (38.27-46.20)
80-84	16.557	19.795	22.138	17.099	20.289	22.699	24.285	36.111	35.682	24.384 (21.96-26.76)	19.212 (16.31-21.94)	29.923 (26.39-33.68)

© 2023 Chien et al. *JAMA Network Open.*

(2) never-smoking male

Age	Never-smoking Male											
	2011	2012	2013	2014	2015	2016	2017	2018	2019	2011-2019	2011-2015	2016-2019
	rate									**rate 95% CI**	**rate 95% c CI**	**rate 95% CI**
40-44	0.617	1.691	3.213	2.565	2.922	3.480	3.532	6.437	4.568	3.425 (2.85-4.05)	2.254 (1.60-2.93)	4.556 (3.65-5.50)
45-49	1.524	4.685	4.572	5.933	8.470	5.821	4.779	8.026	9.248	5.998 (5.20-6.74)	5.112 (4.08-6.11)	7.000 (5.63-8.17)
50-54	2.508	7.388	10.259	7.814	10.450	13.962	8.522	16.827	17.809	10.672 (9.58-11.79)	7.739 (6.44-9.10)	14.264 (12.32-16.20)
55-59	6.131	10.099	12.661	15.185	13.573	15.818	11.867	21.469	21.139	14.518 (13.21-15.85)	11.659 (10.01-13.11)	17.658 (15.50-19.94)
60-64	11.354	10.682	21.522	14.465	22.277	23.578	14.438	29.818	30.791	20.686 (19.03-22.41)	16.490 (14.46-18.55)	24.808 (22.18-27.54)
65-69	9.792	15.181	18.825	23.703	24.260	33.518	23.016	40.248	38.110	27.669 (25.22-29.93)	18.971 (15.69-22.06)	34.103 (30.57-37.64)
70-74	18.928	29.656	23.915	21.831	26.714	39.954	13.930	40.777	41.709	29.337 (26.54-32.15)	24.284 (20.41-28.07)	34.542 (30.23-38.70)
75-79	21.816	20.371	24.403	30.960	24.450	26.717	21.832	33.556	40.580	27.861 (24.62-31.06)	24.551 (20.09-28.82)	31.030 (25.83-35.93)
80-84	23.118	11.534	24.559	21.409	18.329	21.044	12.800	13.995	26.518	19.292 (16.08-22.38)	19.782 (15.68-24.14)	18.720 (14.23-23.24)

© 2023 Chien et al. *JAMA Network Open.*

(3) ever-smoking female

Age	Ever-smoking Female											
	2011	2012	2013	2014	2015	2016	2017	2018	2019	2011-2019	2011-2015	2016-2019
	rate									rate 95% CI	rate 95% CI	rate 95% CI
40-44	1.573	1.480	3.073	0.000	8.027	1.588	7.530	13.288	13.235	5.602 (3.78-7.56)	2.810 (1.20-4.48)	9.016 (5.48-12.43)
45-49	0.000	6.972	3.566	10.905	8.817	18.135	10.994	18.005	12.727	9.957 (7.14-12.73)	6.023 (3.10-8.61)	14.971 (10.09-20.18)
50-54	14.044	11.498	20.603	13.686	13.849	36.527	16.778	26.379	34.185	20.713 (16.41-25.23)	14.744 (9.38-19.22)	28.458 (20.88-37.10)
55-59	3.231	12.857	19.447	22.627	32.562	23.661	24.372	48.905	76.693	28.463 (22.27-34.84)	17.964 (11.20-23.64)	42.815 (31.46-54.42)
60-64	11.384	20.152	61.233	31.428	4.262	37.820	12.352	60.281	68.310	35.049 (27.44-43.33)	25.862 (15.81-35.33)	44.914 (31.95-57.92)
65-69	39.546	62.139	41.409	112.656	48.925	36.006	52.951	103.694	95.612	66.315 (50.83-83.37)	60.724 (38.86-83.55)	72.451 (44.78-93.83)
70-74	56.015	22.375	35.008	85.206	52.166	27.919	30.387	136.951	60.249	54.690 (37.10-68.71)	49.634 (28.88-68.87)	62.888 (36.02-93.66)
75-79	15.468	16.066	16.184	16.354	32.964	130.685	87.397	89.773	95.222	52.837 (34.13-71.68)	19.256 (5.77-31.75)	101.408 (62.63-137.78)
80-84	0.000	25.212	47.209	52.423	49.969	50.336	53.421	0.000	27.647	34.052 (15.00-49.99)	34.537 (12.81-59.77)	33.367 (6.03-60.28)

© 2023 Chien et al. *JAMA Network Open.*

(4) ever-smoking male

Age	Ever-smoking Male											
	2011	2012	2013	2014	2015	2016	2017	2018	2019	2011-2019	2011-2015	2016-2019
	rate									rate 95% CI	rate 95% CI	rate 95% CI
40-44	0.545	1.292	0.778	1.238	3.250	1.598	1.921	2.997	3.294	1.784 (1.40-2.17)	1.361 (0.93-1.81)	2.429 (1.71-3.24)
45-49	1.857	5.117	1.963	3.295	3.174	7.049	3.711	6.260	7.157	4.273 (3.68-4.88)	3.076 (2.43-3.72)	6.030 (4.81-7.13)
50-54	3.457	5.031	6.977	5.589	9.197	9.898	7.612	9.862	12.474	7.766 (6.94-8.62)	6.064 (5.08-6.99)	9.942 (8.65-11.32)
55-59	8.003	8.315	8.608	11.071	12.613	15.225	13.097	20.804	19.372	13.058 (11.96-14.25)	9.741 (8.41-11.03)	17.131 (15.10-19.04)
60-64	11.719	16.679	16.107	17.891	22.579	24.292	14.820	26.228	33.550	20.818 (19.25-22.41)	17.257 (15.18-19.29)	24.752 (21.99-27.48)
65-69	13.409	24.573	23.223	20.837	29.570	28.399	24.457	36.980	38.574	27.609 (25.46-29.73)	22.499 (19.64-25.64)	32.368 (28.99-35.72)
70-74	19.287	35.368	24.859	26.093	36.173	37.954	31.393	42.300	58.152	33.934 (30.71-37.18)	28.196 (24.26-32.16)	42.491 (36.59-48.59)
75-79	17.003	28.356	23.368	37.892	47.318	38.393	30.890	50.377	63.778	36.750 (32.77-40.63)	30.376 (26.24-35.17)	45.520 (39.18-51.99)
80-84	19.388	18.153	41.487	38.192	29.474	41.137	40.720	55.956	43.343	34.891 (30.16-39.46)	28.809 (23.12-34.03)	45.144 (36.35-54.29)

© 2023 Chien et al. *JAMA Network Open.*

eTable 10C. Stage 3&4 ADC age-specific incidence rates for each single year and for the period 2011—2019 by sex and smoking status: never-smoking female (1), never-smoking male (2), ever-smoking female (3), ever-smoking male (4).

(1) never-smoking female

Age	Never-smoking Female											
	2011	2012	2013	2014	2015	2016	2017	2018	2019	2011-2019	2011-2015	2016-2019
	rate									rate 95% CI	rate 95% CI	rate 95% CI
40-44	7.990	7.072	6.899	6.509	5.860	5.468	4.423	7.261	6.993	6.503 (5.96-7.03)	6.872 (6.07-7.72)	6.058 (5.21-6.92)
45-49	14.627	12.771	15.060	14.960	12.625	10.439	14.128	14.272	12.370	13.480 (12.64-14.35)	14.010 (12.85-15.15)	12.800 (11.55-14.05)
50-54	25.336	21.842	21.675	22.149	21.574	21.531	16.344	22.145	21.170	21.515 (20.44-22.49)	22.497 (21.08-23.85)	20.289 (18.76-21.78)
55-59	34.278	35.420	34.343	37.041	31.751	33.780	28.696	31.933	34.346	33.462 (32.14-34.84)	34.553 (32.82-36.52)	32.196 (30.35-34.22)
60-64	53.172	53.102	51.600	56.965	58.524	49.953	41.040	54.217	49.686	51.869 (50.08-53.72)	54.864 (52.23-57.34)	48.755 (46.27-51.42)
65-69	68.150	71.068	66.278	73.180	64.097	73.245	61.540	71.576	61.480	67.488 (65.17-70.00)	68.439 (64.62-72.40)	66.697 (63.23-70.05)
70-74	85.326	102.387	97.588	95.984	87.524	94.233	88.062	98.403	97.615	94.220 (90.68-97.70)	93.817 (88.62-98.54)	94.712 (89.76-100.04)
75-79	107.649	126.303	111.518	119.936	126.911	117.970	114.403	115.808	116.086	117.427 (113.11-121.79)	118.676 (113.23-124.49)	116.055 (109.85-122.31)
80-84	143.077	153.100	121.836	149.466	155.092	145.560	142.191	153.898	141.542	145.210 (139.49-151.44)	144.665 (136.81-152.64)	145.793 (137.47-153.80)

© 2023 Chien et al. *JAMA Network Open.*

(2) never-smoking male

Age	Never-smoking Male											
	2011	2012	2013	2014	2015	2016	2017	2018	2019	2011-2019	2011-2015	2016-2019
	rate									rate 95% CI	rate 95% CI	rate 95% CI
40-44	6.199	4.974	5.289	5.686	5.365	3.016	4.490	4.397	4.504	4.804 (4.10-5.49)	5.493 (4.47-6.54)	4.139 (3.24-4.98)
45-49	8.685	7.555	8.590	8.503	9.456	8.465	8.053	8.157	11.584	8.812 (7.79-9.70)	8.570 (7.24-9.81)	9.085 (7.57-10.47)
50-54	15.121	17.419	15.454	13.398	12.791	16.709	14.185	14.354	14.034	14.818 (13.56-16.12)	14.811 (13.05-16.61)	14.826 (12.80-16.89)
55-59	24.511	25.164	24.948	26.812	22.813	21.382	18.051	24.477	19.392	22.929 (21.27-24.64)	24.838 (22.54-27.17)	20.830 (18.40-23.22)
60-64	39.738	34.836	38.195	31.896	40.062	39.363	31.292	37.661	36.918	36.595 (34.60-38.80)	36.889 (33.44-39.84)	36.306 (33.06-39.91)
65-69	50.085	63.281	44.401	56.699	57.188	60.791	43.214	57.596	54.402	54.153 (50.76-57.53)	54.485 (49.36-59.42)	53.907 (49.44-58.30)
70-74	79.936	72.009	79.393	74.857	68.836	63.510	62.226	58.279	62.322	68.281 (63.88-72.51)	74.830 (68.62-80.95)	61.536 (55.93-67.46)
75-79	91.629	106.176	98.954	93.694	90.546	90.416	101.675	91.081	85.353	93.938 (87.63-99.84)	95.999 (87.46-104.91)	91.964 (83.72-100.03)
80-84	127.496	124.074	118.882	111.333	97.338	106.429	107.197	106.864	95.137	110.160 (101.72-117.80)	115.681 (104.75-125.90)	103.715 (93.53-114.45)

© 2023 Chien et al. *JAMA Network Open.*

(3) ever-smoking female

Age	Ever-smoking Female														
	2011	2012	2013	2014	2015	2016	2017	2018	2019	2011-2019		2011-2015		2016-2019	
	rate									rate	95% CI	rate	95% CI	rate	95% CI
40-44	9.437	15.702	3.127	16.605	17.951	10.931	12.048	14.795	25.098	14.019	(11.02-17.10)	12.528	(8.97-16.14)	15.843	(11.33-20.47)
45-49	18.425	23.039	24.155	27.403	30.478	43.269	21.362	28.304	31.797	27.532	(22.77-31.83)	24.662	(18.94-30.30)	31.190	(24.13-39.05)
50-54	31.156	58.351	44.392	65.402	61.185	82.932	50.816	62.385	75.679	59.028	(51.49-66.89)	52.135	(42.90-62.12)	67.975	(56.26-80.03)
55-59	25.813	38.867	60.117	86.365	73.157	77.877	96.121	91.127	82.924	69.383	(58.91-78.67)	56.503	(44.18-67.18)	86.990	(71.42-103.73)
60-64	92.762	99.173	98.056	131.091	92.424	107.235	117.037	95.619	132.893	108.047	(93.89-122.28)	103.166	(83.69-120.88)	113.289	(91.87-132.81)
65-69	124.388	136.942	179.149	157.555	234.021	270.151	191.343	209.213	223.293	193.534	(166.73-224.63)	166.707	(130.19-202.09)	222.974	(179.12-262.28)
70-74	169.453	172.592	181.112	185.777	218.879	280.514	145.285	190.658	369.326	208.112	(174.51-241.84)	184.439	(144.40-224.38)	246.494	(187.31-309.79)
75-79	215.216	126.811	264.014	284.135	254.476	318.831	250.802	283.246	361.528	258.383	(216.74-296.95)	227.971	(173.20-274.23)	302.369	(233.80-375.75)
80-84	311.924	246.018	268.422	263.961	385.873	256.912	283.121	323.991	173.958	280.377	(227.43-334.90)	294.509	(222.02-358.65)	260.424	(186.87-343.60)

© 2023 Chien et al. *JAMA Network Open.*

(4) ever-smoking male

Age	Ever-smoking Male														
	2011	2012	2013	2014	2015	2016	2017	2018	2019	2011-2019		2011-2015		2016-2019	
	rate									rate	95% CI	rate	95% CI	rate	95% CI
40-44	9.801	10.587	11.526	11.110	9.310	8.447	9.524	10.578	11.995	10.321	(9.41-11.28)	10.472	(9.21-11.72)	10.091	(8.60-11.61)
45-49	16.063	16.038	18.296	19.395	16.497	21.884	20.735	20.450	16.426	18.332	(17.14-19.55)	17.226	(15.75-18.77)	19.955	(17.78-21.99)
50-54	24.017	29.619	27.049	31.112	27.826	29.925	25.462	31.119	29.487	28.401	(26.95-29.99)	27.941	(25.94-29.86)	28.988	(26.71-31.25)
55-59	42.401	49.875	50.541	52.045	49.180	49.125	52.865	59.751	58.769	51.664	(49.42-53.93)	48.833	(45.81-51.60)	55.138	(51.46-58.45)
60-64	68.669	70.935	69.201	73.008	71.051	76.109	69.675	79.702	90.657	74.656	(71.72-77.97)	70.655	(66.60-74.89)	79.076	(74.45-83.82)
65-69	98.548	94.679	104.499	102.319	115.143	105.081	102.942	120.117	124.599	108.709	(104.16-113.08)	103.390	(96.96-110.18)	113.663	(107.17-120.33)
70-74	142.947	150.295	162.799	161.635	151.464	168.865	161.810	182.584	192.220	162.817	(155.52-170.13)	153.724	(143.36-163.04)	176.378	(164.58-188.55)
75-79	186.847	198.243	186.662	202.365	210.551	225.902	192.099	217.330	239.916	205.855	(196.28-214.70)	196.642	(184.43-208.44)	218.533	(203.60-234.06)
80-84	259.079	235.713	255.448	244.122	276.901	267.939	263.297	254.963	239.340	254.831	(241.63-267.88)	253.609	(238.46-269.10)	256.890	(237.35-278.07)

© 2023 Chien et al. *JAMA Network Open.*

eTable 11A. Age-specific late-stage lung ADC incidence rate ratios and their 95% confidence intervals comparing sex (1), smoking status (2), periods (3).

(1) sex

Age	Never-smoking Female/Male, 2011-2019		Ever-smoking Female/Male, 2011-2019	
	IRR	95% CI	IRR	95% CI
40-44	1.357	(1.16-1.65)	1.358	(1.07-1.73)
45-49	1.529	(1.35-1.76)	1.519	(1.28-1.85)
50-54	1.446	(1.31-1.63)	2.081	(1.86-2.36)
55-59	1.487	(1.38-1.63)	1.325	(1.14-1.55)
60-64	1.414	(1.33-1.53)	1.464	(1.25-1.62)
65-69	1.253	(1.14-1.33)	1.778	(1.47-2.05)
70-74	1.381	(1.30-1.50)	1.259	(1.07-1.50)
75-79	1.244	(1.14-1.34)	1.249	(1.08-1.47)
80-84	1.317	(1.23-1.44)	1.077	(0.90-1.32)

Age	Never-smoking Female/Male, 2011-2015		Ever-smoking Female/Male, 2011-2015	
	IRR	95% CI	IRR	95% CI
40-44	1.249	(1.00-1.64)	1.207	(0.85-1.66)
45-49	1.623	(1.42-1.94)	1.430	(1.14-1.75)
50-54	1.506	(1.34-1.80)	1.850	(1.47-2.23)
55-59	1.408	(1.26-1.56)	1.131	(0.93-1.35)
60-64	1.471	(1.34-1.64)	1.476	(1.21-1.78)
65-69	1.245	(1.10-1.41)	1.589	(1.30-1.95)

© 2023 Chien et al. *JAMA Network Open.*

70-74	1.272	(1.12-1.40)	1.180	(0.92-1.45)
75-79	1.232	(1.11-1.38)	1.144	(0.88-1.44)
80-84	1.234	(1.09-1.37)	1.161	(0.87-1.42)

**Age**	**Never-smoking Female/Male, 2016-2019**		**Ever-smoking Female/Male, 2016-2019**	
	**IRR**	**95% CI**	**IRR**	**95% CI**
40-44	1.476	(1.14-1.98)	1.559	(1.13-2.07)
45-49	1.419	(1.22-1.70)	1.598	(1.19-2.06)
50-54	1.371	(1.19-1.59)	2.373	(2.00-2.82)
55-59	1.587	(1.42-1.79)	1.570	(1.26-1.85)
60-64	1.353	(1.22-1.51)	1.449	(1.23-1.78)
65-69	1.259	(1.10-1.36)	1.981	(1.57-2.41)
70-74	1.514	(1.34-1.78)	1.378	(0.96-1.72)
75-79	1.255	(1.13-1.38)	1.391	(1.02-1.75)
80-84	1.426	(1.27-1.61)	0.959	(0.64-1.30)

(2) smoking status

**Age**	**Ever/never smoking Female, 2011-2019**		**Ever/never smoking Male, 2011-2019**	
	**IRR**	**95% CI**	**IRR**	**95% CI**
40-44	2.173	(1.64-2.68)	2.172	(1.84-2.61)
45-49	2.043	(1.77-2.48)	2.056	(1.83-2.36)
50-54	2.724	(2.42-3.09)	1.893	(1.71-2.12)
55-59	2.037	(1.75-2.40)	2.286	(2.13-2.51)

© 2023 Chien et al. *JAMA Network Open.*

60-64	2.100	(1.77-2.36)	2.029	(1.91-2.20)
65-69	2.808	(2.42-3.29)	1.979	(1.87-2.17)
70-74	2.173	(1.79-2.60)	2.384	(2.24-2.64)
75-79	2.212	(1.82-2.61)	2.203	(2.06-2.37)
80-84	1.909	(1.63-2.43)	2.333	(2.14-2.51)

**Age**	**Ever/never smoking Female, 2011-2015**		**Ever/never smoking Male, 2011-2015**	
	**IRR**	**95% CI**	**IRR**	**95% CI**
40-44	1.870	(1.31-2.51)	1.936	(1.47-2.40)
45-49	1.728	(1.36-2.10)	1.962	(1.65-2.38)
50-54	2.306	(1.85-2.75)	1.877	(1.62-2.18)
55-59	1.609	(1.21-2.01)	2.003	(1.79-2.22)
60-64	1.890	(1.54-2.41)	1.883	(1.70-2.05)
65-69	2.380	(1.82-2.86)	1.864	(1.63-2.13)
70-74	1.936	(1.55-2.34)	2.086	(1.90-2.33)
75-79	1.919	(1.50-2.32)	2.067	(1.85-2.30)
80-84	2.049	(1.64-2.56)	2.178	(1.92-2.47)

**Age**	**Ever/never smoking Female, 2016-2019**		**Ever/never smoking Male, 2016-2019**	
	**IRR**	**95% CI**	**IRR**	**95% CI**
40-44	2.586	(1.71-3.48)	2.448	(1.89-3.23)
45-49	2.481	(2.03-3.17)	2.202	(1.85-2.65)
50-54	3.312	(2.60-3.98)	1.914	(1.68-2.31)
55-59	2.642	(2.06-3.15)	2.672	(2.38-3.05)

© 2023 Chien et al. *JAMA Network Open.*

60-64	2.347	(1.94-2.83)	2.192	(1.97-2.43)
65-69	3.278	(2.73-4.03)	2.083	(1.89-2.30)
70-74	2.556	(1.96-3.09)	2.808	(2.47-3.13)
75-79	2.638	(1.98-3.27)	2.380	(2.11-2.72)
80-84	1.718	(1.17-2.15)	2.554	(2.23-2.90)

(3) periods

Age	Never-smoking Female, 2016-2019/2011-2015		Never-smoking Male, 2016-2019/2011-2015	
	**IRR**	**95% CI**	**IRR**	**95% CI**
40-44	0.892	(0.73-1.05)	0.755	(0.57-1.08)
45-49	0.915	(0.83-1.05)	1.047	(0.81-1.28)
50-54	0.915	(0.84-0.99)	1.005	(0.81-1.24)
55-59	0.952	(0.88-1.05)	0.844	(0.73-0.96)
60-64	0.893	(0.82-0.95)	0.971	(0.87-1.13)
65-69	0.992	(0.91-1.07)	0.981	(0.87-1.08)
70-74	1.005	(0.95-1.09)	0.844	(0.73-0.96)
75-79	0.981	(0.92-1.04)	0.963	(0.87-1.11)
80-84	1.016	(0.94-1.10)	0.879	(0.77-0.99)
**Age**	**Ever-smoking Female, 2016-2019/2011-2015**		**Ever-smoking Male, 2016-2019/2011-2015**	
	**IRR**	**95% CI**	**IRR**	**95% CI**
40-44	1.233	(0.79-1.80)	0.955	(0.77-1.18)
45-49	1.314	(0.97-1.84)	1.176	(1.03-1.34)

© 2023 Chien et al. *JAMA Network Open.*

50-54	1.314	(1.03-1.62)	1.025	(0.94-1.13)
55-59	1.563	(1.24-2.05)	1.126	(1.01-1.21)
60-64	1.109	(0.84-1.55)	1.130	(1.04-1.22)
65-69	1.367	(1.03-1.76)	1.096	(1.00-1.21)
70-74	1.327	(0.96-1.75)	1.136	(1.04-1.24)
75-79	1.348	(0.97-1.98)	1.108	(1.03-1.19)
80-84	0.852	(0.58-1.36)	1.031	(0.93-1.14)

© 2023 Chien et al. *JAMA Network Open.*

eTable 11B. Age-specific early-stage lung ADC incidence rate ratios and their 95% confidence intervals comparing sex (1), smoking status (2), periods (3).

(1) sex

Age	Never-smoking Female/Male, 2011-2019		Ever-smoking Female/Male, 2011-2019	
	IRR	95% CI	IRR	95% CI
40-44	1.747	(1.46-2.14)	3.141	(1.78-4.71)
45-49	1.638	(1.42-1.95)	2.330	(1.63-3.57)
50-54	1.608	(1.44-1.85)	2.667	(2.05-3.38)
55-59	1.590	(1.39-1.77)	2.180	(1.65-2.68)
60-64	1.546	(1.40-1.69)	1.684	(1.31-2.18)
65-69	1.433	(1.27-1.57)	2.402	(1.89-3.05)
70-74	1.230	(1.12-1.38)	1.612	(1.17-2.12)
75-79	1.282	(1.14-1.48)	1.438	(0.89-2.02)
80-84	1.264	(1.02-1.57)	0.976	(0.53-1.56)

Age	Never-smoking Female/Male, 2011-2015		Ever-smoking Female/Male, 2011-2015	
	IRR	95% CI	IRR	95% CI
40-44	1.644	(1.18-2.48)	2.064	(0.96-3.99)
45-49	1.467	(1.15-1.89)	1.958	(1.01-3.01)
50-54	1.580	(1.25-1.99)	2.431	(1.59-3.54)
55-59	1.389	(1.16-1.65)	1.844	(1.08-2.58)
60-64	1.523	(1.29-1.73)	1.499	(1.03-2.01)
65-69	1.547	(1.18-1.94)	2.699	(1.71-3.79)

© 2023 Chien et al. *JAMA Network Open.*

70-74	1.237	(1.03-1.52)	1.760	(0.97-2.57)
75-79	1.206	(0.98-1.45)	0.634	(0.27-1.30)
80-84	0.971	(0.71-1.32)	1.199	(0.22-2.04)

Age	Never-smoking Female/Male, 2016-2019		Ever-smoking Female/Male, 2016-2019	
	**IRR**	**95% CI**	**IRR**	**95% confidence interval**
40-44	1.917	(1.48-2.52)	3.712	(2.30-6.70)
45-49	1.831	(1.48-2.19)	2.483	(1.58-3.72)
50-54	1.635	(1.41-1.95)	2.862	(2.10-3.86)
55-59	1.760	(1.54-1.96)	2.499	(1.80-3.35)
60-64	1.577	(1.39-1.78)	1.815	(1.25-2.56)
65-69	1.413	(1.27-1.57)	2.238	(1.40-3.19)
70-74	1.259	(1.07-1.44)	1.480	(0.73-2.30)
75-79	1.368	(1.13-1.70)	2.228	(1.28-3.05)
80-84	1.598	(1.23-2.22)	0.739	(0.27-1.63)

(2) smoking status

Age	Ever/never smoking Female, 2011-2019		Ever/never smoking Male, 2011-2019	
	**IRR**	**95% CI**	**IRR**	**95% CI**
40-44	0.936	(0.62-1.30)	0.521	(0.39-0.68)
45-49	1.014	(0.68-1.38)	0.712	(0.60-0.88)
50-54	1.207	(0.94-1.49)	0.728	(0.64-0.86)
55-59	1.233	(1.00-1.53)	0.899	(0.78-1.01)

© 2023 Chien et al. *JAMA Network Open.*

60-64	1.096	(0.89-1.41)	1.006	(0.89-1.14)
65-69	1.673	(1.19-2.14)	0.998	(0.87-1.14)
70-74	1.515	(1.02-2.02)	1.157	(1.00-1.36)
75-79	1.479	(1.07-2.03)	1.319	(1.13-1.45)
80-84	1.397	(0.73-2.59)	1.809	(1.34-2.26)

**Age**	**Ever/never smoking Female, 2011-2015**		**Ever/never smoking Male, 2011-2015**	
	**IRR**	**95% CI**	**IRR**	**95% CI**
40-44	0.758	(0.29-1.20)	0.604	(0.36-1.04)
45-49	0.803	(0.26-1.23)	0.602	(0.43-0.80)
50-54	1.206	(0.84-1.68)	0.784	(0.62-1.03)
55-59	1.109	(0.67-1.61)	0.835	(0.68-1.02)
60-64	1.030	(0.61-1.37)	1.047	(0.86-1.21)
65-69	2.069	(1.33-2.93)	1.186	(0.97-1.40)
70-74	1.652	(0.78-2.66)	1.161	(0.96-1.37)
75-79	0.650	(0.20-1.20)	1.237	(0.99-1.62)
80-84	1.798	(0.56-2.98)	1.456	(1.08-2.01)

**Age**	**Ever/never smoking Female, 2016-2019**		**Ever/never smoking Male, 2016-2019**	
	**IRR**	**95% CI**	**IRR**	**95% CI**
40-44	1.032	(0.69-1.56)	0.533	(0.33-0.76)
45-49	1.168	(0.79-1.62)	0.861	(0.64-1.13)
50-54	1.220	(0.86-1.64)	0.697	(0.59-0.87)
55-59	1.377	(1.02-1.71)	0.970	(0.83-1.17)

© 2023 Chien et al. *JAMA Network Open.*

60-64	1.148	(0.82-1.53)	0.998	(0.87-1.21)
65-69	1.503	(1.06-2.19)	0.949	(0.82-1.09)
70-74	1.446	(0.82-2.34)	1.230	(1.04-1.47)
75-79	2.389	(1.40-3.32)	1.467	(1.14-1.88)
80-84	1.115	(0.19-2.26)	2.412	(1.68-3.80)

(3) periods

Age	Never-smoking Female, 2016-2019/2011-2015		Never-smoking Male, 2016-2019/2011-2015	
	**IRR**	**95% CI**	**IRR**	**95% CI**
40-44	2.357	(2.01-2.96)	2.021	(1.44-3.31)
45-49	1.710	(1.52-2.01)	1.369	(1.01-1.87)
50-54	1.907	(1.70-2.19)	1.843	(1.52-2.25)
55-59	1.920	(1.72-2.16)	1.515	(1.28-1.84)
60-64	1.558	(1.43-1.73)	1.504	(1.25-1.76)
65-69	1.643	(1.46-1.82)	1.798	(1.53-2.19)
70-74	1.448	(1.26-1.68)	1.422	(1.11-1.72)
75-79	1.434	(1.26-1.66)	1.264	(0.98-1.72)
80-84	1.558	(1.24-1.85)	0.946	(0.66-1.32)
**Age**	**Ever-smoking Female, 2016-2019/2011-2015**		**Ever-smoking Male, 2016-2019/2011-2015**	
	**IRR**	**95% CI**	**IRR**	**95% CI**
40-44	3.209	(1.88-8.50)	1.784	(1.11-2.64)
45-49	2.485	(1.36-5.59)	1.960	(1.46-2.63)

© 2023 Chien et al. *JAMA Network Open.*

50-54	1.930	(1.27-3.27)	1.639	(1.26-2.05)
55-59	2.383	(1.49-3.73)	1.759	(1.52-2.09)
60-64	1.737	(1.03-2.74)	1.434	(1.23-1.66)
65-69	1.193	(0.72-1.89)	1.439	(1.16-1.70)
70-74	1.267	(0.71-2.42)	1.507	(1.25-1.92)
75-79	5.266	(2.41-22.68)	1.499	(1.16-1.85)
80-84	0.966	(0.17-7.04)	1.567	(1.11-1.98)

© 2023 Chien et al. *JAMA Network Open.*

eTable 11C. Age-specific late-stage 3&4 lung ADC incidence rate ratios and their 95% confidence intervals comparing sex (1), smoking status (2), periods (3).

(1) Sex

Age	Never-smoking Female/Male, 2011-2019		Ever-smoking Female/Male, 2011-2019	
	IRR	95% CI	IRR	95% CI
40-44	1.354	(1.17-1.62)	1.358	(0.99-1.75)
45-49	1.530	(1.34-1.80)	1.502	(1.25-1.86)
50-54	1.452	(1.31-1.66)	2.078	(1.78-2.48)
55-59	1.459	(1.35-1.60)	1.343	(1.16-1.53)
60-64	1.417	(1.33-1.53)	1.447	(1.25-1.67)
65-69	1.246	(1.14-1.33)	1.780	(1.51-2.09)
70-74	1.380	(1.28-1.50)	1.278	(1.06-1.48)
75-79	1.250	(1.14-1.33)	1.255	(1.07-1.47)
80-84	1.318	(1.23-1.45)	1.100	(0.91-1.35)

Age	Never-smoking Female/Male, 2011-2015		Ever-smoking Female/Male, 2011-2015	
	IRR	95% CI	IRR	95% CI
40-44	1.251	(1.00-1.61)	1.196	(0.85-1.60)
45-49	1.635	(1.39-2.00)	1.432	(1.10-1.78)
50-54	1.519	(1.34-1.76)	1.866	(1.43-2.32)
55-59	1.391	(1.24-1.55)	1.157	(0.93-1.38)
60-64	1.487	(1.35-1.66)	1.460	(1.16-1.78)
65-69	1.256	(1.10-1.44)	1.612	(1.20-2.00)

© 2023 Chien et al. *JAMA Network Open.*

70-74	1.254	(1.11-1.37)	1.200	(0.97-1.44)
75-79	1.236	(1.13-1.41)	1.159	(0.93-1.44)
80-84	1.251	(1.10-1.39)	1.161	(0.90-1.42)

Age	Never-smoking Female/Male, 2016-2019		Ever-smoking Female/Male, 2016-2019	
	IRR	95% CI	IRR	95% CI
40-44	1.464	(1.09-2.13)	1.570	(1.03-2.14)
45-49	1.409	(1.21-1.68)	1.563	(1.21-2.13)
50-54	1.369	(1.18-1.62)	2.345	(1.96-2.76)
55-59	1.546	(1.38-1.72)	1.578	(1.23-1.97)
60-64	1.343	(1.21-1.49)	1.433	(1.19-1.73)
65-69	1.237	(1.08-1.35)	1.962	(1.48-2.38)
70-74	1.539	(1.35-1.79)	1.398	(1.10-1.75)
75-79	1.262	(1.14-1.38)	1.384	(1.07-1.65)
80-84	1.406	(1.23-1.61)	1.014	(0.75-1.35)

(2) smoking status

Age	Ever/Never smoking Female, 2011-2019		Ever/Never smoking Male, 2011-2019	
	IRR	95% CI	IRR	95% CI
40-44	2.156	(1.49-2.66)	2.148	(1.77-2.62)
45-49	2.042	(1.72-2.39)	2.080	(1.87-2.39)
50-54	2.744	(2.40-3.17)	1.917	(1.70-2.13)
55-59	2.073	(1.73-2.45)	2.253	(2.10-2.47)

© 2023 Chien et al. *JAMA Network Open.*

60-64	2.083	(1.74-2.35)	2.040	(1.93-2.22)
65-69	2.868	(2.38-3.42)	2.007	(1.91-2.18)
70-74	2.209	(1.83-2.66)	2.385	(2.20-2.66)
75-79	2.200	(1.74-2.64)	2.191	(2.02-2.35)
80-84	1.931	(1.61-2.39)	2.313	(2.12-2.50)
**Age**	**Ever/Never smoking Female, 2011-2015**		**Ever/Never smoking Male, 2011-2015**	
	**IRR**	**95% CI**	**IRR**	**95% CI**
40-44	1.823	(1.40-2.41)	1.906	(1.49-2.38)
45-49	1.760	(1.44-2.25)	2.010	(1.71-2.48)
50-54	2.317	(1.87-2.74)	1.886	(1.62-2.22)
55-59	1.635	(1.23-2.11)	1.966	(1.77-2.19)
60-64	1.880	(1.42-2.21)	1.915	(1.74-2.07)
65-69	2.436	(1.94-2.92)	1.898	(1.65-2.17)
70-74	1.966	(1.55-2.36)	2.054	(1.88-2.32)
75-79	1.921	(1.48-2.33)	2.048	(1.82-2.31)
80-84	2.036	(1.63-2.78)	2.192	(1.93-2.45)
**Age**	**Ever/Never smoking Female, 2016-2019**		**Ever/Never smoking Male, 2016-2019**	
	**IRR**	**95% CI**	**IRR**	**95% CI**
40-44	2.615	(1.68-3.61)	2.438	(1.92-3.28)
45-49	2.437	(1.90-3.02)	2.196	(1.83-2.67)
50-54	3.350	(2.67-3.93)	1.955	(1.71-2.36)
55-59	2.702	(2.04-3.29)	2.647	(2.33-3.07)

© 2023 Chien et al. *JAMA Network Open.*

60-64	2.324	(1.77-2.82)	2.178	(1.95-2.45)
65-69	3.343	(2.81-4.01)	2.108	(1.91-2.39)
70-74	2.603	(1.91-3.11)	2.866	(2.50-3.17)
75-79	2.605	(1.99-3.33)	2.376	(2.14-2.74)
80-84	1.786	(1.39-2.44)	2.477	(2.18-2.78)

(3) periods

Age	Never-smoking Female, 2016-2019/2011-2015		Never-smoking Male, 2016-2019/2011-2015	
	**IRR**	**95% CI**	**IRR**	**95% CI**
40-44	0.882	(0.73-1.05)	0.754	(0.55-1.07)
45-49	0.914	(0.80-1.05)	1.060	(0.82-1.30)
50-54	0.902	(0.83-0.99)	1.001	(0.79-1.22)
55-59	0.932	(0.86-1.02)	0.839	(0.71-0.98)
60-64	0.889	(0.82-0.95)	0.984	(0.87-1.13)
65-69	0.975	(0.90-1.06)	0.989	(0.88-1.11)
70-74	1.010	(0.95-1.09)	0.822	(0.72-0.94)
75-79	0.978	(0.91-1.05)	0.958	(0.85-1.10)
80-84	1.008	(0.94-1.09)	0.897	(0.77-1.02)
**Age**	**Ever-smoking Female, 2016-2019/2011-2015**		**Ever-smoking Male, 2016-2019/2011-2015**	
	**IRR**	**95% CI**	**IRR**	**95% CI**
40-44	1.265	(0.82-1.92)	0.964	(0.77-1.21)
45-49	1.265	(0.92-1.77)	1.158	(1.00-1.34)

© 2023 Chien et al. *JAMA Network Open.*

50-54	1.304	(1.03-1.60)	1.037	(0.95-1.14)
55-59	1.540	(1.20-2.11)	1.129	(1.03-1.22)
60-64	1.098	(0.79-1.48)	1.119	(1.03-1.22)
65-69	1.338	(1.00-1.74)	1.099	(1.00-1.21)
70-74	1.336	(0.97-1.72)	1.147	(1.04-1.26)
75-79	1.326	(0.98-1.91)	1.111	(1.02-1.20)
80-84	0.884	(0.60-1.30)	1.013	(0.92-1.10)

© 2023 Chien et al. *JAMA Network Open.*

eTable 12A. Age-specific numbers of never- and ever-smoking males in the Taiwan Biobank; among the ever-smokers, the estimated number of pack-years smoked, number of cigarettes smoked per day (intensity), number of years smoked (duration), age at smoking initiation, and years since cessation based on the Taiwan Biobank.

Male

	N	nonsmoker	ever-smokers	N_SmkPY1	mean	sd	N_Smoking intensity	mean	sd	N_Smoking duration	mean	sd
30-34	5476	3817	1659	1618	7.87	8.30	1629	13.44	11.00	1646	11.03	5.61
35-39	6237	4026	2210	2158	10.88	10.07	2179	14.51	11.24	2186	14.53	7.14
40-44	5877	3312	2563	2497	13.67	12.38	2532	16.15	12.14	2521	17.28	8.74
45-49	5656	2823	2833	2763	15.95	15.16	2807	16.91	12.70	2783	19.07	10.60
50-54	5922	2796	3126	3022	17.97	16.91	3073	17.55	13.16	3068	21.21	12.44
55-59	6710	3248	3459	3340	19.41	19.18	3412	18.03	13.71	3379	22.16	14.11
60-64	6630	3374	3253	3123	19.53	20.65	3196	17.87	14.29	3167	23.01	15.52
65-70	5486	3115	2370	2248	19.67	20.94	2338	17.65	14.71	2265	24.48	17.26

	N_Smoking initiation	Mean	Sd	current	former	N_QuitYears	mean	sd
30-34	1629	18.29	3.76	1139	520	519	5.26	4.43
35-39	2169	18.56	4.02	1466	743	740	7.31	5.66
40-44	2499	18.81	4.16	1532	1031	1028	8.76	6.73
45-49	2776	19.31	4.21	1428	1405	1400	11.12	8.09

© 2023 Chien et al. *JAMA Network Open.*

	N_Smoking initiation	Mean	Sd	current	former	N_QuitYears	mean	sd
50-54	3053	19.77	4.42	1421	1705	1692	13.01	9.36
55-59	3387	20.63	4.77	1291	2168	2150	15.18	10.59
60-64	3160	21.51	5.40	988	2265	2234	17.18	11.73
65-70	2294	22.41	6.22	642	1728	1693	18.86	12.76

© 2023 Chien et al. *JAMA Network Open.*

eTable 12B. Age-specific numbers of never- and ever-smoking females in the Taiwan Biobank; among the ever-smokers, the estimated number of pack-years smoked, number of cigarettes smoked per day (intensity), number of years smoked (duration), age at smoking initiation, and years since cessation based on the Taiwan Biobank.

Female

	N	nonsmoker	ever-smokers	N_SmkPY1	mean	sd	N_Smoking intensity	mean	sd	N_Smoking duration	mean	sd
30-34	8533	7729	800	758	4.38	5.27	776	8.98	8.05	780	9.12	5.58
35-39	10314	9370	942	903	5.41	6.20	918	8.98	8.17	921	11.49	7.23
40-44	10381	9504	872	822	7.33	9.18	851	9.93	9.20	837	14.30	8.81
45-49	10287	9542	740	685	9.03	11.71	715	10.12	9.78	700	16.62	10.64
50-54	12730	12036	679	627	9.83	11.43	653	10.47	9.55	645	17.78	11.94
55-59	13856	13390	457	419	11.37	13.74	445	10.67	9.99	427	20.23	13.26
60-64	11498	11211	284	260	12.42	13.43	275	11.21	10.21	265	22.24	14.18
65-70	7126	7005	120	106	11.73	14.17	115	11.70	10.99	110	21.07	16.98

	N_Smoking initiation	mean	sd	current	former	N_QuitYears	mean	sd
30-34	772	18.69	3.82	438	362	358	5.87	4.44
35-39	915	19.41	4.51	507	435	434	8.25	5.52
40-44	840	20.08	5.38	482	390	385	10.52	7.15
45-49	706	21.68	6.34	414	325	317	12.58	8.30
50-54	650	24.10	7.43	344	335	325	14.56	10.17

© 2023 Chien et al. *JAMA Network Open.*

	N_Smoking initiation	mean	sd	current	former	N_QuitYears	mean	sd
55-59	424	25.17	8.44	207	250	242	13.69	10.78
60-64	264	28.54	9.68	144	140	134	15.46	11.60
65-70	110	30.82	10.70	48	72	69	18.35	12.19

© 2023 Chien et al. *JAMA Network Open.*

eTable 13 Histology codes for lung ADC that appeared in the TCR for each year from 2011 to 2019.

2011	2012	2013	2014	2015	2016	2017	2018	2019
81403	80503	80503	80503	80503	80503	80503	80503	81403
81413	81403	81403	81403	81403	81403	81403	81403	81443
81433	82303	81413	81413	81433	81443	81413	81443	82013
82503	82503	82303	82303	82013	82013	81443	82303	82303
82553	82513	82503	82503	82303	82133	82013	82503	82503
82603	82523	82513	82513	82503	82303	82113	82513	82523
83103	82533	82523	82523	82513	82503	82303	82533	82533
84803	82543	82533	82533	82523	82513	82503	82543	82543
84813	82553	82543	82543	82533	82533	82513	82553	82553
84903	82603	82553	82553	82543	82543	82523	82563	82563
85003	83103	82603	82563	82553	82553	82533	82573	82573
85713	83233	84803	82603	82563	82563	82543	82603	82603
	83333	84813	82653	82573	82573	82553	82653	82653
	84803	84903	83103	82603	82603	82563	83203	83333
	84813	85033	83203	82653	82623	82573	83333	84803
	84903	85503	84803	83103	82653	82603	84803	84813
	85503		84813	83233	83103	82653	84813	84903
			84903	84803	83333	83103	84903	85513
			85503	84813	84803	83333	85513	
			85723	84903	84813	84803		
				85503	84903	84813		
				85513	85503	84903		
					85513	85503		
					85723	85523		

© 2023 Chien et al. *JAMA Network Open.*

eTable 14. Age- and sex-specific number of patient with invasive lung SCC diagnosed for each year from 2011 to 2019 according to the TCR.

**Year**	**2011-2015**		**2016-2019**	
Sex / Age	M	F	M	F
40-44	57	22	36	11
45-49	141	36	106	26
50-54	324	61	250	57
55-59	528	90	422	67
60-64	798	124	698	108
65-69	899	115	899	109
70-74	1242	141	792	92
75-79	1301	136	903	110
80-84	1147	120	751	86

© 2023 Chien et al. *JAMA Network Open.*

eTable 15A. Age-, sex-, and calendar year-specific percentages of patients with late-stage (stage2—4) SCC among those with all stage (1—4) invasive SCC, estimated using the TCRLF for each year from 2011 to 2019.

**Year**	**2011-2015**		**2016-2019**	
Sex / Age	M	F	M	F
40-44	0.882	0.955	0.971	1.000
45-49	0.899	0.879	0.921	1.000
50-54	0.923	0.929	0.933	0.923
55-59	0.914	0.966	0.887	0.968
60-64	0.898	0.849	0.906	0.933
65-69	0.878	0.907	0.863	0.886
70-74	0.866	0.898	0.850	0.908
75-79	0.883	0.895	0.887	0.894
80-84	0.885	0.855	0.893	0.951

© 2023 Chien et al. *JAMA Network Open.*

eTable 15B. Age-, sex- and calendar year-specific percentages of ever-smokers among corresponding patients with late-stage (2—4) SCC in Taiwan.

Year	2011-2015		2016-2019	
Sex / Age	M	F	M	F
40-44	0.841	0.105	0.750	0.400
45-49	0.852	0.103	0.874	0.261
50-54	0.920	0.159	0.901	0.234
55-59	0.875	0.210	0.892	0.200
60-64	0.892	0.175	0.879	0.163
65-69	0.875	0.193	0.863	0.176
70-74	0.883	0.205	0.879	0.233
75-79	0.860	0.223	0.863	0.167
80-84	0.862	0.325	0.824	0.250

© 2023 Chien et al. *JAMA Network Open.*

eTable 15C. Age-, sex- and year-specific percentages of ever-smokers among corresponding patients with early-stage (1) SCC in Taiwan.

Year	2011-2015		2016-2019	
Sex / Age	M	F	M	F
40-64	0.909	0.133	0.925	0.538
65-84	0.872	0.333	0.910	0.563

© 2023 Chien et al. *JAMA Network Open.*

eTable 15D. Age-, sex-, and calendar year-specific percentages of patients with stage 3&4 SCC among patients with all stages (1—4) of invasive SCC, estimated using the TCRLF for each year from 2011 to 2019.

**Year**	**2011-2015**		**2016-2019**	
Sex / Age	M	F	M	F
40-44	0.745	0.955	0.886	0.909
45-49	0.841	0.879	0.832	1.000
50-54	0.816	0.929	0.837	0.846
55-59	0.802	0.909	0.814	0.952
60-64	0.813	0.807	0.819	0.905
65-69	0.789	0.832	0.768	0.838
70-74	0.758	0.839	0.754	0.862
75-79	0.782	0.847	0.807	0.846
80-84	0.806	0.809	0.804	0.902

© 2023 Chien et al. *JAMA Network Open.*

eTable 15E. Age-, sex- and year-specific percentages of ever-smokers among corresponding patients with stage 3&4 SCC in Taiwan.

Year	2011-2015		2016-2019	
Age \ Sex	M	F	M	F
40-44	0.892	0.105	0.759	0.444
45-49	0.852	0.103	0.859	0.261
50-54	0.914	0.159	0.889	0.233
55-59	0.878	0.171	0.891	0.203
60-64	0.890	0.152	0.876	0.169
65-69	0.866	0.160	0.855	0.163
70-74	0.884	0.190	0.883	0.246
75-79	0.856	0.196	0.866	0.163
80-84	0.868	0.311	0.815	0.250

© 2023 Chien et al. *JAMA Network Open.*

eTable 16A. Estimated age- and sex-specific number of patients with late-stage lung SCC in the TCR by smoking status.

	Never-smokers			
Year	2011-2015		2016-2019	
Age \ Sex	M	F	M	F
40-44	8.001	18.789	8.743	6.600
45-49	18.693	28.364	12.341	19.217
50-54	23.913	47.631	22.998	40.301
55-59	60.350	68.687	40.389	51.898
60-64	77.670	86.799	76.258	84.365
65-69	98.936	84.113	106.340	79.506
70-74	125.777	100.595	81.255	64.086
75-79	160.398	94.557	109.390	81.971
80-84	139.949	69.251	118.319	61.354

	Ever-smokers			
Year	2011-2015		2016-2019	
Age \ Sex	M	F	M	F
40-44	42.293	2.211	26.229	4.400
45-49	108.003	3.273	85.263	6.783
50-54	275.003	9.011	210.266	12.314
55-59	422.451	18.245	334.032	12.975
60-64	638.859	18.445	555.912	16.435
65-69	690.327	20.140	669.834	17.037
70-74	949.561	25.996	591.998	19.455
75-79	988.926	27.185	691.625	16.394
80-84	874.998	33.294	552.560	20.451

© 2023 Chien et al. *JAMA Network Open.*

eTable 16B. Estimated age- and sex-specific number of patients with early-stage lung SCC in the TCR by smoking status.

	**Never-smokers**			
**Year**	**2011-2015**		**2016-2019**	
Sex / Age	M	F	M	F
40-64	15.802	27.339	10.416	6.328
65-84	71.724	37.913	38.120	16.077

	**Ever-smokers**			
**Year**	**2011-2015**		**2016-2019**	
Sex / Age	M	F	M	F
40-64	156.962	4.206	129.153	7.383
65-84	488.404	18.956	385.559	20.670

© 2023 Chien et al. *JAMA Network Open.*

eTable 16C. Estimated age- and sex-specific number of patients with stage 3&4 lung SCC in the TCR by smoking status.

	Never-smokers			
Year	2011-2015		2016-2019	
Age \ Sex	M	F	M	F
40-44	4.591	18.789	7.697	5.556
45-49	17.521	28.364	12.433	19.217
50-54	22.852	47.631	23.123	37.014
55-59	51.560	67.823	37.301	50.831
60-64	71.339	84.811	71.030	81.246
65-69	94.766	80.302	99.898	76.508
70-74	108.775	95.813	70.088	59.770
75-79	146.741	92.604	97.877	77.952
80-84	121.727	66.914	111.411	58.207

	Ever-smokers			
Year	2011-2015		2016-2019	
Age \ Sex	M	F	M	F
40-44	37.879	2.211	24.189	4.444
45-49	101.001	3.273	75.726	6.783
50-54	241.574	9.011	186.082	11.216
55-59	371.662	13.995	306.091	12.978
60-64	577.297	15.223	500.536	16.469
65-69	614.305	15.352	590.608	14.845
70-74	832.809	22.544	526.828	19.540
75-79	871.201	22.557	630.897	15.125
80-84	802.639	30.177	492.167	19.402

© 2023 Chien et al. *JAMA Network Open.*

eTable 17. Number of patients with invasive lung SCC in TCR, TCRLF, having known smoking status (ever-smoker or never-smoker), and having a known number of pack-years smoked and a known number of years since smoking quitting, for 2011—2019.

Year	TCR	TCRLF	Smoking status known	Pack-year and quit time known
2011	1735	1647	1522	928
2012	1632	1575	1412	822
2013	1552	1491	1353	830
2014	1639	1600	1413	852
2015	1639	1604	1434	885
2016	1547	1510	1353	843
2017	1545	1519	1370	836
2018	1562	1529	1375	817
2019	1557	1526	1356	828
Sum	14408	14001	12588	7641

© 2023 Chien et al. *JAMA Network Open.*

eTable 18A. Age-specific incidence rates for late-stage SCC in the periods 2011—2019, 2011—2015, and 2016--2019 by sex and smoking status: never-smoking female (1), never-smoking male (2), ever-smoking female (3), ever-smoking male (4).

(1) never-smoking female

Age	Never-smoking Female					
	2011-2019		2011-2015		2016-2019	
	rate	95% Cl	rate	95% Cl	rate	95% Cl
40-44	0.333	(0.20-0.46)	0.450	(0.24-0.62)	0.191	(0.06-0.32)
45-49	0.630	(0.46-0.80)	0.665	(0.42-0.89)	0.579	(0.33-0.84)
50-54	1.152	(0.92-1.39)	1.128	(0.83-1.47)	1.191	(0.83-1.60)
55-59	1.689	(1.39-1.99)	1.791	(1.38-2.19)	1.570	(1.12-2.00)
60-64	2.864	(2.43-3.29)	2.848	(2.20-3.41)	2.879	(2.29-3.51)
65-69	3.956	(3.39-4.59)	4.481	(3.57-5.43)	3.520	(2.75-4.25)
70-74	5.554	(4.69-6.41)	6.161	(4.96-7.29)	4.809	(3.68-6.08)
75-79	7.334	(6.23-8.39)	7.503	(5.95-9.05)	7.146	(5.58-8.63)
80-84	7.966	(6.67-9.28)	8.125	(6.22-9.97)	7.710	(5.78-9.80)

(2) never-smoking male

Age	Never-smoking Male					
	2011-2019		2011-2015		2016-2019	
	rate	95% Cl	rate	95% Cl	rate	95% Cl
40-44	0.441	(0.21-0.63)	0.426	(0.16-0.74)	0.449	(0.15-0.72)
45-49	0.885	(0.60-1.22)	1.002	(0.59-1.39)	0.747	(0.36-1.15)
50-54	1.459	(1.03-1.87)	1.351	(0.79-1.92)	1.592	(0.90-2.21)
55-59	3.089	(2.48-3.68)	3.533	(2.69-4.45)	2.598	(1.80-3.41)
60-64	5.484	(4.60-6.38)	5.586	(4.24-6.83)	5.386	(4.24-6.50)
65-69	11.134	(9.65-12.63)	12.617	(10.07-15.05)	10.033	(8.11-11.89)
70-74	14.890	(12.73-16.83)	17.831	(14.89-21.12)	11.865	(9.35-14.45)
75-79	26.778	(23.52-29.87)	32.549	(27.80-37.54)	21.248	(17.29-25.25)
80-84	34.683	(30.29-38.87)	34.822	(28.86-40.31)	34.369	(28.47-40.09)

(3) ever-smoking female

© 2023 Chien et al. *JAMA Network Open.*

Age	Ever-smoking Female					
	2011-2019		2011-2015		2016-2019	
	**rate**	**95% Cl**	**rate**	**95% Cl**	**rate**	**95% Cl**
40-44	1.089	(0.33-1.81)	0.661	(0.00-1.49)	1.608	(0.37-2.92)
45-49	1.927	(0.77-2.89)	1.127	(0.00-2.41)	2.976	(0.44-5.25)
50-54	5.455	(3.28-7.57)	4.027	(1.79-7.15)	7.142	(3.48-11.02)
55-59	11.215	(7.54-15.45)	11.348	(6.22-16.17)	11.032	(5.10-16.15)
60-64	16.802	(11.56-22.15)	17.150	(9.30-25.11)	16.411	(8.99-23.97)
65-69	37.752	(26.43-49.82)	39.133	(23.32-56.35)	36.329	(19.19-53.31)
70-74	62.426	(43.97-78.32)	57.753	(35.55-75.54)	70.077	(39.62-100.86)
75-79	74.321	(52.90-97.28)	78.468	(46.18-109.69)	68.445	(37.58-96.03)
80-84	133.101	(97.53-169.95)	142.145	(93.93-196.40)	123.275	(72.34-174.81)

(4) ever-smoking male

Age	Ever-smoking Male					
	2011-2019		2011-2015		2016-2019	
	**rate**	**95% Cl**	**rate**	**95% Cl**	**rate**	**95% Cl**
40-44	1.598	(1.21-1.96)	1.630	(1.19-2.12)	1.546	(0.94-2.18)
45-49	4.228	(3.61-4.82)	3.974	(3.20-4.75)	4.607	(3.57-5.57)
50-54	10.177	(9.27-11.18)	10.278	(9.04-11.47)	10.047	(8.74-11.42)
55-59	18.852	(17.52-20.23)	19.105	(17.14-20.80)	18.541	(16.76-20.48)
60-64	38.528	(36.31-40.76)	39.250	(36.13-42.45)	37.728	(34.48-40.99)
65-69	66.721	(62.90-70.25)	70.233	(65.11-75.79)	63.468	(58.84-68.41)
70-74	127.936	(121.58-134.11)	131.647	(122.83-140.16)	122.396	(112.27-132.52)
75-79	181.130	(172.56-189.80)	184.047	(172.15-195.60)	177.122	(163.90-191.30)
80-84	232.683	(220.93-244.89)	227.286	(212.48-241.83)	241.977	(222.04-262.74)

© 2023 Chien et al. *JAMA Network Open.*

eTable 18B. Age-specific incidence rates for early-stage SCC in the periods 2011—2019, 2011—2015, and 2016—2019 by sex and smoking status: never-smoking female (1), never-smoking male (2), ever-smoking female (3), ever-smoking male (4).

(1) never-smoking female

Never-smoking Female							
**Age**	2011-2019		**Age**	2011-2015		2016-2019	
	**rate**	**95% Cl**		**rate**	**95% Cl**	**rate**	**95% Cl**
40-44	0.013	(0.00-0.07)*	40-64	0.140	(0.09-0.19)	0.039	(0.01-0.08)*
45-49	0.056	(0.01-0.11)	65-84	0.674	(0.46-0.87)	0.291	(0.16-0.45)
50-54	0.086	(0.03-0.14)					
55-59	0.044	(0.00-0.10)					
60-64	0.312	(0.17-0.43)					
65-69	0.355	(0.19-0.53)					
70-74	0.478	(0.24-0.74)					
75-79	0.468	(0.21-0.75)					
80-84	0.842	(0.42-1.27)					

*Use the exact binomial 95% confidence interval.

(2) never-smoking male

Never-smoking Male							
**Age**	2011-2019		**Age**	2011-2015		2016-2019	
	**rate**	**95% Cl**		**rate**	**95% Cl**	**rate**	**95% Cl**
40-44	0.000	(0.00-0.00)	40-64	0.183	(0.09-0.27)	0.130	(0.05-0.21)
45-49	0.059	(0.00-0.14)	65-84	3.008	(2.31-3.69)	1.464	(1.00-1.88)
50-54	0.133	(0.03-0.25)					
55-59	0.316	(0.15-0.52)					
60-64	0.335	(0.14-0.53)					
65-69	1.168	(0.70-1.68)					
70-74	2.053	(1.29-2.81)					
75-79	2.662	(1.59-3.57)					
80-84	4.471	(3.08-6.03)					

(3) ever-smoking female

© 2023 Chien et al. *JAMA Network Open.*

Ever-smoking Female							
Age	2011-2019		Age	2011-2015		2016-2019	
	**rate**	**95% Cl**		**rate**	**95% Cl**	**rate**	**95% Cl**
40-44	0.000	(0.00-0.00)	40-64	0.377	(0.09-0.72)	0.828	(0.22-1.46)
45-49	0.000	(0.00-0.00)	65-84	12.266	(6.47-17.47)	17.943	(10.42-25.17)
50-54	0.552	(0.00-1.26)					
55-59	0.747	(0.00-1.80)					
60-64	3.491	(1.44-6.26)					
65-69	8.707	(3.05-14.23)					
70-74	11.979	(4.12-19.24)					
75-79	24.978	(11.95-35.84)					
80-84	18.928	(5.00-32.49)					

(4) ever-smoking male

Ever-smoking Male							
Age	2011-2019		Age	2011-2015		2016-2019	
	**rate**	**95% Cl**		**rate**	**95% Cl**	**rate**	**95% Cl**
40-44	0.176	(0.05-0.28)	40-64	1.327	(1.12-1.54)	1.449	(1.20-1.70)
45-49	0.452	(0.26-0.63)	65-84	18.595	(16.90-20.29)	17.867	(16.27-19.70)
50-54	0.787	(0.55-1.03)					
55-59	2.054	(1.62-2.54)					
60-64	4.447	(3.68-5.22)					
65-69	10.360	(8.83-11.82)					
70-74	21.318	(18.92-23.98)					
75-79	24.449	(21.34-27.59)					
80-84	29.141	(24.95-33.26)					

© 2023 Chien et al. *JAMA Network Open.*

eTable 18C. Age-specific incidence rates for stage 3&4 SCC in the periods 2011—2019 , 2011—2015, and 2016—2019 by sex and smoking status: never-smoking female (1), never-smoking male (2), ever-smoking female (3), ever-smoking male (4).

(1) never-smoking female

Age	Never-smoking Female					
	2011-2019		2011-2015		2016-2019	
	rate	95% Cl	rate	95% Cl	rate	95% Cl
40-44	0.320	(0.20-0.45)	0.450	(0.24-0.62)	0.161	(0.03-0.26)
45-49	0.630	(0.46-0.80)	0.665	(0.42-0.89)	0.579	(0.33-0.84)
50-54	1.109	(0.88-1.35)	1.128	(0.83-1.47)	1.094	(0.74-1.48)
55-59	1.661	(1.36-1.96)	1.768	(1.36-2.16)	1.538	(1.12-1.97)
60-64	2.779	(2.34-3.19)	2.782	(2.17-3.35)	2.772	(2.18-3.41)
65-69	3.792	(3.19-4.40)	4.278	(3.36-5.22)	3.388	(2.61-4.12)
70-74	5.248	(4.38-6.04)	5.868	(4.72-6.92)	4.486	(3.30-5.55)
75-79	7.086	(6.02-8.10)	7.348	(5.79-8.89)	6.796	(5.23-8.28)
80-84	7.622	(6.31-8.86)	7.851	(5.87-9.62)	7.314	(5.40-9.30)

(2) never-smoking male

Age	Never-smoking Male					
	2011-2019		2011-2015		2016-2019	
	rate	95% Cl	rate	95% Cl	rate	95% Cl
40-44	0.325	(0.16-0.50)	0.244	(0.05-0.48)	0.395	(0.10-0.67)
45-49	0.853	(0.54-1.14)	0.939	(0.54-1.34)	0.753	(0.36-1.15)
50-54	1.429	(1.00-1.83)	1.291	(0.73-1.81)	1.600	(0.97-2.28)
55-59	2.725	(2.15-3.31)	3.019	(2.17-3.81)	2.400	(1.67-3.15)
60-64	5.071	(4.24-5.92)	5.130	(3.81-6.26)	5.017	(3.88-6.14)
65-69	10.558	(9.00-11.98)	12.085	(9.57-14.53)	9.425	(7.45-11.23)
70-74	12.864	(10.86-14.67)	15.421	(12.76-18.57)	10.234	(8.03-12.70)
75-79	24.287	(21.34-27.29)	29.778	(24.96-34.90)	19.012	(14.76-22.72)
80-84	31.335	(27.34-35.38)	30.288	(24.89-35.58)	32.362	(26.15-38.05)

(3) ever-smoking female

© 2023 Chien et al. *JAMA Network Open.*

Age	Ever-smoking Female					
	2011-2019		2011-2015		2016-2019	
	rate	95% Cl	rate	95% Cl	rate	95% Cl
40-44	1.092	(0.33-1.81)	0.661	(0.00-1.49)	1.624	(0.37-2.92)
45-49	1.927	(0.77-2.89)	1.127	(0.00-2.41)	2.976	(0.44-5.25)
50-54	5.173	(3.03-7.32)	4.027	(1.79-7.15)	6.505	(3.48-10.44)
55-59	9.683	(6.11-13.29)	8.705	(3.73-12.44)	11.035	(5.10-16.15)
60-64	15.261	(10.11-20.70)	14.154	(7.44-21.39)	16.445	(8.99-23.97)
65-69	30.705	(20.33-41.68)	29.830	(15.54-44.69)	31.654	(14.93-46.91)
70-74	57.747	(41.22-74.20)	50.084	(28.88-68.87)	70.386	(39.62-100.86)
75-79	64.247	(44.37-83.62)	65.110	(37.52-89.48)	63.146	(33.40-96.03)
80-84	123.120	(89.97-159.95)	128.837	(85.39-179.32)	116.953	(72.34-174.66)

(4) ever-smoking male

Age	Ever-smoking Male					
	2011-2019		2011-2015		2016-2019	
	rate	95% Cl	rate	95% Cl	rate	95% Cl
40-44	1.449	(1.10-1.79)	1.460	(0.96-1.93)	1.425	(0.82-2.00)
45-49	3.868	(3.31-4.49)	3.717	(2.91-4.49)	4.092	(3.13-4.97)
50-54	8.969	(8.10-9.92)	9.028	(7.89-10.13)	8.891	(7.60-10.18)
55-59	16.888	(15.58-18.17)	16.808	(15.01-18.59)	16.990	(15.27-18.82)
60-64	34.758	(32.57-36.86)	35.468	(32.56-38.52)	33.970	(31.02-36.99)
65-69	59.102	(55.78-62.26)	62.498	(57.69-67.96)	55.962	(51.55-60.45)
70-74	112.837	(106.98-118.67)	115.460	(107.17-123.52)	108.922	(99.86-118.46)
75-79	161.896	(154.24-169.86)	162.137	(151.68-172.34)	161.570	(149.30-174.40)
80-84	210.997	(199.73-223.04)	208.491	(194.56-222.87)	215.529	(197.06-235.15)

© 2023 Chien et al. *JAMA Network Open.*

eTable 19A. Age-specific late-stage lung SCC incidence rate ratios and their 95% confidence intervals comparing sex (1), smoking status (2), periods (3).

(1) sex

Age	Never-smoking Female/Male, 2011-2019		Ever-smoking Female/Male, 2011-2019	
	IRR	95% CI	IRR	95% CI
40-44	0.756	(0.39-1.79)	0.681	(0.32-1.48)
45-49	0.711	(0.48-1.22)	0.456	(0.26-0.80)
50-54	0.790	(0.56-1.40)	0.536	(0.31-0.83)
55-59	0.547	(0.42-0.71)	0.595	(0.40-0.81)
60-64	0.522	(0.42-0.65)	0.436	(0.29-0.57)
65-69	0.355	(0.28-0.46)	0.566	(0.42-0.75)
70-74	0.373	(0.31-0.47)	0.488	(0.34-0.62)
75-79	0.274	(0.22-0.34)	0.410	(0.29-0.56)
80-84	0.230	(0.16-0.27)	0.572	(0.40-0.77)

Age	Never-smoking Female/Male, 2011-2015		Ever-smoking Female/Male, 2011-2015	
	IRR	95% CI	IRR	95% CI
40-44	1.058	(0.43-3.80)	0.405	(0.00-1.14)
45-49	0.664	(0.36-1.41)	0.284	(0.00-0.67)
50-54	0.835	(0.56-1.48)	0.392	(0.20-0.65)
55-59	0.507	(0.33-0.77)	0.594	(0.36-0.87)
60-64	0.510	(0.37-0.76)	0.437	(0.22-0.59)
65-69	0.355	(0.25-0.49)	0.557	(0.33-0.87)

© 2023 Chien et al. *JAMA Network Open.*

70-74	0.345	(0.23-0.45)	0.439	(0.27-0.65)
75-79	0.231	(0.18-0.29)	0.426	(0.26-0.65)
80-84	0.233	(0.17-0.29)	0.625	(0.42-0.83)

**Age**	**Never-smoking Female/Male, 2016-2019**		**Ever-smoking Female/Male, 2016-2019**	
	**IRR**	**95% CI**	**IRR**	**95% CI**
40-44	0.426	(0.12-1.21)	1.040	(0.13-2.28)
45-49	0.775	(0.38-2.77)	0.646	(0.15-1.35)
50-54	0.748	(0.46-1.41)	0.711	(0.30-1.12)
55-59	0.604	(0.40-0.92)	0.595	(0.33-1.12)
60-64	0.534	(0.41-0.79)	0.435	(0.24-0.69)
65-69	0.351	(0.26-0.44)	0.572	(0.34-0.81)
70-74	0.405	(0.28-0.58)	0.573	(0.30-0.81)
75-79	0.336	(0.25-0.42)	0.386	(0.22-0.60)
80-84	0.224	(0.16-0.33)	0.509	(0.28-0.69)

(2) smoking status

**Age**	**Ever/never smoking Female, 2011-2019**		**Ever/never smoking Male, 2011-2019**	
	**IRR**	**95% CI**	**IRR**	**95% CI**
40-44	3.270	(0.90-8.16)	3.626	(2.47-6.50)
45-49	3.061	(1.32-5.66)	4.778	(3.44-7.46)
50-54	4.734	(2.84-7.15)	6.976	(5.21-10.91)
55-59	6.642	(3.93-9.56)	6.102	(5.09-8.02)

© 2023 Chien et al. *JAMA Network Open.*

60-64	5.868	(3.78-8.36)	7.026	(5.84-8.37)
65-69	9.542	(6.75-14.16)	5.993	(5.23-6.96)
70-74	11.241	(7.78-15.51)	8.592	(7.45-10.19)
75-79	10.134	(7.13-13.63)	6.764	(6.03-7.63)
80-84	16.709	(11.72-24.79)	6.709	(5.81-7.58)

Age	Ever/never smoking Female, 2011-2015		Ever/never smoking Male, 2011-2015	
	IRR	95% CI	IRR	95% CI
40-44	1.467	(0.00-3.83)	3.830	(1.78-11.50)
45-49	1.694	(0.00-4.56)	3.965	(2.57-9.05)
50-54	3.569	(1.78-6.85)	7.606	(4.52-12.13)
55-59	6.337	(3.34-10.94)	5.407	(4.32-6.87)
60-64	6.023	(3.15-9.73)	7.027	(5.65-9.27)
65-69	8.733	(4.74-13.47)	5.567	(4.75-6.66)
70-74	9.374	(5.61-13.75)	7.383	(6.40-8.97)
75-79	10.459	(6.51-14.42)	5.654	(4.87-6.90)
80-84	17.495	(11.66-26.19)	6.527	(5.40-7.83)

Age	Ever/never smoking Female, 2016-2019		Ever/never smoking Male, 2016-2019	
	IRR	95% CI	IRR	95% CI
40-44	8.411	(1.63-25.24)	3.441	(1.57-15.75)
45-49	5.138	(1.38-11.75)	6.166	(3.33-15.05)
50-54	5.998	(2.44-10.80)	6.312	(4.50-9.71)
55-59	7.026	(3.38-11.66)	7.136	(5.39-10.31)

© 2023 Chien et al. *JAMA Network Open.*

60-64	5.701	(2.97-8.81)	7.005	(5.55-9.62)
65-69	10.320	(5.70-17.34)	6.326	(5.28-7.94)
70-74	14.571	(8.03-22.95)	10.316	(7.95-13.33)
75-79	9.578	(5.25-15.34)	8.336	(6.70-10.63)
80-84	15.990	(8.77-24.42)	7.041	(5.60-8.77)

(3) periods

**Age**	**Never-smoking Female, 2016-2019/2011-2015**		**Never-smoking Male, 2016-2019/2011-2015**	
	**IRR**	**95% CI**	**IRR**	**95% CI**
40-44	0.424	(0.12-1.15)	1.055	(0.35-5.26)
45-49	0.871	(0.43-1.55)	0.745	(0.33-1.41)
50-54	1.055	(0.67-1.68)	1.178	(0.63-2.05)
55-59	0.877	(0.61-1.22)	0.735	(0.46-1.18)
60-64	1.011	(0.73-1.37)	0.964	(0.69-1.26)
65-69	0.786	(0.58-1.10)	0.795	(0.63-1.01)
70-74	0.781	(0.57-1.07)	0.665	(0.50-0.83)
75-79	0.952	(0.67-1.29)	0.653	(0.52-0.88)
80-84	0.949	(0.66-1.37)	0.987	(0.77-1.31)
**Age**	**Ever-smoking Female, 2016-2019/2011-2015**		**Ever-smoking Male, 2016-2019/2011-2015**	
	**IRR**	**95% CI**	**IRR**	**95% CI**
40-44	2.434	(0.44-8.56)	0.948	(0.46-1.48)
45-49	2.641	(0.96-13.40)	1.159	(0.88-1.51)

© 2023 Chien et al. *JAMA Network Open.*

50-54	1.773	(0.69-4.49)	0.978	(0.81-1.15)
55-59	0.972	(0.51-2.39)	0.970	(0.82-1.16)
60-64	0.957	(0.48-1.83)	0.961	(0.85-1.07)
65-69	0.928	(0.49-1.87)	0.904	(0.81-1.01)
70-74	1.213	(0.71-2.40)	0.930	(0.83-1.04)
75-79	0.872	(0.38-1.59)	0.962	(0.86-1.07)
80-84	0.867	(0.50-1.39)	1.065	(0.96-1.17)

© 2023 Chien et al. *JAMA Network Open.*

eTable 19B. Age-specific early-stage lung SCC incidence rate ratios and their 95% confidence intervals comparing sex (1), smoking status (2), periods (3).

(1) sex

Age	Never-smoking Female/Male, 2011-2019		Ever-smoking Female/Male, 2011-2019	
	**IRR**	**95% CI**	**IRR**	**95% CI**
40-44	Inf	NA	0.000	(0.00-0.00)
45-49	0.960	(0.01-2.78)	0.000	(0.00-0.00)
50-54	0.646	(0.19-3.60)	0.701	(0.00-1.78)
55-59	0.138	(0.00-0.46)	0.364	(0.00-0.84)
60-64	0.931	(0.43-2.32)	0.785	(0.26-1.33)
65-69	0.304	(0.18-0.58)	0.840	(0.41-1.56)
70-74	0.233	(0.12-0.54)	0.562	(0.23-1.07)
75-79	0.176	(0.07-0.40)	1.022	(0.62-1.71)
80-84	0.188	(0.10-0.40)	0.650	(0.32-1.27)
**Age**	**Never-smoking Female/Male, 2011-2015**		**Ever-smoking Female/Male, 2011-2015**	
	**IRR**	**95% CI**	**IRR**	**95% CI**
40-64	0.763	(0.47-1.58)	0.284	(0.00-0.65)
65-84	0.224	(0.13-0.31)	0.660	(0.42-0.94)
**Age**	**Never-smoking Female/Male, 2016-2019**		**Ever-smoking Female/Male, 2016-2019**	
	**IRR**	**95% CI**	**IRR**	**95% CI**
40-64	0.297	(0.04-0.85)	0.572	(0.16-1.08)
65-84	0.198	(0.10-0.37)	1.004	(0.61-1.58)

© 2023 Chien et al. *JAMA Network Open.*

(2) smoking status

Age	Ever/never smoking Female, 2011-2019		Ever/never smoking Male, 2011-2019	
	IRR	95% CI	IRR	95% CI
40-44	0.000	(0.00-0.00)	Inf	NA
45-49	0.000	(0.00-0.00)	7.698	(3.13-21.43)
50-54	6.400	(0.00-30.21)	5.898	(2.53-25.93)
55-59	17.101	(0.00-102.46)	6.504	(3.66-14.38)
60-64	11.195	(2.58-24.11)	13.272	(7.36-32.67)
65-69	24.526	(9.63-65.97)	8.866	(5.45-14.56)
70-74	25.075	(11.73-66.77)	10.383	(7.38-17.28)
75-79	53.408	(25.06-133.77)	9.186	(6.24-13.51)
80-84	22.468	(7.78-78.87)	6.517	(4.64-10.16)

Age	Ever/never smoking Female, 2011-2015		Ever/never smoking Male, 2011-2015	
	IRR	95% CI	IRR	95% CI
40-64	2.691	(0.62-5.50)	7.234	(4.33-13.46)
65-84	18.191	(8.73-29.54)	6.181	(4.61-7.73)

Age	Ever/never smoking Female, 2016-2019		Ever/never smoking Male, 2016-2019	
	IRR	95% CI	IRR	95% CI
40-64	21.444	(6.19-76.17)	11.145	(6.48-32.54)
65-84	61.762	(30.32-117.33)	12.205	(8.66-18.42)

© 2023 Chien et al. *JAMA Network Open.*

(3) periods

Age	Never-smoking Female, 2016-2019/2011-2015		Never-smoking Male, 2016-2019/2011-2015	
	**IRR**	**95% CI**	**IRR**	**95% CI**
40-64	0.276	(0.08-0.58)	0.708	(0.22-1.58)
65-84	0.431	(0.18-0.73)	0.487	(0.33-0.71)
**Age**	**Ever-smoking Female, 2016-2019/2011-2015**		**Ever-smoking Male, 2016-2019/2011-2015**	
	**IRR**	**95% CI**	**IRR**	**95% CI**
40-64	2.199	(0.53-8.78)	1.091	(0.84-1.40)
65-84	1.463	(0.82-3.57)	0.961	(0.84-1.11)

© 2023 Chien et al. *JAMA Network Open.*

eTable 19C. Age-specific late-stage 3&4 lung SCC incidence rate ratios and their 95% confidence intervals comparing sex (1), smoking status (2), periods (3).

(1) sex

Age	Never-smoking Female/Male, 2011-2019		Ever-smoking Female/Male, 2011-2019	
	IRR	95% CI	IRR	95% CI
40-44	0.983	(0.39-2.26)	0.754	(0.31-1.53)
45-49	0.738	(0.49-1.26)	0.498	(0.19-0.94)
50-54	0.776	(0.54-1.35)	0.577	(0.33-0.83)
55-59	0.609	(0.47-0.85)	0.573	(0.36-0.87)
60-64	0.548	(0.46-0.68)	0.439	(0.30-0.62)
65-69	0.359	(0.28-0.46)	0.520	(0.35-0.75)
70-74	0.408	(0.32-0.52)	0.512	(0.33-0.68)
75-79	0.292	(0.23-0.35)	0.397	(0.26-0.53)
80-84	0.243	(0.19-0.29)	0.584	(0.40-0.72)

Age	Never-smoking Female/Male, 2011-2015		Ever-smoking Female/Male, 2011-2015	
	IRR	95% CI	IRR	95% CI
40-44	1.844	(0.70-9.28)	0.452	(0.00-1.29)
45-49	0.708	(0.37-1.42)	0.303	(0.05-0.70)
50-54	0.874	(0.60-1.43)	0.446	(0.15-0.81)
55-59	0.586	(0.38-0.85)	0.518	(0.24-0.83)
60-64	0.542	(0.38-0.84)	0.399	(0.20-0.57)
65-69	0.354	(0.25-0.49)	0.477	(0.22-0.73)

© 2023 Chien et al. *JAMA Network Open.*

70-74	0.381	(0.27-0.56)	0.434	(0.27-0.68)
75-79	0.247	(0.19-0.32)	0.402	(0.24-0.61)
80-84	0.259	(0.17-0.35)	0.618	(0.40-0.89)

Age	Never-smoking Female/Male, 2016-2019		Ever-smoking Female/Male, 2016-2019	
	**IRR**	**95% CI**	**IRR**	**95% CI**
40-44	0.407	(0.06-1.07)	1.140	(0.21-2.57)
45-49	0.770	(0.38-2.77)	0.727	(0.28-1.35)
50-54	0.683	(0.40-1.32)	0.732	(0.30-1.20)
55-59	0.641	(0.41-0.95)	0.649	(0.34-1.07)
60-64	0.553	(0.42-0.83)	0.484	(0.23-0.71)
65-69	0.359	(0.26-0.46)	0.566	(0.27-0.90)
70-74	0.438	(0.27-0.61)	0.646	(0.41-0.94)
75-79	0.357	(0.27-0.47)	0.391	(0.19-0.63)
80-84	0.226	(0.16-0.31)	0.543	(0.29-0.78)

(2) smoking status

Age	Ever/never smoking Female, 2011-2019		Ever/never smoking Male, 2011-2019	
	**IRR**	**95% CI**	**IRR**	**95% CI**
40-44	3.418	(0.90-8.30)	4.459	(2.78-10.41)
45-49	3.061	(1.32-5.66)	4.536	(3.24-7.11)
50-54	4.663	(2.59-6.69)	6.275	(4.57-9.51)
55-59	5.830	(3.67-8.80)	6.197	(5.09-8.25)

© 2023 Chien et al. *JAMA Network Open.*

Age	IRR	95% CI	IRR	95% CI
60-64	5.492	(3.41-7.75)	6.854	(5.67-8.12)
65-69	8.098	(5.24-12.61)	5.598	(4.81-6.73)
70-74	11.005	(6.35-16.11)	8.771	(7.48-10.47)
75-79	9.067	(6.04-13.03)	6.666	(5.88-7.80)
80-84	16.153	(10.96-22.54)	6.733	(5.82-7.71)
**Age**	**Ever/never smoking Female, 2011-2015**		**Ever/never smoking Male, 2011-2015**	
	**IRR**	**95% CI**	**IRR**	**95% CI**
40-44	1.467	(0.00-3.83)	5.978	(2.54-30.00)
45-49	1.694	(0.00-4.56)	3.956	(2.40-9.05)
50-54	3.569	(1.78-6.85)	6.991	(4.03-10.76)
55-59	4.923	(2.68-9.51)	5.568	(4.26-7.51)
60-64	5.087	(2.60-8.34)	6.913	(5.61-9.17)
65-69	6.973	(3.47-11.06)	5.172	(4.31-6.14)
70-74	8.535	(5.00-12.49)	7.487	(6.38-9.03)
75-79	8.861	(5.11-13.43)	5.445	(4.55-6.69)
80-84	16.411	(10.55-24.31)	6.884	(5.75-8.67)
**Age**	**Ever/never smoking Female, 2016-2019**		**Ever/never smoking Male, 2016-2019**	
	**IRR**	**95% CI**	**IRR**	**95% CI**
40-44	10.094	(0.71-32.46)	3.605	(1.55-12.60)
45-49	5.138	(1.38-11.75)	5.436	(3.06-13.45)
50-54	5.948	(1.59-10.76)	5.556	(3.85-8.65)
55-59	7.175	(3.17-11.68)	7.081	(5.09-10.21)

© 2023 Chien et al. *JAMA Network Open.*

60-64	5.932	(3.28-9.40)	6.771	(5.16-9.18)
65-69	9.344	(5.06-17.02)	5.938	(4.83-7.79)
70-74	15.692	(10.36-27.48)	10.643	(8.11-13.49)
75-79	9.292	(5.53-15.86)	8.498	(7.14-10.91)
80-84	15.990	(9.04-24.61)	6.660	(5.36-8.35)

(3) periods

Age	Never-smoking Female, 2016-2019/2011-2015		Never-smoking Male, 2016-2019/2011-2015	
	**IRR**	**95% CI**	**IRR**	**95% CI**
40-44	0.357	(0.12-0.98)	1.619	(0.59-6.05)
45-49	0.871	(0.43-1.55)	0.801	(0.38-1.53)
50-54	0.969	(0.63-1.53)	1.239	(0.60-2.48)
55-59	0.870	(0.60-1.24)	0.795	(0.47-1.24)
60-64	0.996	(0.75-1.38)	0.978	(0.69-1.36)
65-69	0.792	(0.55-1.11)	0.780	(0.60-0.97)
70-74	0.764	(0.56-1.05)	0.664	(0.48-0.86)
75-79	0.925	(0.64-1.29)	0.638	(0.47-0.85)
80-84	0.932	(0.62-1.42)	1.068	(0.82-1.46)
**Age**	**Ever-smoking Female, 2016-2019/2011-2015**		**Ever-smoking Male, 2016-2019/2011-2015**	
	**IRR**	**95% CI**	**IRR**	**95% CI**
40-44	2.459	(0.44-8.56)	0.976	(0.54-1.47)
45-49	2.641	(0.96-13.40)	1.101	(0.80-1.42)

© 2023 Chien et al. *JAMA Network Open.*

50-54	1.615	(0.65-4.20)	0.985	(0.81-1.19)
55-59	1.268	(0.70-2.80)	1.011	(0.84-1.17)
60-64	1.162	(0.55-2.56)	0.958	(0.83-1.09)
65-69	1.061	(0.54-2.55)	0.895	(0.79-1.00)
70-74	1.405	(0.82-2.69)	0.943	(0.86-1.06)
75-79	0.970	(0.48-1.85)	0.996	(0.89-1.10)
80-84	0.908	(0.50-1.94)	1.034	(0.92-1.14)

© 2023 Chien et al. *JAMA Network Open.*

eTable 20. Histology codes for lung SCC that appeared in the TCR for each year from 2011 to 2019.

2011	2012	2013	2014	2015	2016	2017	2018	2019
80523	80523	80523	80523	80523	80703	80703	80523	80703
80713	80713	80713	80713	80713	80723	80723	80713	80723
80733	80733	80733	80733	80733	80833	80743	80743	80743
80823	80833	80753	80763	80833	80713	80833	80833	80713
80843	80703	90503	90523	80703	80743	80713	80703	80733
80703	80723	80703	80703	80723	80843	80733	80723	80833
80723	80743	80723	80723	80743		80753	80763	
80743	80843	80743	80743					
80833		80843	80833					

© 2023 Chien et al. *JAMA Network Open.*

eTable 21. Age- and sex-specific number of patients with invasive lung SCLC diagnosedfrom 2011 to 2019 according to the TCR.

Year	2011-2015		2016-2019	
Sex / Age	M	F	M	F
40-44	33	8	38	5
45-49	110	14	82	12
50-54	214	24	193	30
55-59	340	47	324	39
60-64	549	57	477	66
65-69	462	55	570	60
70-74	597	61	471	55
75-79	559	48	435	45
80-84	476	59	319	40

© 2023 Chien et al. *JAMA Network Open.*

eTable 22A. Age-, sex-, and calendar year-specific percentages of patients with late-stage (stage2—4) SCLC among those with all stage (1—4) of invasive SCLC, estimated using the TCRLF for each year from 2011 to 2019.

**Year**	**2011-2015**		**2016-2019**	
Sex / Age	M	F	M	F
40-44	0.968	0.875	1.000	1.000
45-49	0.991	1.000	1.000	1.000
50-54	0.990	0.958	0.984	0.966
55-59	0.991	0.978	0.990	0.974
60-64	0.992	0.962	0.974	0.970
65-69	0.980	1.000	0.975	0.983
70-74	0.984	0.945	0.987	1.000
75-79	0.969	0.955	0.973	0.930
80-84	0.954	0.940	0.973	0.973

© 2023 Chien et al. *JAMA Network Open.*

eTable 22B. Age-, sex- and calendar year-specific percentages of ever-smokers among corresponding patients with late-stage (2—4) SCLC in Taiwan.

Year	2011-2015		2016-2019	
Sex / Age	M	F	M	F
40-44	0.967	0.571	0.973	0.200
45-49	0.912	0.692	0.911	0.600
50-54	0.927	0.435	0.938	0.519
55-59	0.938	0.605	0.936	0.611
60-64	0.927	0.583	0.913	0.607
65-69	0.912	0.612	0.893	0.589
70-74	0.932	0.688	0.902	0.667
75-79	0.866	0.550	0.878	0.452
80-84	0.799	0.429	0.852	0.400

© 2023 Chien et al. *JAMA Network Open.*

eTable 22C. Age-, sex- and year-specific percentages of ever-smokers among corresponding patients with early-stage (1) SCLC in Taiwan.

Year	2011-2015		2016-2019	
Sex / Age	M	F	M	F
40-64	0.909	0.200	0.944	0.000
65-84	0.854	0.000	0.923	0.000

© 2023 Chien et al. *JAMA Network Open.*

eTable 22D. Age-, sex-, and calendar year-specific percentages of patients with stage 3&4 SCLC among those of all stages (1—4) of invasive SCLC, estimated using the TCRLF for each year from 2011 to 2019.

Year	2011-2015		2016-2019	
Age \ Sex	M	F	M	F
40-44	0.968	0.875	1.000	0.600
45-49	0.972	1.000	0.963	1.000
50-54	0.980	0.958	0.968	0.966
55-59	0.979	0.978	0.984	0.974
60-64	0.973	0.943	0.959	0.970
65-69	0.970	0.980	0.958	0.966
70-74	0.958	0.945	0.969	1.000
75-79	0.954	0.932	0.957	0.907
80-84	0.931	0.920	0.963	0.946

© 2023 Chien et al. *JAMA Network Open.*

eTable 22E. Age-, sex- and year-specific percentages of ever-smokers among corresponding patients with stage 3&4 SCLC in Taiwan.

**Year**	**2011-2015**		**2016-2019**	
Sex / Age	M	F	M	F
40-44	0.967	0.571	0.973	0.333
45-49	0.920	0.692	0.908	0.600
50-54	0.926	0.435	0.943	0.519
55-59	0.937	0.605	0.936	0.611
60-64	0.926	0.596	0.914	0.607
65-69	0.911	0.625	0.894	0.582
70-74	0.934	0.688	0.901	0.667
75-79	0.865	0.564	0.876	0.433
80-84	0.797	0.439	0.850	0.379

© 2023 Chien et al. *JAMA Network Open.*

eTable 23A. Estimated age- and sex-specific number of patients with late-stage lung SCLC in the TCR by smoking status.

	Never-smokers			
Year	2011-2015		2016-2019	
Age \ Sex	M	F	M	F
40-44	1.065	3.000	1.027	4.000
45-49	9.614	4.308	7.266	4.800
50-54	15.530	13.000	11.803	13.946
55-59	20.864	18.168	20.391	14.778
60-64	39.700	22.854	40.649	25.180
65-69	40.029	21.327	59.165	24.218
70-74	40.087	18.023	45.374	18.333
75-79	72.867	20.618	51.727	22.956
80-84	91.327	31.691	45.978	23.351

	Ever-smokers			
Year	2011-2015		2016-2019	
Age \ Sex	M	F	M	F
40-44	30.871	4.000	36.973	1.000
45-49	99.348	9.692	74.734	7.200
50-54	196.341	10.000	178.117	15.019
55-59	316.083	27.787	300.494	23.222
60-64	505.077	31.995	424.068	38.820
65-69	412.521	33.673	496.326	34.748
70-74	547.470	39.650	419.429	36.667
75-79	468.933	25.200	371.715	18.905
80-84	362.838	23.769	264.372	15.568

© 2023 Chien et al. *JAMA Network Open.*

eTable 23B. Estimated age- and sex-specific number of patients with early-stage lung SCLC in the TCR by smoking status.

	Never-smokers			
Year	2011-2015		2016-2019	
Age \ Sex	M	F	M	F
40-64	1.046	4.156	1.027	4.034
65-84	8.448	9.049	3.147	5.255

	Ever-smokers			
Year	2011-2015		2016-2019	
Age \ Sex	M	F	M	F
40-64	10.462	1.039	17.452	0.000
65-84	49.480	0.000	37.768	0.000

© 2023 Chien et al. *JAMA Network Open.*

eTable 23C. Estimated age- and sex-specific number of patients with stage 3&4 lung SCLC in the TCR by smoking status.

	Never-smokers			
**Year**	**2011-2015**		**2016-2019**	
Sex / Age	M	F	M	F
40-44	1.065	3.000	1.027	2.000
45-49	8.551	4.308	7.276	4.800
50-54	15.536	13.000	10.738	13.946
55-59	20.870	18.168	20.395	14.778
60-64	39.735	21.738	39.501	25.180
65-69	39.853	20.213	58.126	24.226
70-74	37.665	18.023	45.213	18.333
75-79	71.800	19.497	51.710	23.128
80-84	89.881	30.450	46.066	23.486

	Ever-smokers			
**Year**	**2011-2015**		**2016-2019**	
Sex / Age	M	F	M	F
40-44	30.871	4.000	36.973	1.000
45-49	98.336	9.692	71.724	7.200
50-54	194.205	10.000	176.102	15.019
55-59	312.004	27.787	298.413	23.222
60-64	494.484	32.035	418.051	38.820
65-69	408.497	33.688	488.038	33.705
70-74	534.154	39.650	411.327	36.667
75-79	461.400	25.231	364.377	17.686
80-84	353.367	23.830	261.039	14.352

© 2023 Chien et al. *JAMA Network Open.*

eTable 24. Number of patients with invasive lung SCLC in TCR, TCRLF, having known smoking status (ever-smoker or never-smoker), and having a known number of pack-years smoked and a known number of years since smoking quitting, for 2011—2019.

Year	TCR	TCRLF	Smoking status known	Pack-year and quit time known
2011	831	780	719	486
2012	792	756	691	483
2013	815	791	723	511
2014	815	792	710	494
2015	863	853	753	505
2016	891	874	789	545
2017	883	865	781	531
2018	855	833	748	491
2019	938	926	828	537
Sum	7683	7470	6742	4583

© 2023 Chien et al. *JAMA Network Open.*

eTable 25A. Age-specific incidence rates for late-stage SCLC in the periods 2011—2019, 2011—2015, and 2016—2019 by sex and smoking status: never-smoking female (1), never-smoking male (2), ever-smoking female (3), ever-smoking male (4).

(1) never-smoking female

Age	Never-smoking Female					
	2011-2019		2011-2015		2016-2019	
	**rate**	**95% Cl**	**rate**	**95% Cl**	**rate**	**95% Cl**
40-44	0.092	(0.03-0.14)	0.072	(0.00-0.17)	0.116	(0.03-0.23)
45-49	0.119	(0.05-0.20)	0.101	(0.02-0.21)	0.145	(0.03-0.24)
50-54	0.355	(0.22-0.49)	0.308	(0.14-0.50)	0.412	(0.21-0.62)
55-59	0.461	(0.29-0.63)	0.474	(0.26-0.70)	0.447	(0.21-0.67)
60-64	0.803	(0.55-1.00)	0.750	(0.43-1.05)	0.859	(0.51-1.19)
65-69	1.102	(0.80-1.43)	1.136	(0.64-1.60)	1.072	(0.66-1.46)
70-74	1.226	(0.84-1.62)	1.104	(0.61-1.59)	1.376	(0.75-2.03)
75-79	1.795	(1.29-2.28)	1.636	(0.95-2.38)	2.001	(1.13-2.79)
80-84	3.343	(2.49-4.25)	3.718	(2.46-4.93)	2.934	(1.76-4.15)

(2) never-smoking male

Age	Never-smoking Male					
	2011-2019		2011-2015		2016-2019	
	**rate**	**95% Cl**	**rate**	**95% Cl**	**rate**	**95% Cl**
40-44	0.055	(0.00-0.13)	0.057	(0.00-0.16)	0.053	(0.00-0.15)
45-49	0.480	(0.23-0.68)	0.516	(0.21-0.80)	0.440	(0.12-0.79)
50-54	0.849	(0.53-1.18)	0.878	(0.45-1.30)	0.817	(0.35-1.25)
55-59	1.264	(0.89-1.69)	1.221	(0.70-1.70)	1.312	(0.77-1.87)
60-64	2.863	(2.28-3.46)	2.855	(1.94-3.74)	2.871	(1.98-3.67)
65-69	5.382	(4.29-6.40)	5.105	(3.57-6.89)	5.582	(4.25-7.08)
70-74	6.166	(4.82-7.48)	5.683	(3.97-7.66)	6.626	(4.82-8.47)
75-79	12.372	(10.32-14.49)	14.787	(11.36-17.86)	10.048	(7.19-12.82)
80-84	18.447	(15.28-21.58)	22.724	(17.91-27.37)	13.355	(9.30-17.14)

(3) ever-smoking female

© 2023 Chien et al. *JAMA Network Open.*

Age	Ever-smoking Female					
	2011-2019		2011-2015		2016-2019	
	rate	95% Cl	rate	95% Cl	rate	95% Cl
40-44	0.822	(0.16-1.64)	1.195	(0.00-2.09)	0.365	(0.00-1.10)
45-49	3.271	(1.74-4.63)	3.337	(1.03-5.16)	3.159	(0.88-5.70)
50-54	6.295	(3.79-8.58)	4.469	(1.79-6.70)	8.710	(4.64-13.34)
55-59	18.323	(13.29-23.71)	17.283	(10.57-23.01)	19.745	(11.90-28.06)
60-64	34.129	(26.48-42.37)	29.750	(19.53-39.05)	38.763	(26.96-50.93)
65-69	69.500	(53.88-87.41)	65.431	(42.75-87.44)	74.094	(49.04-98.09)
70-74	104.922	(82.45-127.79)	88.085	(62.21-113.30)	132.077	(86.46-172.91)
75-79	75.848	(54.61-98.98)	72.738	(46.18-101.03)	78.926	(45.93-112.73)
80-84	98.353	(69.98-129.96)	101.477	(59.78-140.90)	93.837	(48.22-138.65)

(4) ever-smoking male

Age	Ever-smoking Male					
	2011-2019		2011-2015		2016-2019	
	rate	95% Cl	rate	95% Cl	rate	95% Cl
40-44	1.582	(1.21-1.98)	1.190	(0.77-1.58)	2.179	(1.42-2.83)
45-49	3.811	(3.24-4.36)	3.656	(2.94-4.34)	4.038	(3.08-4.86)
50-54	7.853	(7.03-8.70)	7.338	(6.32-8.37)	8.511	(7.31-9.70)
55-59	15.366	(14.08-16.60)	14.295	(12.62-15.78)	16.679	(14.65-18.59)
60-64	29.957	(27.93-31.92)	31.031	(28.32-33.73)	28.780	(26.06-31.69)
65-69	44.585	(41.70-47.34)	41.969	(37.75-45.99)	47.028	(43.02-51.16)
70-74	80.222	(75.11-85.14)	75.901	(69.46-82.35)	86.717	(78.36-95.31)
75-79	90.601	(84.29-96.90)	87.272	(79.47-95.10)	95.194	(84.52-105.51)
80-84	102.213	(94.40-110.54)	94.250	(84.42-104.42)	115.773	(102.47-129.62)

© 2023 Chien et al. *JAMA Network Open.*

eTable 25B. Age-specific incidence rates for early-stage SCLC in the periods 2011—2019, 2011—2015, and 2016—2019 by sex and smoking status: never-smoking female (1), never-smoking male (2), ever-smoking female (3), ever-smoking male (4).

(1) never-smoking female

Never-smoking Female							
**Age**	2011-2019		**Age**	2011-2015		2016-2019	
	**rate**	**95% Cl**		**rate**	**95% Cl**	**rate**	**95% Cl**
40-44	0.013	(0.00-0.04)	40-64	0.021	(0.01-0.05)*	0.025	(0.01-0.06)*
45-49	NA	NA	65-84	0.161	(0.05-0.28)	0.095	(0.02-0.18)
50-54	0.027	(0.00-0.07)					
55-59	0.014	(0.00-0.06)					
60-64	0.069	(0.02-0.13)					
65-69	0.026	(0.00-0.07)					
70-74	0.111	(0.00-0.24)					
75-79	0.222	(0.04-0.42)					
80-84	0.276	(0.06-0.49)					

*Use the exact binomial 95% confidence interval.

(2) never-smoking male

Never-smoking Male							
**Age**	2011-2019		**Age**	2011-2015		2016-2019	
	**rate**	**95% Cl**		**rate**	**95% Cl**	**rate**	**95% Cl**
40-44	0.000	(0.00-0.00)	40-64	0.012	(0.00-0.03)	0.013	(0.00-0.04)
45-49	0.029	(0.00-0.09)	65-84	0.354	(0.13-0.59)	0.121	(0.00-0.27)
50-54	0.000	(0.00-0.00)					
55-59	0.032	(0.00-0.09)					
60-64	0.000	(0.00-0.00)					
65-69	0.000	(0.00-0.00)					
70-74	0.086	(0.00-0.22)					
75-79	0.456	(0.10-0.79)					
80-84	0.785	(0.13-1.34)					

(3) ever-smoking female

© 2023 Chien et al. *JAMA Network Open.*

Ever-smoking Female							
Age	2011-2019		Age	2011-2015		2016-2019	
	**rate**	**95% Cl**		**rate**	**95% Cl**	**rate**	**95% Cl**
40-44	0.000	(0.00-0.00)	40-64	0.093	(0.00-0.27)	0.000	(0.00-0.00)
45-49	NA	NA	65-84	0.000	(0.00-0.00)	0.000	(0.00-0.00)
50-54	0.000	(0.00-0.00)					
55-59	0.368	(0.00-1.08)					
60-64	0.000	(0.00-0.00)					
65-69	0.000	(0.00-0.00)					
70-74	0.000	(0.00-0.00)					
75-79	0.000	(0.00-0.00)					
80-84	0.000	(0.00-0.00)					

(4) ever-smoking male

Ever-smoking Male							
Age	2011-2019		Age	2011-2015		2016-2019	
	**rate**	**95% Cl**		**rate**	**95% Cl**	**rate**	**95% Cl**
40-44	0.024	(0.00-0.07)	40-64	0.088	(0.03-0.14)	0.196	(0.10-0.28)
45-49	0.000	(0.00-0.00)	65-84	1.884	(1.33-2.40)	1.750	(1.20-2.32)
50-54	0.110	(0.02-0.21)					
55-59	0.128	(0.03-0.25)					
60-64	0.537	(0.29-0.74)					
65-69	1.176	(0.69-1.62)					
70-74	1.197	(0.58-1.74)					
75-79	2.602	(1.62-3.67)					
80-84	4.010	(2.45-5.38)					

© 2023 Chien et al. *JAMA Network Open.*

eTable 25C. Age-specific incidence rates for stage 3&4 SCLC in the periods 2011—2019, 2011—2015, and 2016—2019 by sex and smoking status: never-smoking female (1), never-smoking male (2), ever-smoking female (3), ever-smoking male (4).

(1) never-smoking female

Age	Never-smoking Female					
	2011-2019		2011-2015		2016-2019	
	rate	95% Cl	rate	95% Cl	rate	95% Cl
40-44	0.066	(0.00-0.10)	0.072	(0.00-0.17)	0.058	(0.00-0.14)
45-49	0.119	(0.05-0.20)	0.101	(0.02-0.21)	0.145	(0.03-0.24)
50-54	0.355	(0.22-0.49)	0.308	(0.14-0.50)	0.412	(0.21-0.62)
55-59	0.461	(0.29-0.63)	0.474	(0.26-0.70)	0.447	(0.21-0.67)
60-64	0.785	(0.54-0.99)	0.713	(0.39-1.02)	0.859	(0.51-1.19)
65-69	1.076	(0.77-1.40)	1.077	(0.64-1.54)	1.073	(0.66-1.46)
70-74	1.226	(0.84-1.62)	1.104	(0.61-1.59)	1.376	(0.75-2.03)
75-79	1.750	(1.25-2.24)	1.547	(0.87-2.22)	2.016	(1.22-2.79)
80-84	3.276	(2.37-4.13)	3.573	(2.35-4.81)	2.951	(1.76-4.15)

(2) never-smoking male

Age	Never-smoking Male					
	2011-2019		2011-2015		2016-2019	
	rate	95% Cl	rate	95% Cl	rate	95% Cl
40-44	0.055	(0.00-0.13)	0.057	(0.00-0.16)	0.053	(0.00-0.15)
45-49	0.450	(0.20-0.65)	0.459	(0.16-0.75)	0.441	(0.12-0.79)
50-54	0.816	(0.50-1.15)	0.878	(0.45-1.30)	0.743	(0.28-1.18)
55-59	1.265	(0.89-1.69)	1.222	(0.70-1.70)	1.312	(0.77-1.87)
60-64	2.824	(2.24-3.42)	2.858	(1.94-3.74)	2.790	(1.84-3.60)
65-69	5.315	(4.28-6.40)	5.082	(3.57-6.50)	5.484	(4.15-6.98)
70-74	5.983	(4.68-7.34)	5.340	(3.69-7.09)	6.602	(4.68-8.62)
75-79	12.263	(10.22-14.49)	14.570	(11.16-17.65)	10.044	(7.19-12.82)
80-84	18.270	(15.15-21.44)	22.364	(17.67-27.11)	13.381	(9.59-17.14)

(3) ever-smoking female

© 2023 Chien et al. *JAMA Network Open.*

Age	Ever-smoking Female					
	2011-2019		2011-2015		2016-2019	
	**rate**	**95% Cl**	**rate**	**95% Cl**	**rate**	**95% Cl**
40-44	0.822	(0.16-1.32)	1.195	(0.00-2.09)	0.365	(0.01-2.04)*
45-49	3.271	(1.74-4.63)	3.337	(1.03-5.16)	3.159	(0.88-5.70)
50-54	6.295	(3.79-8.58)	4.469	(1.79-6.70)	8.710	(4.64-13.34)
55-59	18.323	(13.29-23.71)	17.283	(10.57-23.01)	19.745	(11.90-28.06)
60-64	34.145	(26.48-42.37)	29.787	(19.53-40.89)	38.763	(26.96-50.93)
65-69	68.422	(52.87-85.40)	65.458	(42.75-87.44)	71.871	(46.91-93.82)
70-74	104.922	(82.45-127.79)	88.085	(62.21-113.30)	132.077	(86.46-172.91)
75-79	74.028	(52.90-97.28)	72.827	(46.18-101.03)	73.838	(41.75-108.45)
80-84	95.434	(67.48-124.96)	101.741	(59.78-140.90)	86.512	(42.20-132.62)

*Use the exact binomial 95% confidence interval.

(4) ever-smoking male

Age	Ever-smoking Male					
	2011-2019		2011-2015		2016-2019	
	**rate**	**95% Cl**	**rate**	**95% Cl**	**rate**	**95% Cl**
40-44	1.582	(1.21-1.98)	1.190	(0.77-1.58)	2.179	(1.42-2.83)
45-49	3.722	(3.17-4.29)	3.618	(2.91-4.31)	3.876	(2.92-4.75)
50-54	7.766	(6.94-8.62)	7.258	(6.24-8.26)	8.414	(7.21-9.60)
55-59	15.212	(13.98-16.45)	14.110	(12.39-15.60)	16.564	(14.82-18.37)
60-64	29.422	(27.41-31.34)	30.380	(27.71-32.99)	28.372	(25.72-31.22)
65-69	43.981	(41.11-46.75)	41.560	(37.44-45.68)	46.243	(42.26-50.50)
70-74	78.445	(73.36-83.07)	74.055	(67.79-80.55)	85.042	(76.91-93.45)
75-79	88.999	(82.88-95.28)	85.870	(77.98-93.80)	93.315	(82.98-103.46)
80-84	100.123	(91.64-108.42)	91.789	(81.83-102.08)	114.314	(101.60-128.31)

© 2023 Chien et al. *JAMA Network Open.*

eTable 26A. Age-specific late-stage lung SCLC incidence rate ratios and their 95% confidence intervals comparing sex (1), smoking status (2), periods (3).

(1) sex

Age	Never-smoking Female/Male, 2011-2019		Ever-smoking Female/Male, 2011-2019	
	IRR	95% CI	IRR	95% CI
40-44	1.682	(0.38-5.53)	0.520	(0.10-1.12)
45-49	0.248	(0.07-0.46)	0.858	(0.48-1.44)
50-54	0.418	(0.25-0.91)	0.802	(0.53-1.26)
55-59	0.365	(0.25-0.60)	1.192	(0.85-1.50)
60-64	0.280	(0.20-0.39)	1.139	(0.90-1.42)
65-69	0.205	(0.15-0.30)	1.559	(1.15-2.06)
70-74	0.199	(0.11-0.30)	1.308	(0.99-1.69)
75-79	0.145	(0.10-0.21)	0.837	(0.54-1.09)
80-84	0.181	(0.12-0.26)	0.962	(0.66-1.32)

Age	Never-smoking Female/Male, 2011-2015		Ever-smoking Female/Male, 2011-2015	
	IRR	95% CI	IRR	95% CI
40-44	1.270	(0.00-3.05)	1.005	(0.13-2.53)
45-49	0.196	(0.02-0.51)	0.913	(0.43-1.58)
50-54	0.351	(0.15-0.68)	0.609	(0.30-1.02)
55-59	0.388	(0.18-0.74)	1.209	(0.85-1.75)
60-64	0.263	(0.12-0.44)	0.959	(0.52-1.43)
65-69	0.223	(0.11-0.37)	1.559	(1.05-2.26)

© 2023 Chien et al. *JAMA Network Open.*

70-74	0.194	(0.10-0.32)	1.161	(0.79-1.63)
75-79	0.111	(0.06-0.18)	0.833	(0.54-1.16)
80-84	0.164	(0.11-0.26)	1.077	(0.69-1.67)

Age	Never-smoking Female/Male, 2016-2019		Ever-smoking Female/Male, 2016-2019	
	**IRR**	**95% CI**	**IRR**	**95% CI**
40-44	2.196	(0.11-4.19)	0.168	(0.00-0.80)
45-49	0.329	(0.06-1.99)	0.782	(0.17-1.31)
50-54	0.504	(0.23-1.04)	1.023	(0.56-1.62)
55-59	0.341	(0.15-0.71)	1.184	(0.70-1.77)
60-64	0.299	(0.17-0.49)	1.347	(0.94-1.85)
65-69	0.192	(0.12-0.29)	1.576	(1.03-2.23)
70-74	0.208	(0.11-0.37)	1.523	(1.02-2.12)
75-79	0.199	(0.10-0.31)	0.829	(0.52-1.24)
80-84	0.220	(0.13-0.38)	0.811	(0.41-1.35)

(2) smoking status

Age	Ever/never smoking Female, 2011-2019		Ever/never smoking Male, 2011-2019	
	**IRR**	**95% CI**	**IRR**	**95% CI**
40-44	8.953	(2.32-45.40)	28.980	(12.17-71.34)
45-49	27.426	(13.29-77.46)	7.938	(4.99-14.00)
50-54	17.722	(10.49-31.85)	9.248	(6.62-16.67)
55-59	39.718	(25.40-63.27)	12.154	(9.38-17.33)

© 2023 Chien et al. *JAMA Network Open.*

60-64	42.527	(30.24-62.64)	10.462	(8.48-14.95)
65-69	63.068	(41.05-81.60)	8.283	(6.88-10.56)
70-74	85.568	(57.06-142.54)	13.010	(10.37-17.33)
75-79	42.257	(26.35-66.89)	7.323	(6.28-9.21)
80-84	29.422	(18.12-44.02)	5.541	(4.51-6.88)

**Age**	**Ever/never smoking Female, 2011-2015**		**Ever/never smoking Male, 2011-2015**	
	**IRR**	**95% CI**	**IRR**	**95% CI**
40-44	16.622	(1.93-74.92)	21.014	(4.77-30.15)
45-49	33.035	(11.87-139.04)	7.091	(3.84-20.92)
50-54	14.513	(6.92-33.28)	8.361	(4.78-17.87)
55-59	36.488	(23.35-70.29)	11.703	(8.07-21.58)
60-64	39.681	(22.03-81.37)	10.868	(8.12-16.21)
65-69	57.590	(37.83-101.88)	8.222	(6.01-10.82)
70-74	79.804	(41.51-154.30)	13.356	(10.00-20.06)
75-79	44.462	(23.99-82.11)	5.902	(4.74-7.78)
80-84	27.292	(15.04-55.30)	4.148	(3.38-5.56)

**Age**	**Ever/never smoking Female, 2016-2019**		**Ever/never smoking Male, 2016-2019**	
	**IRR**	**95% CI**	**IRR**	**95% CI**
40-44	3.154	(0.00-14.70)	41.297	(9.56-54.28)
45-49	21.835	(5.38-82.31)	9.180	(4.32-32.03)
50-54	21.139	(8.44-55.66)	10.418	(6.16-19.36)
55-59	44.162	(20.57-85.72)	12.716	(7.86-20.29)

© 2023 Chien et al. *JAMA Network Open.*

60-64	45.117	(25.41-76.31)	10.024	(7.06-13.84)
65-69	69.098	(39.59-120.75)	8.425	(6.55-11.96)
70-74	95.996	(50.15-181.72)	13.088	(10.28-18.31)
75-79	39.438	(20.59-81.74)	9.474	(7.40-12.67)
80-84	31.980	(12.69-58.34)	8.669	(6.58-12.93)

(3) periods

**Age**	**Never-smoking Female, 2016-2019/2011-2015**		**Never-smoking Male, 2016-2019/2011-2015**	
	**IRR**	**95% CI**	**IRR**	**95% CI**
40-44	1.611	(0.11-7.34)	0.932	(0.00-3.49)
45-49	1.432	(0.37-9.00)	0.853	(0.24-1.94)
50-54	1.338	(0.43-2.84)	0.931	(0.42-2.20)
55-59	0.944	(0.50-2.06)	1.074	(0.44-2.34)
60-64	1.146	(0.68-1.77)	1.006	(0.59-1.58)
65-69	0.944	(0.50-2.24)	1.093	(0.73-1.91)
70-74	1.247	(0.56-2.26)	1.166	(0.87-1.81)
75-79	1.223	(0.70-2.32)	0.680	(0.51-1.02)
80-84	0.789	(0.43-1.35)	0.588	(0.40-0.86)
**Age**	**Ever-smoking Female, 2016-2019/2011-2015**		**Ever-smoking Male, 2016-2019/2011-2015**	
	**IRR**	**95% CI**	**IRR**	**95% CI**
40-44	0.306	(0.00-3.06)	1.831	(1.13-3.05)
45-49	0.947	(0.19-2.91)	1.105	(0.79-1.46)

© 2023 Chien et al. *JAMA Network Open.*

50-54	1.949	(0.75-5.52)	1.160	(0.92-1.41)
55-59	1.142	(0.57-1.89)	1.167	(0.96-1.38)
60-64	1.303	(0.71-1.88)	0.927	(0.80-1.04)
65-69	1.132	(0.68-1.71)	1.121	(0.99-1.25)
70-74	1.499	(0.81-2.46)	1.143	(1.01-1.30)
75-79	1.085	(0.50-1.99)	1.091	(0.98-1.27)
80-84	0.925	(0.44-1.93)	1.228	(1.05-1.42)

© 2023 Chien et al. *JAMA Network Open.*

eTable 26B. Age-specific early-stage lung SCLC incidence rate ratios and their 95% confidence intervals comparing sex (1), smoking status (2), periods (3).

(1) sex

Age	Never-smoking Female/Male, 2011-2019		Ever-smoking Female/Male, 2011-2019	
	**IRR**	**95% CI**	**IRR**	**95% CI**
40-44	Inf		0.000	(0.00-0.00)
45-49	NA		NA	NA
50-54	Inf		0.000	(0.00-0.00)
55-59	0.455	(0.00-0.97)	2.872	(0.00-14.37)
60-64	Inf		0.000	(0.00-0.00)
65-69	Inf		0.000	(0.00-0.00)
70-74	1.280	(0.00-3.28)	0.000	(0.00-0.00)
75-79	0.487	(0.07-2.51)	0.000	(0.00-0.00)
80-84	0.352	(0.08-1.13)	0.000	(0.00-0.00)
**Age**	**Never-smoking Female/Male, 2011-2015**		**Ever-smoking Female/Male, 2011-2015**	
	**IRR**	**95% CI**	**IRR**	**95% CI**
40-64	1.751	(0.07-2.65)	1.051	(0.00-5.50)
65-84	0.454	(0.19-1.53)	0.000	(0.00-0.00)
**Age**	**Never-smoking Female/Male, 2016-2019**		**Ever-smoking Female/Male, 2016-2019**	
	**IRR**	**95% CI**	**IRR**	**95% CI**
40-64	1.921	(0.00-3.51)	0.000	(0.00-0.00)
65-84	0.786	(0.17-2.36)	0.000	(0.00-0.00)

© 2023 Chien et al. *JAMA Network Open.*

(2) smoking status

Age	Ever/never smoking Female, 2011-2019		Ever/never smoking Male, 2011-2019	
	IRR	95% CI	IRR	95% CI
40-44	0.000	(0.00-0.00)	Inf	NA
45-49	NA	NA	0.000	(0.00-0.00)
50-54	0.000	(0.00-0.00)	Inf	NA
55-59	25.651	(0-102.72)	4.065	(0.41-8.49)
60-64	0.000	(0.00-0.00)	Inf	NA
65-69	0.000	(0.00-0.00)	Inf	NA
70-74	0.000	(0.00-0.00)	13.845	(3.17-25.79)
75-79	0.000	(0.00-0.00)	5.702	(1.82-25.29)
80-84	0.000	(0.00-0.00)	5.110	(2.28-13.80)
**Age**	**Ever/never smoking Female, 2011-2015**		**Ever/never smoking Male, 2011-2015**	
	**IRR**	**95% CI**	**IRR**	**95% CI**
40-64	4.373	(0.00-27.53)	7.283	(1.62-11.89)
65-84	0.000	(0.00-0.00)	5.317	(2.86-16.00)
**Age**	**Ever/never smoking Female, 2016-2019**		**Ever/never smoking Male, 2016-2019**	
	**IRR**	**95% CI**	**IRR**	**95% CI**
40-64	0.000	(0.00-0.00)	15.280	(3.48-22.68)
65-84	0.000	(0.00-0.00)	14.480	(5.75-53.13)

© 2023 Chien et al. *JAMA Network Open.*

(3) periods

Age	Never-smoking Female, 2016-2019/2011-2015		Never-smoking Male, 2016-2019/2011-2015	
	**IRR**	**95% CI**	**IRR**	**95% CI**
40-64	1.157	(0.00-4.77)	1.055	(0.00-3.85)
65-84	0.590	(0.18-2.47)	0.341	(0.00-1.31)
**Age**	**Ever-smoking Female, 2016-2019/2011-2015**		**Ever-smoking Male, 2016-2019/2011-2015**	
	**IRR**	**95% CI**	**IRR**	**95% CI**
40-64	0.000	(0.00-0.00)	2.213	(1.13-6.33)
65-84	NA	NA	0.929	(0.56-1.54)

© 2023 Chien et al. *JAMA Network Open.*

eTable 26C. Age-specific late-stage 3&4 lung SCLC incidence rate ratios and their 95% confidence intervals comparing sex (1), smoking status (2), periods (3).

(1) sex

Age	Never-smoking Female/Male, 2011-2019		Ever-smoking Female/Male, 2011-2019	
	IRR	95% CI	IRR	95% CI
40-44	1.202	(0.02-4.46)	0.520	(0.10-1.12)
45-49	0.265	(0.11-0.55)	0.879	(0.44-1.62)
50-54	0.436	(0.27-0.91)	0.811	(0.44-1.25)
55-59	0.365	(0.25-0.60)	1.205	(0.83-1.62)
60-64	0.278	(0.19-0.37)	1.161	(0.94-1.41)
65-69	0.202	(0.14-0.29)	1.556	(1.12-1.97)
70-74	0.205	(0.12-0.29)	1.338	(0.96-1.66)
75-79	0.143	(0.09-0.22)	0.832	(0.58-1.12)
80-84	0.179	(0.13-0.26)	0.953	(0.68-1.28)

Age	Never-smoking Female/Male, 2011-2015		Ever-smoking Female/Male, 2011-2015	
	IRR	95% CI	IRR	95% CI
40-44	1.270	(0.00-3.05)	1.005	(0.13-2.53)
45-49	0.220	(0.04-0.61)	0.922	(0.46-1.54)
50-54	0.351	(0.15-0.68)	0.616	(0.28-1.22)
55-59	0.388	(0.18-0.74)	1.225	(0.90-1.71)
60-64	0.250	(0.12-0.42)	0.980	(0.56-1.36)
65-69	0.212	(0.11-0.36)	1.575	(1.08-2.20)

© 2023 Chien et al. *JAMA Network Open.*

70-74	0.207	(0.11-0.36)	1.189	(0.85-1.65)
75-79	0.106	(0.06-0.17)	0.848	(0.56-1.19)
80-84	0.160	(0.09-0.25)	1.108	(0.69-1.59)

Age	Never-smoking Female/Male, 2016-2019		Ever-smoking Female/Male, 2016-2019	
	**IRR**	**95% CI**	**IRR**	**95% CI**
40-44	1.098	(0.00-2.26)	0.168	(0.00-0.80)
45-49	0.328	(0.06-1.99)	0.815	(0.24-1.28)
50-54	0.554	(0.24-1.20)	1.035	(0.53-1.59)
55-59	0.341	(0.15-0.71)	1.192	(0.68-1.70)
60-64	0.308	(0.17-0.49)	1.366	(0.95-1.87)
65-69	0.196	(0.11-0.29)	1.554	(1.02-2.21)
70-74	0.208	(0.11-0.37)	1.553	(1.02-2.21)
75-79	0.201	(0.10-0.31)	0.791	(0.48-1.14)
80-84	0.221	(0.13-0.38)	0.757	(0.38-1.19)

(2) smoking status

Age	Ever/never smoking Female, 2011-2019		Ever/never smoking Male, 2011-2019	
	**IRR**	**95% CI**	**IRR**	**95% CI**
40-44	12.534	(2.96-53.17)	28.980	(12.17-71.34)
45-49	27.426	(13.29-77.46)	8.262	(5.05-15.86)
50-54	17.722	(10.49-31.85)	9.521	(6.79-16.44)
55-59	39.718	(25.40-63.27)	12.029	(9.22-17.14)

© 2023 Chien et al. *JAMA Network Open.*

60-64	43.516	(31.85-64.45)	10.419	(8.42-14.59)
65-69	63.580	(42.56-82.32)	8.275	(6.85-10.47)
70-74	85.568	(57.06-142.54)	13.112	(10.32-17.47)
75-79	42.292	(25.79-67.46)	7.257	(6.25-9.09)
80-84	29.135	(17.21-42.46)	5.480	(4.49-6.78)

**Age**	**Ever/never smoking Female, 2011-2015**		**Ever/never smoking Male, 2011-2015**	
	**IRR**	**95% CI**	**IRR**	**95% CI**
40-44	16.622	(1.93-74.92)	21.014	(4.77-30.15)
45-49	33.035	(11.87-139.04)	7.892	(3.71-21.60)
50-54	14.513	(6.92-33.28)	8.267	(4.74-17.66)
55-59	36.488	(23.35-70.29)	11.548	(7.97-21.38)
60-64	41.769	(21.25-84.73)	10.631	(7.94-15.81)
65-69	60.789	(39.07-111.75)	8.177	(5.93-10.65)
70-74	79.804	(41.51-154.30)	13.869	(10.27-20.51)
75-79	47.077	(25.09-90.92)	5.894	(4.73-7.78)
80-84	28.478	(15.65-54.84)	4.104	(3.24-5.49)

**Age**	**Ever/never smoking Female, 2016-2019**		**Ever/never smoking Male, 2016-2019**	
	**IRR**	**95% CI**	**IRR**	**95% CI**
40-44	6.309	(0.00-33.29)	41.297	(9.56-54.28)
45-49	21.835	(5.38-82.31)	8.797	(4.24-30.19)
50-54	21.139	(8.44-55.66)	11.321	(6.78-24.03)
55-59	44.162	(20.57-85.72)	12.625	(7.75-20.18)

© 2023 Chien et al. *JAMA Network Open.*

60-64	45.117	(25.41-76.31)	10.169	(7.08-13.99)
65-69	67.004	(39.59-115.03)	8.433	(6.43-11.77)
70-74	95.996	(50.15-181.72)	12.881	(10.08-18.15)
75-79	36.621	(20.48-78.95)	9.290	(7.18-12.45)
80-84	29.315	(11.10-53.16)	8.543	(6.49-12.81)

(3) periods

© 2023 Chien et al. *JAMA Network Open.*

Age	Never-smoking Female, 2016-2019/2011-2015		Never-smoking Male, 2016-2019/2011-2015	
	IRR	95% CI	IRR	95% CI
40-44	0.806	(0.00-4.27)	0.932	(0.00-3.49)
45-49	1.432	(0.37-9.00)	0.961	(0.35-1.98)
50-54	1.338	(0.43-2.84)	0.847	(0.34-1.99)
55-59	0.944	(0.50-2.06)	1.074	(0.44-2.34)
60-64	1.205	(0.71-1.83)	0.976	(0.58-1.56)
65-69	0.996	(0.54-2.22)	1.079	(0.73-1.88)
70-74	1.247	(0.56-2.26)	1.236	(0.85-1.99)
75-79	1.303	(0.64-2.43)	0.689	(0.51-1.04)
80-84	0.826	(0.44-1.37)	0.598	(0.40-0.87)
**Age**	**Ever-smoking Female, 2016-2019/2011-2015**		**Ever-smoking Male, 2016-2019/2011-2015**	
	**IRR**	**95% CI**	**IRR**	**95% CI**
40-44	0.306	(0.00-3.06)	1.831	(1.13-3.05)
45-49	0.947	(0.19-2.91)	1.071	(0.78-1.44)
50-54	1.949	(0.75-5.52)	1.159	(0.90-1.42)
55-59	1.142	(0.57-1.89)	1.174	(0.96-1.39)
60-64	1.301	(0.71-1.88)	0.934	(0.80-1.06)
65-69	1.098	(0.65-1.67)	1.113	(0.99-1.24)
70-74	1.499	(0.81-2.46)	1.148	(1.00-1.29)
75-79	1.014	(0.45-1.85)	1.087	(0.97-1.26)
80-84	0.850	(0.39-1.69)	1.245	(1.06-1.45)

© 2023 Chien et al. *JAMA Network Open.*

eTable 27. Histology codes for lung SCLC that appeared in the TCR for each year from 2011 to 2019.

2011	2012	2013	2014	2015	2016	2017	2018	2019
80413	80413	80413	80413	80413	80413	80413	80413	80413
80453	80453	80453	80453	80433	80433	80453	80453	80453
80443	80443	80443		80453	80453	80423		
				80423	80423			
				80443	80443			

© 2023 Chien et al. *JAMA Network Open.*

eTable 28. Age- and sex-specific number of patients with invasive lung cancer diagnosed for each year from 2011 to 2019 according to the TCR.

Year	2011		2012		2013		2014		2015		2016		2017		2018		2019	
Age \ Sex	M	F	M	F	M	F	M	F	M	F	M	F	M	F	M	F	M	F
40	23	13	22	20	18	16	23	18	25	18	16	20	15	21	24	37	30	42
41	19	21	14	23	26	19	18	25	13	25	27	12	31	21	27	33	24	45
42	28	18	21	18	18	24	21	15	23	23	16	27	27	22	35	35	22	43
43	39	18	29	24	41	23	32	30	23	33	19	32	27	37	32	28	41	42
44	32	36	44	28	34	30	45	26	34	37	24	36	40	35	38	46	30	53
45	32	25	38	39	36	55	33	43	31	35	42	42	48	41	53	52	41	43
46	42	48	42	50	42	30	43	42	40	43	57	34	38	41	38	43	42	57
47	43	46	54	41	47	46	42	53	53	43	42	56	56	51	49	71	51	66
48	53	54	58	29	52	50	64	55	58	58	69	56	66	54	62	62	72	79
49	44	50	67	58	73	59	84	68	78	64	76	57	74	86	70	70	57	74
50	70	69	74	65	74	67	65	64	71	65	81	69	64	73	101	91	84	102
51	72	66	84	67	103	69	85	82	75	68	81	95	87	87	98	84	96	97
52	70	79	97	75	95	92	93	82	102	84	79	97	86	89	87	112	96	113
53	86	66	96	83	81	74	87	95	99	85	116	92	101	105	105	108	97	114
54	74	73	87	64	95	82	113	94	125	108	121	115	127	94	116	111	128	124
55	108	77	103	76	109	74	124	97	115	90	118	114	138	120	142	129	144	143
56	111	76	100	107	112	87	126	106	132	134	129	106	143	117	129	133	162	156
57	117	86	125	82	135	100	130	100	134	98	137	123	155	135	180	157	177	141
58	126	88	135	89	131	119	160	128	138	96	144	127	161	145	185	156	163	159
59	140	88	151	103	157	99	148	133	167	133	153	137	160	158	194	133	189	160
60	146	98	173	121	172	107	147	128	174	133	201	137	182	148	179	158	239	180
61	139	105	174	113	165	122	168	130	186	146	191	161	183	172	221	155	198	208
62	139	101	127	117	166	128	150	151	155	159	193	160	200	145	231	169	235	183
63	126	87	144	118	180	116	169	125	206	151	209	149	220	152	204	190	244	181
64	124	82	143	106	187	115	178	134	204	171	187	159	186	178	230	199	242	197
65	117	70	132	92	148	111	178	130	178	136	204	168	207	178	243	186	234	192
66	98	73	115	67	122	102	167	114	207	131	215	160	230	179	227	180	260	188
67	149	76	131	77	117	70	167	125	188	115	203	159	226	173	272	208	276	196
68	142	93	154	80	119	72	125	105	170	86	200	140	213	177	203	169	309	192
69	168	95	153	114	145	80	136	74	151	76	154	132	186	116	235	149	236	173
70	186	107	178	111	195	91	190	87	112	89	167	109	177	118	182	152	229	168

© 2023 Chien et al. *JAMA Network Open.*

Year	2011		2012		2013		2014		2015		2016		2017		2018		2019	
Age \ Sex	M	F	M	F	M	F	M	F	M	F	M	F	M	F	M	F	M	F
71	191	82	175	113	176	114	186	110	184	107	138	81	139	98	179	135	195	145
72	203	112	190	96	175	116	168	106	186	105	162	127	140	75	169	97	180	134
73	185	84	186	107	181	122	181	116	188	110	219	121	176	130	158	102	161	114
74	204	83	185	126	189	110	184	114	209	118	195	122	210	133	184	123	147	121
75	164	102	182	118	175	104	197	114	188	121	203	125	187	130	193	122	196	124
76	213	92	187	97	159	96	173	108	201	130	189	122	190	139	179	120	216	116
77	176	105	174	110	168	92	172	99	158	107	170	111	185	146	167	123	174	109
78	185	69	207	101	175	98	172	101	185	119	181	99	181	130	187	130	157	138
79	201	83	175	96	163	101	175	94	179	117	150	104	161	107	179	118	171	138
80	194	76	160	98	150	90	161	79	143	103	164	92	167	117	151	119	134	109
81	217	76	170	86	176	59	146	98	142	92	150	83	139	94	161	90	135	112
82	187	87	177	83	176	91	167	64	138	84	156	107	163	93	110	110	128	95
83	163	73	143	87	175	77	147	82	158	87	142	74	130	88	124	107	118	91
84	139	70	178	58	137	55	161	94	181	88	114	65	115	70	117	90	108	89

© 2023 Chien et al. *JAMA Network Open.*

eTable 29A. Age-, sex-, and calendar year-specific percentages of patients with late-stage (stage2—4) lung cancer among those with all stage (1—4) of invasive lung cancer, estimated using the TCRLF for each year from 2011 to 2019.

Year	2011		2012		2013		2014		2015		2016		2017		2018		2019	
Sex / Age	M	F	M	F	M	F	M	F	M	F	M	F	M	F	M	F	M	F
40-44	0.931	0.820	0.873	0.768	0.865	0.657	0.864	0.673	0.726	0.557	0.747	0.492	0.716	0.448	0.682	0.503	0.718	0.470
45-49	0.894	0.784	0.796	0.689	0.860	0.730	0.822	0.664	0.764	0.600	0.776	0.576	0.785	0.641	0.748	0.526	0.709	0.468
50-54	0.896	0.785	0.859	0.720	0.811	0.666	0.834	0.654	0.785	0.645	0.761	0.583	0.774	0.529	0.750	0.510	0.713	0.468
55-59	0.869	0.788	0.859	0.733	0.845	0.731	0.816	0.740	0.816	0.609	0.771	0.599	0.781	0.561	0.757	0.521	0.765	0.506
60-64	0.869	0.759	0.866	0.724	0.823	0.722	0.825	0.739	0.805	0.681	0.803	0.624	0.780	0.597	0.775	0.591	0.764	0.563
65-69	0.897	0.802	0.842	0.763	0.857	0.758	0.852	0.688	0.827	0.692	0.821	0.646	0.773	0.597	0.785	0.625	0.776	0.571
70-74	0.886	0.823	0.858	0.813	0.873	0.799	0.866	0.757	0.842	0.727	0.826	0.733	0.826	0.718	0.813	0.690	0.784	0.688
75-79	0.911	0.857	0.891	0.834	0.892	0.819	0.855	0.817	0.873	0.812	0.870	0.774	0.847	0.740	0.858	0.717	0.838	0.738
80-84	0.919	0.900	0.910	0.901	0.889	0.867	0.891	0.903	0.902	0.895	0.889	0.880	0.873	0.824	0.904	0.841	0.878	0.833

© 2023 Chien et al. *JAMA Network Open.*

eTable 29B. Age-, sex- and calendar year-specific percentages of ever-smokers among corresponding patients with late-stage (2—4) lung cancer in Taiwan.

Year	2011		2012		2013		2014		2015		2016		2017		2018		2019	
Age \ Sex	M	F	M	F	M	F	M	F	M	F	M	F	M	F	M	F	M	F
40-44	0.802	0.086	0.796	0.177	0.793	0.100	0.778	0.186	0.697	0.186	0.740	0.183	0.713	0.203	0.720	0.172	0.737	0.212
45-49	0.770	0.101	0.826	0.121	0.804	0.106	0.799	0.123	0.756	0.150	0.808	0.240	0.777	0.108	0.771	0.145	0.676	0.170
50-54	0.779	0.074	0.780	0.126	0.805	0.120	0.814	0.157	0.833	0.138	0.795	0.154	0.809	0.157	0.813	0.132	0.805	0.185
55-59	0.780	0.072	0.780	0.064	0.790	0.103	0.785	0.108	0.785	0.100	0.800	0.104	0.826	0.126	0.783	0.119	0.814	0.090
60-64	0.791	0.075	0.794	0.091	0.779	0.085	0.795	0.090	0.761	0.070	0.764	0.087	0.778	0.098	0.759	0.076	0.774	0.121
65-69	0.819	0.083	0.768	0.080	0.786	0.081	0.769	0.084	0.783	0.119	0.744	0.104	0.775	0.081	0.750	0.085	0.760	0.086
70-74	0.797	0.100	0.803	0.075	0.788	0.079	0.778	0.062	0.776	0.087	0.776	0.089	0.772	0.071	0.764	0.067	0.753	0.089
75-79	0.783	0.092	0.774	0.060	0.778	0.091	0.761	0.073	0.772	0.072	0.768	0.067	0.730	0.054	0.728	0.080	0.745	0.074
80-84	0.765	0.106	0.754	0.086	0.750	0.107	0.755	0.079	0.762	0.080	0.725	0.062	0.736	0.059	0.709	0.067	0.727	0.041

© 2023 Chien et al. *JAMA Network Open.*

eTable 29C. Age-, sex- and year-specific percentages of ever-smokers among corresponding patients with early-stage (1) lung cancer in Taiwan.

Year	2011		2012		2013		2014		2015		2016		2017		2018		2019	
Age \ Sex	M	F	M	F	M	F	M	F	M	F	M	F	M	F	M	F	M	F
40-44	0.750	0.056	0.600	0.115	0.278	0.054	0.444	0.028	0.548	0.088	0.360	0.016	0.421	0.095	0.319	0.103	0.375	0.079
45-49	0.650	0.044	0.633	0.062	0.485	0.032	0.477	0.073	0.362	0.052	0.623	0.101	0.441	0.097	0.446	0.080	0.480	0.055
50-54	0.694	0.083	0.559	0.053	0.538	0.073	0.606	0.043	0.596	0.043	0.542	0.084	0.584	0.050	0.467	0.045	0.507	0.067
55-59	0.681	0.012	0.556	0.035	0.526	0.066	0.517	0.064	0.570	0.052	0.574	0.034	0.613	0.049	0.560	0.042	0.538	0.058
60-64	0.646	0.037	0.708	0.026	0.576	0.089	0.652	0.042	0.586	0.013	0.554	0.043	0.632	0.029	0.526	0.046	0.564	0.050
65-69	0.778	0.053	0.768	0.063	0.667	0.040	0.604	0.074	0.662	0.031	0.545	0.027	0.553	0.035	0.534	0.047	0.567	0.028
70-74	0.694	0.080	0.643	0.031	0.635	0.029	0.649	0.058	0.659	0.051	0.539	0.028	0.706	0.034	0.539	0.061	0.568	0.024
75-79	0.704	0.036	0.726	0.027	0.628	0.014	0.625	0.037	0.735	0.061	0.692	0.078	0.608	0.057	0.627	0.043	0.574	0.058
80-84	0.661	0.032	0.729	0.031	0.676	0.095	0.701	0.129	0.714	0.054	0.697	0.067	0.750	0.029	0.736	0.028	0.582	0.014

© 2023 Chien et al. *JAMA Network Open.*

eTable 29D. Age-, sex-, and calendar year-specific percentages of patients with stage 3&4 lung cancer among those with all stages (1—4) invasive lung cancer, estimated using the TCRLF for each year from 2011 to 2019.

Year	2011		2012		2013		2014		2015		2016		2017		2018		2019	
Age \ Sex	M	F	M	F	M	F	M	F	M	F	M	F	M	F	M	F	M	F
40-44	0.863	0.800	0.841	0.723	0.789	0.620	0.780	0.655	0.690	0.527	0.667	0.459	0.687	0.418	0.642	0.458	0.697	0.447
45-49	0.843	0.746	0.759	0.646	0.802	0.670	0.784	0.612	0.720	0.563	0.726	0.525	0.742	0.595	0.710	0.498	0.659	0.429
50-54	0.854	0.743	0.816	0.690	0.767	0.628	0.780	0.627	0.721	0.608	0.728	0.550	0.721	0.487	0.711	0.478	0.678	0.437
55-59	0.818	0.749	0.805	0.690	0.792	0.700	0.768	0.689	0.772	0.585	0.735	0.568	0.748	0.523	0.711	0.473	0.726	0.475
60-64	0.815	0.730	0.819	0.681	0.772	0.687	0.767	0.690	0.762	0.640	0.755	0.602	0.733	0.554	0.723	0.554	0.719	0.515
65-69	0.849	0.755	0.794	0.739	0.801	0.701	0.792	0.644	0.767	0.652	0.773	0.603	0.739	0.558	0.729	0.578	0.723	0.517
70-74	0.825	0.784	0.796	0.773	0.822	0.763	0.816	0.720	0.771	0.680	0.772	0.691	0.788	0.688	0.755	0.656	0.733	0.646
75-79	0.849	0.811	0.833	0.797	0.837	0.781	0.794	0.786	0.815	0.781	0.813	0.748	0.799	0.707	0.807	0.685	0.798	0.694
80-84	0.866	0.866	0.853	0.878	0.850	0.828	0.851	0.881	0.848	0.869	0.842	0.860	0.822	0.803	0.846	0.812	0.832	0.781

© 2023 Chien et al. *JAMA Network Open.*

eTable 29E. Age-, sex- and year-specific percentages of ever-smokers among corresponding patients with stage 3&4 lung cancers in Taiwan.

Year	2011		2012		2013		2014		2015		2016		2017		2018		2019	
Age \ Sex	M	F	M	F	M	F	M	F	M	F	M	F	M	F	M	F	M	F
40-44	0.785	0.089	0.808	0.176	0.802	0.091	0.786	0.176	0.694	0.182	0.754	0.179	0.700	0.218	0.734	0.177	0.739	0.223
45-49	0.768	0.107	0.829	0.120	0.812	0.115	0.804	0.121	0.753	0.152	0.799	0.239	0.768	0.110	0.769	0.146	0.682	0.171
50-54	0.777	0.074	0.774	0.132	0.803	0.119	0.820	0.152	0.834	0.138	0.798	0.160	0.808	0.150	0.817	0.137	0.805	0.186
55-59	0.778	0.069	0.773	0.064	0.792	0.101	0.783	0.111	0.783	0.101	0.798	0.107	0.824	0.127	0.780	0.125	0.818	0.093
60-64	0.785	0.075	0.797	0.095	0.783	0.079	0.798	0.089	0.764	0.073	0.762	0.089	0.775	0.103	0.756	0.077	0.779	0.121
65-69	0.815	0.080	0.762	0.079	0.788	0.080	0.773	0.087	0.780	0.123	0.743	0.107	0.776	0.085	0.750	0.084	0.756	0.088
70-74	0.799	0.105	0.796	0.079	0.783	0.078	0.784	0.063	0.772	0.083	0.777	0.091	0.773	0.075	0.765	0.067	0.750	0.092
75-79	0.785	0.094	0.769	0.057	0.774	0.089	0.758	0.073	0.768	0.070	0.761	0.070	0.730	0.051	0.729	0.078	0.747	0.071
80-84	0.761	0.104	0.753	0.088	0.753	0.108	0.755	0.078	0.760	0.079	0.714	0.063	0.728	0.057	0.701	0.070	0.721	0.041

© 2023 Chien et al. *JAMA Network Open.*

eTable 30A. Estimated age- and sex-specific number of patients with late-stage lung cancer in the TCR by smoking status.

	Never-smokers																	
Year	2011		2012		2013		2014		2015		2016		2017		2018		2019	
Age \ Sex	M	F	M	F	M	F	M	F	M	F	M	F	M	F	M	F	M	F
40-44	26.036	79.408	23.139	71.391	24.545	66.267	26.677	62.448	25.914	61.712	19.844	51.008	28.809	48.510	29.795	74.487	27.787	83.326
45-49	43.978	157.135	35.851	131.357	42.111	156.618	43.961	151.908	48.556	123.930	42.690	107.258	49.344	156.027	46.631	134.034	60.353	123.812
50-54	73.809	256.563	82.900	222.679	70.724	224.892	68.873	229.677	61.760	228.048	74.466	230.694	68.634	199.746	71.158	223.972	69.527	209.913
55-59	115.039	303.248	115.893	313.852	114.246	314.159	120.909	372.060	120.425	301.827	105.075	325.635	102.804	330.577	136.280	324.894	119.089	349.433
60-64	122.440	331.989	135.741	378.166	158.176	388.402	137.070	449.377	178.090	480.840	186.346	436.419	168.115	427.842	198.534	475.204	199.911	469.594
65-69	109.628	299.239	133.880	302.007	119.174	303.256	151.759	345.531	160.637	331.788	205.435	439.843	184.748	451.655	231.185	509.868	245.166	491.068
70-74	174.302	346.529	154.577	415.871	169.509	407.114	175.004	378.210	165.921	351.026	163.049	373.950	158.535	369.533	167.061	392.235	176.475	427.460
75-79	185.943	350.906	186.464	409.509	166.493	365.574	181.815	390.978	181.452	447.396	180.010	404.954	206.471	456.790	211.023	404.577	195.633	427.275
80-84	194.386	307.504	185.597	339.152	181.217	288.080	170.542	346.673	163.754	374.180	177.729	347.666	164.642	358.394	174.326	404.897	149.365	396.291

	Ever-smokers																	
Year	2011		2012		2013		2014		2015		2016		2017		2018		2019	
Age \ Sex	M	F	M	F	M	F	M	F	M	F	M	F	M	F	M	F	M	F
40-44	105.277	7.512	90.353	15.377	93.913	7.363	93.369	14.243	59.714	14.075	56.399	11.451	71.489	12.386	76.616	15.518	77.804	22.434
45-49	147.325	17.705	170.292	18.155	172.765	18.490	174.796	21.396	150.084	21.870	179.296	33.929	172.155	18.946	156.850	22.699	126.193	25.397

© 2023 Chien et al. *JAMA Network Open.*

	**Ever-smokers**																	
Year	2011		2012		2013		2014		2015		2016		2017		2018		2019	
Sex / Age	M	F	M	F	M	F	M	F	M	F	M	F	M	F	M	F	M	F
50-54	259.419	20.568	293.424	32.117	292.689	30.763	300.638	42.858	308.801	36.402	289.230	42.136	291.432	37.187	309.092	34.129	287.887	47.708
55-59	408.166	23.658	411.584	21.350	430.038	36.123	440.767	45.033	439.071	33.653	420.298	37.739	488.050	47.833	491.884	43.963	520.060	34.726
60-64	463.045	26.947	523.098	38.034	557.486	36.207	532.804	44.183	566.849	36.411	601.790	41.819	588.953	46.693	626.658	39.247	685.251	64.475
65-69	495.021	26.999	443.188	26.261	438.826	26.601	506.606	31.610	578.292	44.748	596.316	50.793	636.602	40.075	694.677	47.503	775.075	46.206
70-74	684.511	38.629	629.931	33.903	630.105	34.863	612.512	25.141	573.904	33.325	564.313	36.510	536.937	28.426	542.096	28.017	538.592	41.543
75-79	669.395	35.666	637.587	26.038	582.726	36.557	578.172	30.711	614.054	34.691	597.251	29.166	559.097	25.941	565.155	35.230	570.498	33.911
80-84	632.734	36.415	567.549	31.875	542.338	34.619	526.169	29.863	523.497	32.321	467.976	22.857	458.465	22.400	424.838	29.285	397.417	16.863

© 2023 Chien et al. *JAMA Network Open.*

eTable 30B. Estimated age- and sex-specific number of patients with early-stage lung cancer in the TCR by smoking status.

	Never-smokers																	
Year	2011		2012		2013		2014		2015		2016		2017		2018		2019	
Age \ Sex	M	F	M	F	M	F	M	F	M	F	M	F	M	F	M	F	M	F
40-44	2.422	18.020	6.603	23.205	13.391	36.296	10.530	36.273	14.619	54.932	16.485	63.483	22.985	68.000	33.763	79.788	25.880	109.826
45-49	7.944	46.019	19.417	63.335	18.094	62.799	24.695	81.279	39.143	92.138	24.137	93.327	33.840	88.541	37.949	129.924	39.756	160.473
50-54	11.847	69.546	27.179	93.927	39.122	119.030	28.981	138.273	41.007	139.267	52.345	178.821	43.636	200.618	67.600	236.631	70.752	272.819
55-59	25.171	87.020	38.455	117.561	47.234	120.278	61.057	137.463	54.365	204.285	66.247	235.332	64.381	282.072	88.892	324.799	90.555	352.914
60-64	31.304	109.840	29.797	154.648	65.379	148.821	49.529	167.171	74.508	239.664	85.950	275.251	78.651	311.161	113.593	340.250	119.037	394.287
65-69	15.411	76.512	25.075	95.373	31.000	100.978	45.422	158.203	52.403	162.198	79.204	261.111	107.547	319.775	118.380	319.040	127.520	392.339
70-74	33.731	76.214	46.167	100.001	42.526	107.789	42.684	122.086	47.471	137.311	70.826	145.414	43.096	150.698	74.994	177.214	85.015	207.877
75-79	24.745	62.128	27.641	84.116	33.752	87.651	48.380	90.818	30.572	105.130	35.695	116.951	54.265	159.627	48.035	165.710	63.062	154.241
80-84	24.705	36.853	20.299	39.692	29.299	44.606	25.459	35.243	21.357	44.931	24.332	47.112	22.723	78.886	16.862	79.545	31.852	81.711

	Ever-smokers																	
Year	2011		2012		2013		2014		2015		2016		2017		2018		2019	
Age \ Sex	M	F	M	F	M	F	M	F	M	F	M	F	M	F	M	F	M	F
40-44	7.265	1.060	9.905	3.027	5.150	2.074	8.424	1.036	17.752	5.282	9.273	1.058	16.716	7.104	15.826	9.206	15.528	9.414
45-49	14.753	2.140	33.440	4.153	17.030	2.093	22.548	6.417	22.217	5.063	39.878	10.486	26.662	9.486	30.570	11.343	36.698	9.318

© 2023 Chien et al. *JAMA Network Open.*

	Ever-smokers																	
Year	2011		2012		2013		2014		2015		2016		2017		2018		2019	
Age \ Sex	M	F	M	F	M	F	M	F	M	F	M	F	M	F	M	F	M	F
50-54	26.925	6.322	34.496	5.277	45.466	9.315	44.507	6.191	60.432	6.283	61.959	16.349	61.298	10.449	59.150	11.268	72.833	19.561
55-59	53.625	1.074	48.068	4.236	52.482	8.441	65.268	9.444	72.139	11.236	89.381	8.294	101.764	14.518	112.945	14.344	105.296	21.927
60-64	57.211	4.225	72.365	4.152	88.959	14.570	92.597	7.268	105.553	3.086	106.914	12.511	135.280	9.304	126.215	16.299	153.801	20.643
65-69	53.939	4.251	82.858	6.358	62.000	4.164	69.214	12.656	102.668	5.266	95.045	7.253	133.103	11.495	135.757	15.588	167.239	11.387
70-74	76.457	6.627	83.325	3.226	73.860	3.234	78.801	7.563	91.704	7.337	82.812	4.125	103.431	5.344	87.850	11.535	111.918	5.120
75-79	58.917	2.301	73.308	2.337	57.029	1.217	80.633	3.493	84.922	6.783	80.044	9.930	84.166	9.642	80.787	7.484	84.807	9.574
80-84	48.174	1.228	54.555	1.280	61.146	4.695	59.830	5.221	53.393	2.568	55.963	3.365	68.169	2.320	46.974	2.273	44.366	1.135

© 2023 Chien et al. *JAMA Network Open.*

eTable 30C. Estimated age- and sex-specific number of patients with stage 3&4 lung cancer in the TCR by smoking status.

	Never-smokers																	
Year	2011		2012		2013		2014		2015		2016		2017		2018		2019	
Age \ Sex	M	F	M	F	M	F	M	F	M	F	M	F	M	F	M	F	M	F
40-44	26.144	77.286	20.989	67.366	21.417	63.165	23.242	61.450	24.888	58.609	16.738	47.885	28.836	44.435	26.652	67.399	26.735	78.107
45-49	41.822	148.709	33.708	123.305	37.712	142.229	40.859	140.436	46.249	115.977	41.720	97.922	48.445	144.413	44.561	126.808	55.189	113.521
50-54	70.679	242.863	80.657	212.202	67.624	212.480	62.358	221.579	56.576	214.720	70.270	216.344	64.536	185.457	65.822	208.614	66.233	195.595
55-59	109.374	289.463	112.202	295.108	105.891	301.521	114.458	345.300	115.099	289.616	100.958	308.043	99.743	308.204	129.941	293.030	110.573	326.697
60-64	118.089	319.432	126.661	354.232	145.975	372.060	126.118	419.627	166.414	451.081	175.972	420.010	159.921	395.168	187.702	445.808	183.787	429.913
65-69	105.869	282.451	129.344	292.577	110.302	280.751	138.755	322.563	151.176	310.903	193.515	409.035	175.868	420.612	215.401	471.994	231.610	444.035
70-74	161.030	328.489	148.479	393.713	163.502	389.174	160.524	359.814	154.472	329.592	151.775	351.381	150.559	352.669	154.995	372.620	166.895	399.701
75-79	171.384	331.420	177.758	392.154	158.805	348.963	170.655	375.792	172.201	431.436	173.549	390.202	195.164	437.129	197.851	386.955	184.481	403.377
80-84	186.137	296.284	174.121	330.175	171.002	274.854	163.109	338.972	155.033	363.250	174.792	339.009	159.737	349.828	167.547	389.445	144.575	371.597

	Ever-smokers																	
Year	2011		2012		2013		2014		2015		2016		2017		2018		2019	
Age \ Sex	M	F	M	F	M	F	M	F	M	F	M	F	M	F	M	F	M	F
40-44	95.482	7.514	88.376	14.357	86.740	6.316	85.220	13.168	56.563	13.024	51.262	10.410	67.284	12.401	73.560	14.517	75.750	22.469
45-49	138.673	17.756	162.921	16.862	162.701	18.457	167.627	19.296	140.951	20.710	165.811	30.807	160.748	17.912	148.538	21.677	118.105	23.341

© 2023 Chien et al. *JAMA Network Open.*

	Ever-smokers																	
Year	2011		2012		2013		2014		2015		2016		2017		2018		2019	
Sex / Age	M	F	M	F	M	F	M	F	M	F	M	F	M	F	M	F	M	F
50-54	246.831	19.516	276.851	32.152	275.948	28.685	283.347	39.686	283.968	34.355	277.838	41.158	270.840	32.852	294.525	33.164	273.620	44.647
55-59	383.362	21.521	381.917	20.315	404.115	33.990	413.809	43.028	414.571	32.648	399.347	36.749	466.190	44.637	460.121	42.015	495.934	33.647
60-64	431.136	25.900	496.733	37.058	525.289	31.735	496.729	41.098	538.397	35.442	564.209	40.819	551.453	45.471	581.988	37.062	648.266	58.966
65-69	466.273	24.658	414.595	25.203	411.125	24.367	473.335	30.616	534.675	43.789	560.526	48.824	608.861	39.026	645.081	43.293	719.093	42.902
70-74	638.368	38.380	578.836	33.941	589.774	32.713	581.168	23.988	523.614	29.963	528.314	35.359	513.060	28.533	503.733	26.775	501.835	40.629
75-79	625.610	34.403	592.527	23.695	544.476	34.284	535.124	29.637	570.415	32.358	552.743	29.237	527.407	23.659	532.861	32.908	545.219	30.679
80-84	593.555	34.544	531.810	31.748	520.898	33.278	502.588	28.572	490.938	31.100	436.342	22.922	427.233	21.089	393.540	29.465	373.487	15.735

© 2023 Chien et al. *JAMA Network Open.*

eTable 31. Number of patients with invasive lung cancer in TCR, TCRLF, having known smoking status (ever-smoker or never-smoker), and having a known number of pack-years smoked and a known number of years since smoking quitting, for 2011—2019.

Year	TCR	TCRLF	Smoking status known	Pack-year and quit time known
2011	9884	9263	8493	3283
2012	10352	9851	8970	3294
2013	10421	10008	9096	3329
2014	10990	10626	9588	3415
2015	11425	11136	10100	3563
2016	11811	11481	10516	3589
2017	12362	12008	11023	3658
2018	13017	12699	11712	3673
2019	13508	13209	12172	3716
Sum	103770	100281	91670	31520

© 2023 Chien et al. *JAMA Network Open.*

eTable 32A. Late-stage lung cancer age-specific incidence rates for each single year and for the period 2011—2019 by sex and smoking status: never-smoking female (1), never-smoking male (2), ever-smoking female (3), ever-smoking male (4).

(1) never-smoking female

Age	Never-smoking Female														
	2011	2012	2013	2014	2015	2016	2017	2018	2019	2011-2019		2011-2015		2016-2019	
	rate									rate	95% Cl	rate	95% Cl	rate	95% Cl
40-44	9.421	8.500	7.921	7.534	7.500	6.124	5.691	8.553	9.302	7.852	(7.27-8.46)	8.181	(7.34-9.09)	7.454	(6.49-8.40)
45-49	18.095	15.245	18.425	18.007	14.743	12.832	18.743	16.175	15.090	16.383	(15.46-17.32)	16.907	(15.64-18.17)	15.709	(14.32-17.09)
50-54	31.186	26.659	26.482	26.813	26.573	26.870	23.453	26.657	25.167	26.637	(25.50-27.69)	27.519	(25.87-29.04)	25.538	(23.82-27.18)
55-59	41.224	41.813	40.982	47.432	37.801	40.292	40.290	38.942	41.485	41.110	(39.66-42.63)	41.848	(39.92-43.96)	40.255	(38.15-42.36)
60-64	64.628	66.015	62.824	68.570	69.880	61.339	59.028	64.290	62.175	64.189	(62.23-66.23)	66.556	(63.55-69.22)	61.727	(58.93-64.69)
65-69	86.653	86.953	83.671	88.680	76.739	89.593	82.446	86.175	78.183	84.008	(81.42-86.83)	84.270	(79.96-88.17)	83.791	(80.05-87.49)
70-74	108.938	127.823	123.229	113.685	107.563	117.719	115.489	117.257	118.620	116.746	(112.90-120.76)	116.285	(110.79-121.63)	117.311	(111.67-123.22)
75-79	148.804	168.411	145.276	150.257	166.024	145.410	160.164	139.380	145.774	151.948	(147.18-157.06)	155.864	(149.17-163.05)	147.646	(140.71-154.57)
80-84	194.706	206.119	168.988	196.464	204.559	184.324	183.910	200.212	188.631	191.904	(185.18-198.28)	194.244	(184.67-204.37)	189.397	(179.82-199.17)

© 2023 Chien et al. *JAMA Network Open.*

(2) never-smoking male

Age	Never-smoking Male											
	2011	2012	2013	2014	2015	2016	2017	2018	2019	2011-2019	2011-2015	2016-2019
	rate									rate 95% CI	rate 95% CI	rate 95% CI
40-44	7.666	6.451	6.508	6.775	6.310	4.546	6.146	5.940	5.148	6.077 (5.31-6.87)	6.719 (5.59-7.87)	5.457 (4.47-6.47)
45-49	12.401	9.836	11.309	11.519	12.393	10.649	12.051	11.169	14.241	11.758 (10.55-12.82)	11.500 (9.92-12.87)	12.050 (10.41-13.74)
50-54	21.627	23.793	19.866	19.087	17.013	20.448	18.925	19.786	19.405	19.968 (18.48-21.53)	20.235 (18.14-22.38)	19.642 (17.24-21.87)
55-59	35.967	35.103	33.509	34.235	33.086	28.170	26.800	34.519	29.541	32.176 (30.28-34.08)	34.337 (31.61-37.00)	29.800 (26.89-32.68)
60-64	53.732	52.720	56.193	45.363	55.393	55.273	48.318	55.294	53.787	52.896 (50.21-55.63)	52.608 (48.62-56.60)	53.179 (49.23-57.14)
65-69	80.926	95.395	78.935	90.888	84.365	92.606	72.860	82.464	80.594	83.596 (79.28-87.58)	86.088 (79.71-92.58)	81.753 (76.04-87.17)
70-74	138.697	115.081	118.954	116.798	108.416	105.637	98.425	95.637	90.627	108.217 (102.72-113.65)	118.989 (110.87-126.88)	97.122 (89.66-104.69)
75-79	206.304	201.084	171.429	176.713	165.075	152.762	164.586	159.192	140.773	168.251 (160.38-176.46)	183.074 (170.67-195.41)	154.062 (143.94-164.72)
80-84	250.435	231.644	223.173	209.388	200.890	217.039	196.890	200.429	162.748	209.277 (198.48-219.92)	222.814 (208.51-236.87)	193.475 (178.94-209.14)

© 2023 Chien et al. *JAMA Network Open.*

(3) ever-smoking female

Age	Ever-smoking Female														
	2011	2012	2013	2014	2015	2016	2017	2018	2019	2011-2019		2011-2015		2016-2019	
	rate									rate	95% CI	rate	95% CI	rate	95% CI
40-44	11.052	22.764	10.972	21.477	21.438	17.275	18.307	22.509	31.720	19.789	(16.11-23.18)	17.505	(13.15-22.12)	22.583	(17.18-28.14)
45-49	30.049	30.994	31.935	37.164	38.052	59.255	33.162	39.838	44.931	38.313	(32.80-43.40)	33.610	(26.52-39.94)	44.306	(34.68-52.66)
50-54	46.354	71.905	68.335	95.209	81.429	95.056	85.330	80.102	113.824	81.743	(72.94-90.61)	72.711	(62.12-83.12)	93.465	(79.45-109.02)
55-59	71.747	65.546	112.274	141.581	107.706	123.803	160.559	150.971	123.065	116.413	(103.45-128.60)	99.405	(82.73-113.20)	139.663	(118.19-160.68)
60-64	146.427	186.651	165.824	192.274	152.011	170.086	187.797	155.935	252.582	180.082	(162.74-198.37)	169.026	(145.05-193.41)	191.954	(164.76-218.68)
65-69	255.672	260.013	266.072	310.901	420.588	447.387	337.930	397.974	393.292	346.474	(309.09-385.29)	303.549	(256.49-349.76)	393.580	(332.65-449.93)
70-74	397.125	358.334	382.556	289.609	414.980	496.996	410.380	415.160	616.331	412.721	(365.55-457.58)	368.473	(313.25-426.55)	484.466	(407.05-572.65)
75-79	494.593	368.233	526.650	452.312	523.151	452.866	420.171	601.615	618.527	491.338	(436.89-544.41)	472.405	(398.34-548.36)	518.722	(430.03-609.55)
80-84	754.015	666.168	736.185	648.569	718.051	524.018	530.976	717.791	429.134	641.046	(559.85-714.80)	704.849	(597.73-806.93)	550.966	(440.05-663.09)

© 2023 Chien et al. *JAMA Network Open.*

(4) ever-smoking male

Age	Ever-smoking Male														
	2011	2012	2013	2014	2015	2016	2017	2018	2019	2011-2019		2011-2015		2016-2019	
	rate									rate	95% CI	rate	95% CI	rate	95% CI
40-44	18.258	16.497	18.089	19.077	12.940	12.712	16.588	18.374	19.194	16.893	(15.66-18.08)	17.061	(15.46-18.69)	16.636	(14.79-18.50)
45-49	25.310	30.240	31.879	33.386	29.606	36.636	36.489	34.535	28.989	31.735	(30.10-33.45)	29.999	(28.08-31.98)	34.284	(31.56-36.85)
50-54	49.298	55.149	54.361	55.625	57.342	54.053	55.216	59.623	56.276	55.208	(53.10-57.31)	54.377	(51.61-57.11)	56.269	(53.09-59.44)
55-59	93.694	93.846	97.344	98.738	97.831	93.666	108.396	108.791	115.442	100.925	(97.49-104.04)	96.311	(92.35-100.35)	106.588	(101.96-110.79)
60-64	163.866	168.319	168.565	154.167	158.342	164.662	160.383	169.850	184.288	165.937	(161.26-170.49)	162.397	(156.18-168.89)	169.848	(163.56-176.45)
65-69	261.064	237.744	230.504	253.378	267.027	248.774	244.553	254.038	274.933	253.378	(246.33-260.02)	250.473	(239.80-260.45)	256.085	(245.88-265.97)
70-74	452.231	422.337	431.766	432.774	430.503	455.352	450.753	456.053	442.339	440.915	(429.06-452.79)	434.075	(417.86-448.78)	451.116	(431.91-470.97)
75-79	578.829	581.085	549.550	557.655	600.621	590.550	563.021	584.408	611.228	579.211	(564.35-594.09)	573.572	(553.30-594.61)	586.970	(563.15-611.54)
80-84	729.213	686.056	700.288	733.385	789.707	761.684	792.782	767.208	739.952	740.383	(718.55-761.74)	725.314	(697.73-751.47)	765.788	(733.08-801.82)

© 2023 Chien et al. *JAMA Network Open.*

eTable 32B. Early-stage lung cancer age-specific incidence rates for each single year and for the period 2011—2019 by sex and smoking status: never-smoking female (1), never-smoking male (2), ever-smoking female (3), ever-smoking male (4).

(1) never-smoking female

Age	Never-smoking Female														
	2011	2012	2013	2014	2015	2016	2017	2018	2019	2011-2019		2011-2015		2016-2019	
	rate									rate	95% Cl	rate	95% Cl	rate	95% Cl
40-44	2.138	2.763	4.339	4.376	6.676	7.622	7.978	9.161	12.260	6.425	(5.88-6.94)	4.045	(3.48-4.70)	9.302	(8.23-10.31)
45-49	5.299	7.351	7.388	9.634	10.961	11.165	10.636	15.679	19.558	10.787	(10.02-11.57)	8.104	(7.20-8.96)	14.236	(12.93-15.55)
50-54	8.453	11.245	14.016	16.142	16.228	20.828	23.556	28.163	32.709	19.048	(18.00-19.97)	13.265	(12.20-14.35)	26.263	(24.49-27.89)
55-59	11.830	15.662	15.690	17.524	25.585	29.119	34.378	38.930	41.899	26.071	(24.95-27.21)	17.379	(16.14-18.69)	36.158	(34.10-38.18)
60-64	21.382	26.996	24.072	25.508	34.830	38.686	42.930	46.032	52.204	35.810	(34.24-37.25)	26.906	(24.97-28.74)	45.072	(42.69-47.56)
65-69	22.156	27.460	27.861	40.603	37.515	53.186	58.373	53.923	62.464	45.593	(43.65-47.73)	31.606	(28.87-34.31)	57.217	(53.93-60.30)
70-74	23.959	30.736	32.627	36.697	42.076	45.776	47.097	52.977	57.686	41.297	(38.88-43.67)	33.279	(30.56-35.95)	51.122	(47.20-54.93)
75-79	26.346	34.593	34.832	34.902	39.013	41.994	55.970	57.088	52.623	42.635	(39.96-45.24)	34.106	(30.79-37.21)	52.005	(47.69-56.23)
80-84	23.334	24.123	26.166	19.972	24.563	24.978	40.481	39.333	38.894	29.644	(27.00-32.22)	23.621	(20.30-26.86)	36.096	(32.05-40.21)

© 2023 Chien et al. *JAMA Network Open.*

(2) never-smoking male

Age	Never-smoking Male											
	2011	2012	2013	2014	2015	2016	2017	2018	2019	2011-2019	2011-2015	2016-2019
					**rate**					**rate 95% CI**	**rate 95% CI**	**rate 95% CI**
40-44	0.713	1.841	3.550	2.674	3.560	3.776	4.903	6.731	4.795	3.833 (3.21-4.49)	2.530 (1.81-3.24)	5.091 (4.11-6.06)
45-49	2.240	5.327	4.859	6.471	9.991	6.021	8.265	9.089	9.381	6.966 (6.11-7.73)	5.860 (4.77-6.97)	8.215 (6.72-9.51)
50-54	3.471	7.800	10.989	8.032	11.296	14.374	12.032	18.796	19.747	11.899 (10.73-13.07)	8.371 (7.06-9.66)	16.219 (14.12-18.27)
55-59	7.870	11.648	13.854	17.288	14.937	17.761	16.784	22.516	22.463	16.440 (15.08-17.87)	13.248 (11.53-14.93)	19.947 (17.69-22.32)
60-64	13.738	11.573	23.227	16.391	23.175	25.494	22.605	31.637	32.028	23.082 (21.38-24.91)	18.016 (15.82-20.13)	28.057 (25.36-31.15)
65-69	11.376	17.867	20.533	27.203	27.522	35.703	42.414	42.227	41.920	32.642 (30.20-35.08)	21.591 (18.36-24.86)	40.818 (36.79-44.62)
70-74	26.840	34.371	29.843	28.487	31.018	45.887	26.756	42.932	43.659	34.996 (31.72-38.05)	30.137 (26.23-34.17)	40.000 (35.34-44.54)
75-79	27.455	29.808	34.753	47.022	27.813	30.292	43.257	36.237	45.378	36.338 (32.75-39.90)	33.501 (28.82-38.35)	39.054 (33.80-44.29)
80-84	31.828	25.336	36.082	31.258	26.200	29.714	27.174	19.387	34.706	29.067 (25.33-32.97)	30.136 (24.64-35.58)	27.819 (22.37-33.11)

© 2023 Chien et al. *JAMA Network Open.*

(3) ever-smoking female

Age	Ever-smoking Female											
	2011	2012	2013	2014	2015	2016	2017	2018	2019	2011-2019	2011-2015	2016-2019
	rate									rate 95% CI	rate 95% CI	rate 95% CI
40-44	1.560	4.481	3.091	1.563	8.045	1.596	10.501	13.354	13.310	6.455 (4.27-8.55)	3.730 (1.79-5.68)	9.789 (6.21-13.16)
45-49	3.633	7.090	3.616	11.146	8.808	18.314	16.605	19.906	16.484	11.672 (8.68-14.47)	6.840 (3.79-9.64)	17.830 (12.29-23.26)
50-54	14.249	11.814	20.692	13.754	14.054	36.883	23.976	26.447	46.669	22.972 (18.42-27.76)	14.921 (9.83-20.11)	33.421 (24.94-42.34)
55-59	3.258	13.006	26.234	29.691	35.959	27.207	48.734	49.260	77.705	33.592 (26.22-40.59)	21.416 (13.68-27.99)	50.236 (39.11-63.75)
60-64	22.956	20.374	66.729	31.630	12.883	50.886	37.419	64.761	80.870	44.324 (35.63-53.44)	30.963 (20.46-41.84)	58.672 (43.96-73.87)
65-69	40.252	62.953	41.649	124.481	49.497	63.886	96.933	130.596	96.925	79.726 (63.03-98.62)	63.530 (40.81-85.50)	97.499 (68.24-127.94)
70-74	68.131	34.096	35.483	87.119	91.366	56.155	77.150	170.923	75.961	74.354 (54.96-93.44)	62.175 (37.77-82.20)	94.101 (57.73-133.28)
75-79	31.909	33.044	17.538	51.444	102.283	154.183	156.171	127.797	174.621	90.037 (64.85-112.64)	46.560 (25.98-72.16)	152.923 (104.38-204.58)
80-84	25.436	26.759	99.848	113.392	57.040	77.149	54.999	55.706	28.880	60.195 (37.49-84.97)	64.010 (29.89-93.93)	54.810 (24.11-90.42)

© 2023 Chien et al. *JAMA Network Open.*

(4) ever-smoking male

Age	Ever-smoking Male														
	2011	2012	2013	2014	2015	2016	2017	2018	2019	2011-2019		2011-2015		2016-2019	
	rate									rate	95% CI	rate	95% CI	rate	95% CI
40-44	1.260	1.808	0.992	1.721	3.847	2.090	3.879	3.795	3.831	2.466	(2.03-2.96)	1.869	(1.35-2.39)	3.379	(2.53-4.30)
45-49	2.535	5.938	3.142	4.307	4.383	8.148	5.651	6.731	8.430	5.337	(4.68-6.04)	4.047	(3.27-4.75)	7.230	(5.94-8.48)
50-54	5.117	6.484	8.444	8.235	11.222	11.579	11.614	11.410	14.237	9.795	(8.89-10.78)	7.917	(6.88-8.93)	12.196	(10.70-13.71)
55-59	12.310	10.960	11.880	14.621	16.073	19.919	22.602	24.980	23.374	17.468	(16.22-18.74)	13.187	(11.58-14.79)	22.723	(20.54-24.81)
60-64	20.246	23.285	26.898	26.793	29.485	29.254	36.839	34.209	41.362	30.276	(28.22-32.28)	25.600	(22.92-28.08)	35.441	(32.51-38.41)
65-69	28.446	44.448	32.567	34.617	47.407	39.651	51.132	49.645	59.323	44.244	(41.31-47.05)	37.712	(33.88-41.61)	50.327	(45.96-54.58)
70-74	50.512	55.866	50.611	55.677	68.790	66.822	86.829	73.906	91.917	65.575	(60.92-69.88)	56.031	(50.60-61.69)	79.808	(71.95-87.87)
75-79	50.946	66.812	53.782	77.772	83.064	79.146	84.757	83.539	90.862	73.789	(68.23-79.33)	66.033	(59.00-73.14)	84.461	(74.79-92.96)
80-84	55.520	65.946	78.953	83.392	80.544	91.086	117.879	84.829	82.605	80.311	(73.05-87.39)	71.978	(63.91-80.52)	94.359	(81.89-106.41)

© 2023 Chien et al. *JAMA Network Open.*

eTable 32C. Stage 3&4 lung cancer age-specific incidence rates for each single year and for the period 2011—2019 by sex and smoking status: never-smoking female (1), never-smoking male (2), ever-smoking female (3), ever-smoking male (4).

(1) never-smoking female

Age	Never-smoking Female														
	2011	2012	2013	2014	2015	2016	2017	2018	2019	2011-2019		2011-2015		2016-2019	
	rate									rate	95% Cl	rate	95% Cl	rate	95% Cl
40-44	9.169	8.021	7.550	7.413	7.123	5.749	5.213	7.739	8.719	7.421	(6.83-7.99)	7.861	(7.05-8.75)	6.889	(5.97-7.76)
45-49	17.125	14.311	16.732	16.647	13.797	11.715	17.348	15.303	13.836	15.212	(14.31-16.09)	15.727	(14.52-16.95)	14.549	(13.26-15.89)
50-54	29.520	25.404	25.021	25.867	25.020	25.198	21.776	24.829	23.450	25.108	(23.94-26.14)	26.145	(24.56-27.69)	23.815	(22.16-25.44)
55-59	39.350	39.316	39.333	44.020	36.272	38.116	37.563	35.122	38.786	38.608	(37.22-40.04)	39.654	(37.80-41.77)	37.394	(35.43-39.45)
60-64	62.184	61.837	60.181	64.030	65.555	59.032	54.520	60.313	56.921	60.334	(58.52-62.30)	62.870	(59.90-65.64)	57.695	(54.90-60.56)
65-69	81.792	84.239	77.462	82.785	71.909	83.317	76.780	79.774	70.695	78.221	(75.56-80.93)	79.338	(75.12-83.27)	77.293	(73.54-80.76)
70-74	103.266	121.012	117.799	108.155	100.995	110.615	110.219	111.393	110.917	110.515	(106.56-114.42)	110.285	(104.61-115.44)	110.797	(105.14-116.47)
75-79	140.541	161.273	138.674	144.421	160.101	140.113	153.270	133.309	137.621	145.280	(140.57-150.37)	149.152	(142.35-155.84)	141.026	(134.00-147.76)
80-84	187.602	200.664	161.230	192.099	198.584	179.734	179.515	192.572	176.877	185.265	(178.99-192.22)	188.137	(178.46-197.69)	182.188	(171.91-191.63)

© 2023 Chien et al. *JAMA Network Open.*

(2) never-smoking male

Age	Never-smoking Male														
	2011	2012	2013	2014	2015	2016	2017	2018	2019	2011-2019		2011-2015		2016-2019	
	rate									rate	95% CI	rate	95% CI	rate	95% CI
40-44	7.698	5.852	5.678	5.902	6.060	3.834	6.151	5.314	4.953	5.635	(4.89-6.38)	6.207	(5.11-7.34)	5.084	(4.06-6.16)
45-49	11.793	9.248	10.128	10.706	11.804	10.407	11.831	10.673	13.022	11.098	(9.87-12.17)	10.743	(9.28-12.12)	11.498	(9.81-13.08)
50-54	20.709	23.149	18.995	17.282	15.585	19.296	17.795	18.302	18.486	18.814	(17.36-20.25)	19.095	(17.12-20.96)	18.471	(16.13-20.56)
55-59	34.196	33.985	31.058	32.409	31.623	27.067	26.002	32.914	27.428	30.596	(28.78-32.55)	32.611	(29.92-35.24)	28.383	(25.60-31.20)
60-64	51.822	49.194	51.859	41.739	51.761	52.196	45.963	52.277	49.449	49.554	(47.14-52.24)	49.138	(45.31-52.93)	49.964	(46.12-53.61)
65-69	78.151	92.164	73.059	83.100	79.396	87.232	69.358	76.834	76.138	78.728	(74.56-82.64)	81.035	(74.48-87.47)	77.022	(71.52-82.55)
70-74	128.136	110.541	114.738	107.135	100.935	98.332	93.473	88.730	85.707	101.585	(96.46-106.96)	111.716	(103.78-119.51)	91.150	(84.11-98.86)
75-79	190.150	191.696	163.513	165.866	156.658	147.279	155.573	149.254	132.748	158.976	(150.86-166.73)	172.650	(160.32-184.26)	145.886	(136.17-156.17)
80-84	239.807	217.320	210.593	200.262	190.192	213.452	191.024	192.634	157.529	200.498	(190.84-210.68)	211.345	(197.32-224.93)	187.836	(173.70-202.75)

© 2023 Chien et al. *JAMA Network Open.*

(3) ever-smoking female

Age	Ever-smoking Female											
	2011	2012	2013	2014	2015	2016	2017	2018	2019	2011-2019	2011-2015	2016-2019
	rate									rate 95% CI	rate 95% CI	rate 95% CI
40-44	11.055	21.254	9.413	19.857	19.838	15.705	18.329	21.056	31.770	18.773 (15.13-22.03)	16.253 (12.25-20.32)	21.855 (16.81-27.41)
45-49	30.136	28.787	31.879	33.518	36.034	53.803	31.351	38.043	41.292	36.042 (30.87-40.90)	32.049 (25.13-38.56)	41.131 (32.03-49.58)
50-54	43.982	71.983	63.717	88.161	76.851	92.851	75.384	77.837	106.521	77.287 (68.66-86.07)	68.995 (58.11-79.10)	88.049 (73.65-102.65)
55-59	65.269	62.369	105.644	135.277	104.488	120.555	149.831	144.284	119.239	110.835 (98.07-123.20)	94.233 (78.37-108.23)	133.530 (113.93-156.45)
60-64	140.736	181.863	145.341	178.845	147.966	166.020	182.882	147.256	230.998	170.228 (152.63-187.77)	159.216 (136.69-184.11)	182.053 (155.80-207.70)
65-69	233.508	249.536	243.724	301.125	411.579	430.045	329.084	362.697	365.172	328.054 (291.81-366.00)	288.809 (240.95-336.16)	371.121 (313.46-426.48)
70-74	394.562	358.738	358.963	276.321	373.110	481.330	411.932	396.761	602.765	398.875 (351.81-443.84)	353.194 (299.92-408.78)	472.942 (396.24-558.34)
75-79	477.087	335.098	493.900	436.484	487.962	453.974	383.208	561.966	559.589	462.240 (409.58-513.69)	445.599 (375.25-519.57)	486.309 (396.63-576.05)
80-84	715.282	663.501	707.675	620.534	690.925	525.517	499.907	722.192	400.428	620.941 (547.34-697.29)	679.870 (572.11-776.94)	537.742 (427.99-657.06)

© 2023 Chien et al. *JAMA Network Open.*

(4) ever-smoking male

Age	Ever-smoking Male														
	2011	2012	2013	2014	2015	2016	2017	2018	2019	2011-2019		2011-2015		2016-2019	
	rate									rate	95% CI	rate	95% CI	rate	95% CI
40-44	16.560	16.136	16.707	17.412	12.257	11.554	15.612	17.641	18.687	15.851	(14.68-16.99)	15.895	(14.30-17.54)	15.784	(14.02-17.56)
45-49	23.824	28.931	30.022	32.017	27.805	33.880	34.071	32.705	27.131	29.903	(28.22-31.59)	28.439	(26.42-30.47)	32.053	(29.50-34.58)
50-54	46.906	52.034	51.252	52.425	52.730	51.924	51.314	56.813	53.487	52.086	(50.04-54.15)	51.087	(48.47-53.89)	53.363	(50.36-56.57)
55-59	88.000	87.082	91.476	92.699	92.372	88.997	103.541	101.766	110.087	95.180	(92.11-98.31)	90.349	(86.33-94.11)	101.110	(96.36-105.74)
60-64	152.574	159.836	158.830	143.728	150.395	154.379	150.171	157.743	174.342	155.885	(151.59-160.17)	152.874	(146.84-159.18)	159.210	(152.50-165.94)
65-69	245.903	222.406	215.953	236.737	246.887	233.843	233.897	235.902	255.075	237.137	(230.25-243.58)	233.998	(224.74-244.07)	240.061	(230.35-249.77)
70-74	421.746	388.081	404.129	410.628	392.779	426.305	430.708	423.779	412.151	411.520	(399.51-423.33)	403.685	(387.78-418.28)	423.206	(404.41-442.23)
75-79	540.969	540.018	513.478	516.135	557.937	546.541	531.108	551.014	584.144	541.752	(526.84-555.72)	533.786	(515.15-553.67)	552.712	(530.12-576.21)
80-84	684.059	642.854	672.604	700.517	740.592	710.195	738.774	710.687	695.396	696.264	(676.15-716.90)	685.702	(657.97-710.69)	714.071	(679.24-748.38)

© 2023 Chien et al. *JAMA Network Open.*

eTable 33A. Age-specific late-stage lung incidence rate ratios and their 95% confidence intervals comparing sex (1), smoking status (2), periods (3).

(1) sex

Age	Never-smoking Female/Male, 2011-2019		Ever-smoking Female/Male, 2011-2019	
	IRR	95% CI	IRR	95% CI
40-44	1.292	(1.13-1.58)	1.171	(0.94-1.36)
45-49	1.393	(1.26-1.54)	1.207	(1.00-1.36)
50-54	1.334	(1.22-1.46)	1.481	(1.34-1.66)
55-59	1.278	(1.19-1.36)	1.153	(1.03-1.29)
60-64	1.213	(1.14-1.27)	1.085	(0.98-1.19)
65-69	1.005	(0.95-1.05)	1.367	(1.17-1.53)
70-74	1.079	(1.02-1.17)	0.936	(0.84-1.04)
75-79	0.903	(0.85-0.96)	0.848	(0.75-0.95)
80-84	0.917	(0.87-0.96)	0.866	(0.76-0.99)

Age	Never-smoking Female/Male, 2011-2015		Ever-smoking Female/Male, 2011-2015	
	IRR	95% CI	IRR	95% CI
40-44	1.218	(0.98-1.58)	1.026	(0.79-1.33)
45-49	1.470	(1.32-1.76)	1.120	(0.89-1.39)
50-54	1.360	(1.22-1.56)	1.337	(1.08-1.49)
55-59	1.219	(1.10-1.37)	1.032	(0.85-1.20)
60-64	1.265	(1.16-1.40)	1.041	(0.92-1.20)
65-69	0.979	(0.89-1.08)	1.212	(1.03-1.47)

© 2023 Chien et al. *JAMA Network Open.*

Age	IRR	95% CI	IRR	95% CI
70-74	0.977	(0.89-1.06)	0.849	(0.73-1.00)
75-79	0.851	(0.78-0.93)	0.824	(0.71-0.93)
80-84	0.872	(0.79-0.95)	0.972	(0.85-1.13)

Age	Never-smoking Female/Male, 2016-2019		Ever-smoking Female/Male, 2016-2019	
	IRR	95% CI	IRR	95% CI
40-44	1.366	(1.02-1.85)	1.357	(1.00-1.89)
45-49	1.304	(1.08-1.59)	1.292	(1.07-1.57)
50-54	1.300	(1.13-1.49)	1.661	(1.31-1.88)
55-59	1.351	(1.22-1.53)	1.310	(1.07-1.52)
60-64	1.161	(1.06-1.29)	1.130	(0.98-1.28)
65-69	1.025	(0.92-1.11)	1.537	(1.26-1.83)
70-74	1.208	(1.11-1.31)	1.074	(0.88-1.21)
75-79	0.958	(0.88-1.04)	0.884	(0.69-1.07)
80-84	0.979	(0.89-1.09)	0.719	(0.56-0.87)

(2) smoking status

Age	Ever/never smoking Female, 2011-2019		Ever/never smoking Male, 2011-2019	
	IRR	95% CI	IRR	95% CI
40-44	2.520	(1.99-3.10)	2.780	(2.42-3.20)
45-49	2.339	(1.95-2.71)	2.699	(2.44-3.05)
50-54	3.069	(2.75-3.54)	2.765	(2.52-3.02)
55-59	2.832	(2.51-3.17)	3.137	(2.92-3.38)

© 2023 Chien et al. *JAMA Network Open.*

60-64	2.805	(2.47-3.11)	3.137	(2.98-3.32)
65-69	4.124	(3.64-4.61)	3.031	(2.87-3.24)
70-74	3.535	(3.02-3.99)	4.074	(3.86-4.31)
75-79	3.234	(2.86-3.73)	3.443	(3.29-3.66)
80-84	3.340	(2.85-3.76)	3.538	(3.35-3.71)

Age	Ever/never smoking Female, 2011-2015		Ever/never smoking Male, 2011-2015	
	IRR	95% CI	IRR	95% CI
40-44	2.140	(1.56-2.81)	2.539	(2.03-3.22)
45-49	1.988	(1.65-2.40)	2.609	(2.27-2.99)
50-54	2.642	(2.21-3.12)	2.687	(2.40-3.15)
55-59	2.375	(1.96-2.78)	2.805	(2.59-3.02)
60-64	2.540	(2.10-2.92)	3.087	(2.76-3.34)
65-69	3.602	(3.05-4.32)	2.909	(2.66-3.12)
70-74	3.169	(2.64-3.71)	3.648	(3.40-4.03)
75-79	3.031	(2.45-3.61)	3.133	(2.89-3.35)
80-84	3.629	(3.01-4.36)	3.255	(3.05-3.50)

Age	Ever/never smoking Female, 2016-2019		Ever/never smoking Male, 2016-2019	
	IRR	95% CI	IRR	95% CI
40-44	3.030	(2.23-3.85)	3.048	(2.44-4.05)
45-49	2.820	(2.15-3.62)	2.845	(2.48-3.32)
50-54	3.660	(3.03-4.32)	2.865	(2.53-3.37)
55-59	3.469	(2.86-4.02)	3.577	(3.27-4.01)

© 2023 Chien et al. *JAMA Network Open.*

60-64	3.110	(2.66-3.66)	3.194	(2.89-3.52)
65-69	4.697	(4.00-5.54)	3.132	(2.90-3.36)
70-74	4.130	(3.33-4.82)	4.645	(4.22-5.04)
75-79	3.513	(2.88-4.12)	3.810	(3.50-4.12)
80-84	2.909	(2.40-3.63)	3.958	(3.58-4.27)

(3) periods

Age	Never-smoking Female, 2016-2019/2011-2015		Never-smoking Male, 2016-2019/2011-2015	
	**IRR**	**95% CI**	**IRR**	**95% CI**
40-44	0.911	(0.77-1.05)	0.812	(0.63-1.12)
45-49	0.929	(0.83-1.05)	1.048	(0.87-1.25)
50-54	0.928	(0.85-1.01)	0.971	(0.81-1.10)
55-59	0.962	(0.90-1.04)	0.868	(0.74-0.97)
60-64	0.927	(0.87-0.98)	1.011	(0.91-1.14)
65-69	0.994	(0.91-1.06)	0.950	(0.87-1.05)
70-74	1.009	(0.95-1.08)	0.816	(0.74-0.91)
75-79	0.947	(0.89-1.01)	0.842	(0.76-0.93)
80-84	0.975	(0.90-1.03)	0.868	(0.81-0.96)
**Age**	**Ever-smoking Female, 2016-2019/2011-2015**		**Ever-smoking Male, 2016-2019/2011-2015**	
	**IRR**	**95% CI**	**IRR**	**95% CI**
40-44	1.290	(0.87-1.91)	0.975	(0.83-1.13)
45-49	1.318	(0.96-1.75)	1.143	(1.02-1.28)

© 2023 Chien et al. *JAMA Network Open.*

50-54	1.285	(1.02-1.54)	1.035	(0.97-1.13)
55-59	1.405	(1.17-1.68)	1.107	(1.02-1.18)
60-64	1.136	(0.94-1.43)	1.046	(1.00-1.12)
65-69	1.297	(1.06-1.58)	1.022	(0.96-1.08)
70-74	1.315	(1.05-1.63)	1.039	(0.99-1.09)
75-79	1.098	(0.88-1.43)	1.023	(0.97-1.08)
80-84	0.782	(0.58-1.00)	1.056	(0.99-1.12)

© 2023 Chien et al. *JAMA Network Open.*

eTable 33B. Age-specific early-stage lung incidence rate ratios and their 95% confidence intervals comparing sex (1), smoking status (2), periods (3).

(1) sex

Age	Never-smoking Female/Male, 2011-2019		Ever-smoking Female/Male, 2011-2019	
	IRR	95% CI	IRR	95% CI
40-44	1.676	(1.43-2.00)	2.617	(1.88-4.41)
45-49	1.548	(1.36-1.77)	2.187	(1.62-2.82)
50-54	1.601	(1.45-1.85)	2.345	(1.80-2.95)
55-59	1.586	(1.43-1.80)	1.923	(1.39-2.38)
60-64	1.551	(1.40-1.71)	1.464	(1.16-1.81)
65-69	1.397	(1.26-1.51)	1.802	(1.39-2.31)
70-74	1.180	(1.07-1.30)	1.134	(0.84-1.59)
75-79	1.173	(1.06-1.35)	1.220	(0.84-1.62)
80-84	1.020	(0.87-1.16)	0.750	(0.40-1.14)

Age	Never-smoking Female/Male, 2011-2015		Ever-smoking Female/Male, 2011-2015	
	IRR	95% CI	IRR	95% CI
40-44	1.599	(1.18-2.47)	1.995	(0.80-3.35)
45-49	1.383	(1.14-1.79)	1.690	(1.15-2.47)
50-54	1.585	(1.30-1.94)	1.885	(1.28-2.60)
55-59	1.312	(1.13-1.53)	1.624	(1.18-2.15)
60-64	1.493	(1.27-1.69)	1.210	(0.85-1.59)
65-69	1.464	(1.15-1.79)	1.685	(1.10-2.32)

© 2023 Chien et al. *JAMA Network Open.*

70-74	1.104	(0.92-1.29)	1.110	(0.67-1.61)
75-79	1.018	(0.85-1.20)	0.705	(0.33-1.14)
80-84	0.784	(0.61-0.99)	0.889	(0.41-1.35)

Age	Never-smoking Female/Male, 2016-2019		Ever-smoking Female/Male, 2016-2019	
	IRR	95% CI	IRR	95% CI
40-44	1.827	(1.37-2.43)	2.897	(1.62-4.50)
45-49	1.733	(1.46-2.06)	2.466	(1.67-3.70)
50-54	1.619	(1.42-1.89)	2.740	(1.93-3.76)
55-59	1.813	(1.61-2.01)	2.211	(1.62-2.82)
60-64	1.606	(1.43-1.78)	1.656	(1.28-2.16)
65-69	1.402	(1.27-1.53)	1.937	(1.38-2.65)
70-74	1.278	(1.11-1.44)	1.179	(0.66-1.62)
75-79	1.332	(1.15-1.63)	1.811	(1.29-2.37)
80-84	1.298	(1.04-1.71)	0.581	(0.28-1.03)

(2) smoking status

Age	Ever/never smoking Female, 2011-2019		Ever/never smoking Male, 2011-2019	
	IRR	95% CI	IRR	95% CI
40-44	1.005	(0.69-1.34)	0.643	(0.50-0.85)
45-49	1.082	(0.79-1.40)	0.766	(0.65-0.94)
50-54	1.206	(0.98-1.50)	0.823	(0.74-0.97)
55-59	1.288	(1.03-1.52)	1.063	(0.95-1.20)

© 2023 Chien et al. *JAMA Network Open.*

60-64	1.238	(0.95-1.58)	1.312	(1.20-1.48)
65-69	1.749	(1.34-2.19)	1.355	(1.22-1.52)
70-74	1.800	(1.29-2.25)	1.874	(1.66-2.12)
75-79	2.112	(1.51-2.63)	2.031	(1.79-2.28)
80-84	2.031	(1.08-3.00)	2.763	(2.36-3.32)

**Age**	**Ever/never smoking Female, 2011-2015**		**Ever/never smoking Male, 2011-2015**	
	**IRR**	**95% CI**	**IRR**	**95% CI**
40-44	0.922	(0.46-1.44)	0.739	(0.44-1.24)
45-49	0.844	(0.39-1.30)	0.691	(0.51-0.93)
50-54	1.125	(0.74-1.48)	0.946	(0.78-1.18)
55-59	1.232	(0.81-1.55)	0.995	(0.82-1.18)
60-64	1.151	(0.74-1.63)	1.421	(1.20-1.59)
65-69	2.010	(1.23-2.78)	1.747	(1.51-2.11)
70-74	1.868	(1.25-2.63)	1.859	(1.62-2.18)
75-79	1.365	(0.62-2.11)	1.971	(1.68-2.37)
80-84	2.710	(1.51-4.41)	2.388	(1.98-2.96)

**Age**	**Ever/never smoking Female, 2016-2019**		**Ever/never smoking Male, 2016-2019**	
	**IRR**	**95% CI**	**IRR**	**95% CI**
40-44	1.052	(0.69-1.52)	0.664	(0.46-0.90)
45-49	1.252	(0.93-1.73)	0.880	(0.70-1.15)
50-54	1.273	(0.96-1.62)	0.752	(0.64-0.93)
55-59	1.389	(1.10-1.73)	1.139	(1.01-1.36)

© 2023 Chien et al. *JAMA Network Open.*

60-64	1.302	(0.97-1.68)	1.263	(1.12-1.46)
65-69	1.704	(1.30-2.32)	1.233	(1.07-1.37)
70-74	1.841	(1.30-2.51)	1.995	(1.72-2.36)
75-79	2.941	(1.89-4.04)	2.163	(1.72-2.63)
80-84	1.518	(0.59-2.64)	3.392	(2.61-4.50)

(3) periods

Age	Never-smoking Female, 2016-2019/2011-2015		Never-smoking Male, 2016-2019/2011-2015	
	**IRR**	**95% CI**	**IRR**	**95% CI**
40-44	2.299	(1.94-2.83)	2.012	(1.41-3.00)
45-49	1.757	(1.51-2.04)	1.402	(1.03-1.85)
50-54	1.980	(1.75-2.19)	1.937	(1.54-2.40)
55-59	2.081	(1.88-2.38)	1.506	(1.30-1.81)
60-64	1.675	(1.55-1.85)	1.557	(1.33-1.84)
65-69	1.810	(1.62-2.00)	1.891	(1.64-2.16)
70-74	1.536	(1.36-1.81)	1.327	(1.07-1.55)
75-79	1.525	(1.32-1.73)	1.166	(0.99-1.50)
80-84	1.528	(1.25-1.82)	0.923	(0.73-1.28)
**Age**	**Ever-smoking Female, 2016-2019/2011-2015**		**Ever-smoking Male, 2016-2019/2011-2015**	
	**IRR**	**95% CI**	**IRR**	**95% CI**
40-44	2.625	(1.81-6.21)	1.808	(1.30-2.82)
45-49	2.607	(1.56-5.38)	1.786	(1.43-2.35)

© 2023 Chien et al. *JAMA Network Open.*

50-54	2.240	(1.53-3.82)	1.541	(1.26-1.86)
55-59	2.346	(1.57-3.33)	1.723	(1.46-1.99)
60-64	1.895	(1.23-2.70)	1.384	(1.20-1.60)
65-69	1.535	(0.93-2.20)	1.335	(1.17-1.52)
70-74	1.513	(0.93-2.63)	1.424	(1.29-1.67)
75-79	3.284	(1.91-6.85)	1.279	(1.11-1.52)
80-84	0.856	(0.36-2.67)	1.311	(1.06-1.55)

© 2023 Chien et al. *JAMA Network Open.*

eTable 33C. Age-specific late-stage 3&4 lung incidence rate ratios and their 95% confidence intervals comparing sex (1), smoking status (2), periods (3).

(1) sex

Age	Never-smoking Female/Male, 2011-2019		Ever-smoking Female/Male, 2011-2019	
	IRR	95% CI	IRR	95% CI
40-44	1.317	(1.14-1.59)	1.184	(0.89-1.39)
45-49	1.371	(1.24-1.54)	1.205	(0.99-1.40)
50-54	1.335	(1.21-1.45)	1.484	(1.33-1.70)
55-59	1.262	(1.18-1.35)	1.164	(1.01-1.29)
60-64	1.218	(1.14-1.29)	1.092	(1.00-1.21)
65-69	0.994	(0.94-1.04)	1.383	(1.23-1.52)
70-74	1.088	(1.02-1.17)	0.969	(0.85-1.14)
75-79	0.914	(0.86-0.97)	0.853	(0.75-0.97)
80-84	0.924	(0.88-0.98)	0.892	(0.79-1.01)

Age	Never-smoking Female/Male, 2011-2015		Ever-smoking Female/Male, 2011-2015	
	IRR	95% CI	IRR	95% CI
40-44	1.267	(1.00-1.61)	1.022	(0.74-1.34)
45-49	1.464	(1.31-1.70)	1.127	(0.88-1.43)
50-54	1.369	(1.22-1.61)	1.351	(1.12-1.59)
55-59	1.216	(1.09-1.35)	1.043	(0.89-1.22)
60-64	1.279	(1.17-1.42)	1.041	(0.86-1.22)
65-69	0.979	(0.90-1.10)	1.234	(1.02-1.44)

© 2023 Chien et al. *JAMA Network Open.*

70-74	0.987	(0.89-1.07)	0.875	(0.75-1.02)
75-79	0.864	(0.79-0.95)	0.835	(0.69-0.96)
80-84	0.890	(0.80-0.98)	0.991	(0.82-1.19)

Age	Never-smoking Female/Male, 2016-2019		Ever-smoking Female/Male, 2016-2019	
	IRR	95% CI	IRR	95% CI
40-44	1.355	(1.01-1.87)	1.385	(0.97-1.98)
45-49	1.265	(1.04-1.55)	1.283	(1.04-1.63)
50-54	1.289	(1.13-1.49)	1.650	(1.31-1.88)
55-59	1.317	(1.19-1.49)	1.321	(1.08-1.60)
60-64	1.155	(1.05-1.29)	1.143	(1.00-1.35)
65-69	1.004	(0.90-1.09)	1.546	(1.32-1.79)
70-74	1.216	(1.11-1.33)	1.118	(0.93-1.38)
75-79	0.967	(0.88-1.06)	0.880	(0.68-1.05)
80-84	0.970	(0.88-1.09)	0.753	(0.61-0.93)

(2) smoking status

Age	Ever/never smoking Female, 2011-2019		Ever/never smoking Male, 2011-2019	
	IRR	95% CI	IRR	95% CI
40-44	2.530	(2.10-3.12)	2.813	(2.42-3.27)
45-49	2.369	(2.02-2.78)	2.694	(2.46-3.09)
50-54	3.078	(2.65-3.53)	2.768	(2.50-3.07)
55-59	2.871	(2.58-3.24)	3.111	(2.91-3.38)

© 2023 Chien et al. *JAMA Network Open.*

60-64	2.821	(2.58-3.12)	3.146	(2.97-3.34)
65-69	4.194	(3.71-4.77)	3.012	(2.85-3.22)
70-74	3.609	(3.10-4.04)	4.051	(3.83-4.30)
75-79	3.182	(2.82-3.53)	3.408	(3.24-3.62)
80-84	3.352	(2.75-3.77)	3.473	(3.29-3.64)

**Age**	**Ever/never smoking Female, 2011-2015**		**Ever/never smoking Male, 2011-2015**	
	**IRR**	**95% CI**	**IRR**	**95% CI**
40-44	2.068	(1.56-2.80)	2.561	(2.00-3.17)
45-49	2.038	(1.62-2.47)	2.647	(2.28-3.14)
50-54	2.639	(2.22-3.19)	2.675	(2.37-3.15)
55-59	2.376	(1.95-2.86)	2.770	(2.55-3.01)
60-64	2.532	(2.15-2.96)	3.111	(2.81-3.37)
65-69	3.640	(3.01-4.39)	2.888	(2.62-3.14)
70-74	3.203	(2.67-3.74)	3.613	(3.40-4.03)
75-79	2.988	(2.47-3.48)	3.092	(2.87-3.35)
80-84	3.614	(3.03-4.17)	3.244	(3.02-3.50)

**Age**	**Ever/never smoking Female, 2016-2019**		**Ever/never smoking Male, 2016-2019**	
	**IRR**	**95% CI**	**IRR**	**95% CI**
40-44	3.172	(2.21-4.16)	3.105	(2.46-4.09)
45-49	2.827	(2.27-3.63)	2.788	(2.41-3.21)
50-54	3.697	(3.03-4.29)	2.889	(2.51-3.41)
55-59	3.571	(2.97-4.17)	3.562	(3.24-4.02)

© 2023 Chien et al. *JAMA Network Open.*

60-64	3.155	(2.58-3.78)	3.187	(2.88-3.53)
65-69	4.801	(4.06-5.63)	3.117	(2.86-3.38)
70-74	4.269	(3.33-5.13)	4.643	(4.21-5.13)
75-79	3.448	(2.90-4.16)	3.789	(3.47-4.12)
80-84	2.952	(2.37-3.51)	3.802	(3.41-4.13)

(3) periods

**Age**	**Never-smoking Female, 2016-2019/2011-2015**		**Never-smoking Male, 2016-2019/2011-2015**	
	**IRR**	**95% CI**	**IRR**	**95% CI**
40-44	0.876	(0.72-1.01)	0.819	(0.63-1.12)
45-49	0.925	(0.83-1.06)	1.070	(0.88-1.32)
50-54	0.911	(0.84-0.99)	0.967	(0.83-1.13)
55-59	0.943	(0.87-1.03)	0.870	(0.75-0.97)
60-64	0.918	(0.86-0.98)	1.017	(0.91-1.16)
65-69	0.974	(0.89-1.03)	0.950	(0.86-1.05)
70-74	1.005	(0.94-1.08)	0.816	(0.73-0.91)
75-79	0.946	(0.88-1.01)	0.845	(0.75-0.92)
80-84	0.968	(0.89-1.04)	0.889	(0.82-1.00)
**Age**	**Ever-smoking Female, 2016-2019/2011-2015**		**Ever-smoking Male, 2016-2019/2011-2015**	
	**IRR**	**95% CI**	**IRR**	**95% CI**
40-44	1.345	(0.92-2.16)	0.993	(0.86-1.17)
45-49	1.283	(0.94-1.70)	1.127	(1.00-1.26)

© 2023 Chien et al. *JAMA Network Open.*

50-54	1.276	(0.96-1.58)	1.045	(0.97-1.15)
55-59	1.417	(1.13-1.71)	1.119	(1.04-1.20)
60-64	1.143	(0.93-1.45)	1.041	(0.99-1.10)
65-69	1.285	(1.04-1.54)	1.026	(0.96-1.09)
70-74	1.339	(1.07-1.71)	1.048	(0.99-1.10)
75-79	1.091	(0.88-1.42)	1.035	(0.98-1.10)
80-84	0.791	(0.61-1.01)	1.041	(0.98-1.11)

© 2023 Chien et al. *JAMA Network Open.*

eReferences

1. Chiang CJ, You SL, Chen CJ, Yang YW, Lo WC, Lai MS. Quality assessment and improvement of nationwide cancer registration system in Taiwan: a review. Japanese journal of clinical oncology. 2015;45(3):291-6. doi: 10.1093/jjco/hyu211. PubMed PMID: 25601947.
2. Kao CW, Chiang CJ, Lin LJ, Huang CW, Lee WC, Lee MY, et al. Accuracy of long-form data in the Taiwan cancer registry. J Formos Med Assoc. 2021;120(11):2037-41. Epub 2021/05/23. doi: 10.1016/j.jfma.2021.04.022. PubMed PMID: 34020856.
3. Lu TH, Lee MC, Chou MC. Accuracy of cause-of-death coding in Taiwan: types of miscoding and effects on mortality statistics. International journal of epidemiology. 2000;29(2):336-43. PubMed PMID: 10817134.
4. Hsing AW, Ioannidis JP. Nationwide Population Science: Lessons From the Taiwan National Health Insurance Research Database. JAMA Intern Med. 2015;175(9):1527-9. doi: 10.1001/jamainternmed.2015.3540. PubMed PMID: 26192815.
5. Chien LH, Tseng TJ, Tsai FY, Wang JH, Hsiung CA, Liu TW, et al. Patterns of age-specific socioeconomic inequalities in net survival for common cancers in Taiwan, a country with universal health coverage. Cancer epidemiology. 2018;53:42-8. doi: 10.1016/j.canep.2018.01.006. PubMed PMID: 29396159.
6. Chien LH, Chen TY, Chen CH, Chen KY, Hsiao CF, Chang GC, et al. Recalibrating Risk Prediction Models by Synthesizing Data Sources: Adapting the Lung Cancer PLCO Model for Taiwan. Cancer Epidemiol Biomarkers Prev. 2022;31(12):2208-18. Epub 2022/09/22. doi: 10.1158/1055-9965.EPI-22-0281. PubMed PMID: 36129788.
7. Chien LH, Tseng TJ, Chen TY, Chen CH, Chen CY, Jiang HF, et al. Prevalence of comorbidities and their impact on survival among older adults with the five most common cancers in Taiwan: a population study. Sci Rep. 2023;13(1):6727. Epub 2023/05/15. doi: 10.1038/s41598-023-29582-0. PubMed PMID: 37185775; PubMed Central PMCID: PMCPMC10130096.
8. Chiang CY, Chang HY. A population study on the time trend of cigarette smoking, cessation, and exposure to secondhand smoking from 2001 to 2013 in Taiwan. Popul Health Metr. 2016;14:38. Epub 2016/11/09. doi: 10.1186/s12963-016-0109-x. PubMed PMID: 27822144; PubMed Central PMCID: PMCPMC5097365.
9. Efron B, Tibshirani RJ. An Introduction to the Bootstrap: Chapman and Hall/CRC; 1993.
10. Feng YA, Chen CY, Chen TT, Kuo PH, Hsu YH, Yang HI, et al. Taiwan Biobank:

© 2023 Chien et al. *JAMA Network Open.*

A rich biomedical research database of the Taiwanese population. Cell Genom. 2022;2(11):100197. Epub 2023/02/14. doi: 10.1016/j.xgen.2022.100197. PubMed PMID: 36776991; PubMed Central PMCID: PMCPMC9903657.
11. Chien LH, Chen CH, Chen TY, Chang GC, Tsai YH, Hsiao CF, et al. Predicting Lung Cancer Occurrence in Never-Smoking Females in Asia: TNSF-SQ, a Prediction Model. Cancer Epidemiol Biomarkers Prev. 2020;29(2):452-9. Epub 2019/12/19. doi: 10.1158/1055-9965.EPI-19-1221. PubMed PMID: 31848206.

© 2023 Chien et al. *JAMA Network Open.*
