# Supplementary material for: Incidence of Lung Adenocarcinoma by Age, Sex, and Smoking Status in Taiwan
Source: JAMA Netw Open. 2023 Nov 1;6(11):e2340704. doi: 10.1001/jamanetworkopen.2023.40704 (PMC10620613; doi:10.1001/jamanetworkopen.2023.40704)
Supplement: Supplement 2. — Data Sharing Statement [file jamanetwopen-e2340704-s002.pdf]

Data Sharing Statement

Chien. Incidence of Lung Adenocarcinoma by Age, Sex, and Smoking Status in Taiwan. *JAMA Netw Open*. Published November 01, 2023. doi:10.1001/jamanetworkopen.2023.40704

Data
Data available: No

Additional Information
Explanation for why data not available: The TCR, TCOD, NHIRD, and NHIS can be used for research upon approval of the Data Science Center, MOHW, Taiwan. The Taiwan Biobank dataset can be used for research upon approval of the Taiwan Biobank (https://taiwanview.twbiobank.org.tw/index). Age-, year- and sex-specific population sizes can be freely downloaded from MBIS, Ministry of Interior, Taiwan.
